# Predictors of treatment outcomes among patients with multidrug-resistant tuberculosis in Vietnam: a retrospective cohort study

**DOI:** 10.1186/s12879-021-06992-x

**Published:** 2022-01-20

**Authors:** Ian Wrohan, Thu Anh Nguyen, Viet Nhung Nguyen, Binh Hoa Nguyen, Thi Thanh Thuy Hoang, Phuong Chi Nguyen, Kavindhran Velen, Guy Barrington Marks, Greg James Fox

**Affiliations:** 1 The Central Clinical School, Faculty of Medicine and Health, The University of Sydney, 90-92 Parramatta Road, Sydney, NSW 2006 Australia; 2 The Woolcock Institute of Medical Research, Sydney, NSW 2037 Australia; 3 The Woolcock Institute of Medical Research, Hanoi, Vietnam; 4 National Lung Hospital, Hanoi, Vietnam; 5 South Western Sydney Clinical School, University of New South Wales, Sydney, NSW 2052 Australia

**Keywords:** MDR-TB, Gender, HIV, Co-morbidities, Bacteriological conversion

## Abstract

**Background:**

Improving treatment outcomes for multidrug-resistant tuberculosis (MDR-TB) is a leading priority for global TB control. This retrospective cohort study evaluated the factors associated with treatment success among patients treated for MDR-TB in two provinces in Vietnam.

**Methods:**

Treatment outcomes were evaluated for adult patients treated in Hanoi and Thanh Hoa provinces between 2014 and 2016. The primary outcome was the proportion of patients with treatment success, defined as cure or treatment completion. Logistic regression analysis was used to evaluate the relationship between patient clinical and microbiological characteristics and treatment success.

**Results:**

Treatment outcomes were reported in 612 of 662 patients; of these, 401 (65.5)% were successfully treated. The odds of treatment success were lower for male patients (aOR 0.56, 95% CI 0.34–0.90), for people living with HIV (aOR 0.44, 95% CI 0.20–1.00), and for patients treated for extensive antibiotic resistance (pre-XDR-/XDT-TB) (aOR 0.53, 95% CI 0.29–0.97), compared with others. Patients who achieved culture conversion in the first 4 months of treatment had increased odds (aOR 2.93, 95% CI 1.33–6.45) of treatment success. In addition, loss to follow-up was less common among patients covered by social health insurance compared to those who paid for treatment out-of-pocket (aOR 0.55, 95% CI 0.32–0.95).

**Conclusions:**

Among patients with MDR-TB, males, people living with HIV, and those with more extensive antibiotic resistance at diagnosis are at greatest risk of an unsuccessful treatment outcome. Efforts to optimise the management of co-morbidities (such as HIV), ensure rapid bacteriological conversion, and provide financial support for patients promise to improve treatment outcomes.

**Supplementary Information:**

The online version contains supplementary material available at 10.1186/s12879-021-06992-x.

## Background

Tuberculosis (TB) is the tenth leading cause of death worldwide. An estimated two billion people have been infected with *M. tuberculosis*, and of the 10 million people diagnosed with TB in 2019, 465,000 had rifampicin-resistant (RR-TB) or multidrug-resistant (MDR-TB) forms of the disease [1]. The treatment of drug-resistant tuberculosis is a prolonged and expensive process, further complicated by the occurrence of side effects resulting from the use of toxic drugs [2]. MDR-TB has a treatment success rate of only 58%, globally [1]. Consequently, drug-resistant TB represents a significant obstacle to realising the WHO End TB Strategy [3]. Vietnam ranks 11th among the top 20 high burden countries for MDR-TB, with 8400 new cases reported in 2019 [1]. In 2009, the Government of Vietnam initiated the Programmatic Management of Drug-resistant Tuberculosis (PMDT) to enhance diagnostic and treatment services for MDR-TB, and to provide free treatment with quality-assured drugs to patients across the country [4]. However despite this, less than 70% of patients enrolled in MDR-TB treatment achieve a successful outcome [1]. Re-treatment is a costly and time-consuming process, placing a strain on the healthcare system and often burdening families with catastrophic costs associated with treatment, despite government support [5]. Furthermore, re-treatment of TB has an even lower success rate than treatment among those receiving it for the first time [1, 6]. The aim of this study is to evaluate the factors associated with treatment success amongst patients treated for MDR-TB by the National Tuberculosis Programme (NTP) in two provinces of Vietnam. The findings of this study will inform the strengthening of programmatic management of drug-resistant TB in Vietnam.

## Methods

### Study setting

Vietnam is a Southeast Asian country with a population of 96 million people and a high annual TB incidence (176 cases of TB per 100,000 population). It is ranked among the high-burden countries for drug-resistant TB, with 8.8 cases of RR-/MDR-TB per 100,000 population. The Vietnamese healthcare system has four levels of care for managing MDR-TB patients (in descending order): Central, Provincial, District, and Commune. This study was conducted in two of Vietnam’s 63 provinces: Hanoi and Thanh Hoa, and included patients with confirmed RR-/MDR-TB who were treated at Provincial and District healthcare facilities within the Vietnamese PMDT program.

### Description of programmatic management of patients

At the time of this study, patients with presumptive MDR-TB were identified from among those presenting for the treatment of TB with risk factors for drug-resistance, including a history of prior treatment for TB, HIV infection, and exposure to individuals with known drug-resistant TB. Rifampicin resistance was confirmed using GeneXpert MTB/RIF and additional drug resistance was assessed using Genotype MTBDR*plus* and drug-susceptibility testing (DST). Once diagnosed, patients were treated at a provincial inpatient facility for up to 8 weeks and subsequent outpatient care was administered through district health centres. Standardised treatment for MDR-TB was delivered in accordance with WHO guidelines [4, 7]. During this study, 20-month standard and 9-month short-course regimens were used routinely to treat MDR-TB, although individualized regimens were used occasionally for patients with more extensive drug resistance (such as resistance to fluoroquinolones and second-line injectable drugs). Patients were requested to return to the provincial hospital for a monthly clinical evaluation, which included a chest radiograph along with sputum smear and culture analysis. Although most clinical costs were covered by the NTP, other expenses (e.g., transportation) were either self-funded or funded through the social health insurance (SHI) program, which covered at least 80% of a patient’s direct expenses [5].

### Study design and participants

Participants in this retrospective cohort study included consecutive adult (at least 15 years of age) patients who were diagnosed with either RR-TB or MDR-TB and registered for treatment in the PMDT program between January 01, 2014 and December 31, 2016. MDR-TB was defined as patients shown to be resistant to both isoniazid and rifampicin. This group also included patients with more extensive drug resistance, including extensively drug-resistant tuberculosis (XDR-TB) (additional resistance to both a fluoroquinolone and at least one injectable agent) and pre-XDR-TB (additional resistance to either a fluoroquinolone or injectable) [7]. Although study participants need not have been residents of either Hanoi or Thanh Hoa, they were required to have started treatment in one of these provinces.

### Data collection

Patient data were extracted from NTP patient registers, individual clinical records, and the Vietnam electronic TB database (eTB Manager). Ten percent of register entries and patient records were selected, at random, for duplicate data transcription in order to verify the accuracy of data entry. Source documents were transcribed into electronic case report forms developed using Epidata 4.4 (see Additional file 2). Data from multiple sources were linked based upon the following patient identifiers: name, age, gender, address, and treatment dates. Data collected from the source documents included patient demographics, pre-treatment information, treatment details (e.g., sputum smear and mycobacterial culture results), and treatment outcomes (see Additional file 1: Table S1). Treatment outcomes were assigned using standardised definitions [4, 7]: success, failure, loss to follow-up, death, or not evaluated (see Additional file 1: Table S2).

### Data analysis

Participant characteristics were evaluated using descriptive statistics. Logistic regression analysis was used to determine the associations between independent variables and treatment outcomes. Univariate analyses were first used to calculate crude (unadjusted) odds ratios (cOR) with a 95% confidence interval for each variable, and those variables individually associated with treatment success at a level of *p* ≤ 0.20 were then evaluated together in a multivariable logistic regression. Multiple imputation was used to address missing values in the dataset, based upon 50 imputations using the fully conditional specification method, with all 23 variables included in the univariate analyses used as predictors. Adjusted odds ratios (aOR) with a 95% confidence interval were calculated for each variable in the multivariable analysis, and those variables significantly (*p* ≤ 0.05) associated with treatment success were included in the final model. Multicollinearity between variables was evaluated using the variance inflation factor (VIF). Univariate analyses were also performed for treatment failure, loss to follow-up, and death. However, as the primary outcome of interest was treatment success, multivariable analyses were not performed for the unsuccessful outcomes. Participants for whom a treatment outcome was not evaluated (i.e., not reported), including patients who were transferred to another treatment unit, were not included in the primary analysis. However, to assess the range of impacts that these missing treatment outcomes may have had on the findings of this study, the upper and lower bounds of the estimated treatment success rate were calculated by assuming individuals without an evaluated outcome all had successful outcomes, or all had unsuccessful outcomes. All analyses and operations were done using IBM SPSS version 26 (IBM Corporation, NY).

### Ethical considerations

Ethical approval for the project was obtained though the Human Research Ethics Committee (HREC) at the University of Sydney in Australia (project no. 2018/746), and the Vietnam National Lung Hospital. Due to the retrospective nature of this study, the need for written informed consent was waived by the HREC at the University of Sydney, and consent to access and analyze the patient treatment data used in this study was obtained through the Vietnam National Lung Hospital. All patient data was stored in a secure database during data collection and de-identified prior to analysis to ensure the confidentiality of study participants. All methods were carried out in accordance with relevant guidelines and regulations.

## Results

### Participant characteristics

Treatment records for 662 individual patients with RR-/MDR-TB were retrieved and included in the study (Table 1). The 494 patients who were treated in Hanoi accounted for majority of the study population. Males comprised 76.4% of the population. Age ranged from 15 to 85 years with a median age of 43 years [interquartile range (IQR) 32–55]. Previous treatment for TB was reported in 532 (82.7%) of the 643 patients for whom data was available. Of the 458 patients who reported the method by which their treatment was financed, 333 (72.7%) were supported through social health insurance. Among 527 patients for whom HIV status was recorded, 37 (7.0%) were HIV-positive. Among 458 patients for whom other co-morbidity data were recorded, the most commonly reported co-morbidities were diabetes (11.4%) and other respiratory disorders (11.8%). Among the 645 patients for whom infection site data were recorded, 622 (96.4%) had pulmonary disease. Although rifampicin resistance was confirmed in all patients using GeneXpert MTB/RIF, additional drug susceptibility test results at the commencement of treatment were available for only 441 of the 662 patients. Among these, 388 (88.0%) were classified at MDR-TB, 36 (8.2%) as pre-XDR-TB, and 17 (3.8%) as XDR-TB.

**Table 1:** Patient characteristics

	Hanoi province	Thanh Hoa province	Total
**Total**	**494**	**168**	**662**
**Gender**	**n = 494**	**n = 168**	**n = 662**
Male	365 (73.9%)	141 (83.9%)	506 (76.4%)
Female	129 (26.1%)	27 (16.1%)	156 (23.6%)
**Age (years)**	**n = 494**	**n = 167**	**n = 661**
Mean (IQR)	43.0 (32.0–55.0)	44.0 (33.0–56.0)	43.0 (32.0–55.0)
By group (years)			
≤ 19	12 (2.4%)	1 (0.6%)	13 (2.0%)
20–39	204 (41.3%)	64 (38.3%)	268 (40.5%)
40–59	207 (41.9%)	71 (42.5%)	278 (42.1%)
60–79	69 (14.0%)	29 (17.4%)	98 (14.8%)
≥ 80	2 (0.4%)	2 (1.2%)	4 (0.6%)
Not reported	0	1	1
**Previous TB treatment**	**n = 478**	**n = 165**	**n = 643**
Yes	394 (82.4%)	138 (83.6%)	532 (82.7%)
No	84 (17.6%)	27 (16.4%)	111 (17.3%)
Not reported	16	3	19
**Financially supported through social health insurance**	**n = 311**	**n = 147**	**n = 458**
Yes	224 (72.0%)	109 (74.1%)	333 (72.7%)
No	87 (28.0%)	38 (25.9%)	125 (27.3%)
Not reported	183	21	204
**HIV status**	**n = 453**	**n = 74**	**n = 527**
Positive	32 (7.1%)	5 (6.8%)	37 (7.0%)
Negative	421 (92.9%)	69 (93.2%)	490 (93.0%)
Not reported	41	94	135
**Other comorbidities**	**n = 312**	**n = 146**	**n = 458**
Diabetes	44 (14.1%)	8 (5.5%)	52 (11.4%)
Heart disease	12 (3.8%)	6 (4.1%)	18 (3.9%)
Liver disease	13 (4.2%)	7 (4.8%)	20 (4.4%)
Kidney disease	2 (0.6%)	0 (0%)	2 (0.4%)
Malnutrition	7 (2.2%)	7 (4.8%)	14 (3.1%)
Psychiatric disorder	13 (4.2%)	0 (0%)	13 (2.8%)
Respiratory disorder^a^	32 (10.3%)	22 (15.1%)	54 (11.8%)
Substance abuse	8 (2.6%)	10 (6.8%)	18 (3.9%)
Other^b^	10 (3.2%)	3 (2.1%)	13 (2.8%)
Not reported	182	22	204
**Infection site**	**n = 481**	**n = 164**	**n = 645**
Pulmonary	458 (95.2%)	164 (100.0%)	622 (96.4%)
Extra-pulmonary	12 (2.5%)	0 (0%)	12 (1.9%)
Both	11 (2.3%)	0 (0%)	11 (1.7%)
Not reported	13	4	17
**Initial sputum smear grade**	**n = 474**	**n = 146**	**n = 620**
Negative	162 (34.2%)	66 (45.2%)	228 (36.8%)
Scanty	8 (1.7%)	6 (4.1%)	14 (2.3%)
1 +	185 (39.0%)	23 (15.8%)	208 (33.5%)
2 +	54 (11.4%)	26 (17.8%)	80 (12.9%)
3 +	65 (13.7%)	25 (17.1%)	90 (14.5%)
Not reported	20	22	42
**Antibiotic resistance at enrolment**	**n = 309**	**n = 132**	**n = 441**
RR-/MDR-TB only	257 (83.2%)	131 (98.5%)	388 (88.0%)
Pre-XDR-TB	35 (11.3%)	1 (1.5%)	36 (8.2%)
XDR-TB	17 (5.5%)	0 (0%)	17 (3.8%)
Not reported	185	36	221

Bolded numbers indicate the number of individuals for whom data were available (percentages exclude those for whom data were not reported)

^a^Respiratory disorder refers to either: atelectasis, bronchiectasis, bronchitis, bronchopneumonia, chronic pulmonary disease, lung tumor, pneumonia, pneumothorax, or respiratory failure

^b^Other comorbidity refers to either: adrenal failure, anemia, esophageal cancer, gout, pleural effusion, or seizure

### Treatment outcomes and adverse events

The median duration of inpatient stay was 28 days (IQR 16–37), and the median total treatment duration was 19 months (IQR 9.7–20.0) (Table 2). Sputum smear conversion within 2 months of starting treatment was observed in 503 (85.8%) of the 586 patients for whom smear results were available, and within 4 months in 519 (96.6%) of 537 patients. Culture conversion within 2 months of starting treatment was observed in 415 (75.0%) of the 553 patients for whom mycobacterial culture results were available, and within 4 months in 488 (92.4%) of 528 patients. Of the 452 patients for whom side effects during inpatient treatment were documented, 39 (8.6%) experienced one or more side effects. Of the 572 patients for whom side effects during outpatient treatment were recorded, 107 (18.7%) experienced one or more side effects. Common reported side effects included high uric acid levels (17.0%), hepatotoxicity (14.2%), and joint pain (14.2%).

**Table 2:** Microbiological outcomes and side effects for patients, by province

	Hanoi province	Thanh Hoa province	Total
**Total**	**n = 494**	**n = 168**	**n = 662**
**Treatment duration [median (IQR)]**			
Start to completion (months)	19.0 (9.8–20.0)	20.0 (9.7–21.3)	19.0 (9.7–20.0)
Inpatient regimen (days)	25 (16–34)	33 (19–42)	28 (16–37)
**Smear conversion after 2 months**	**n = 454**	**n = 132**	**n = 586**
Yes	418 (92.1%)	85 (64.4%)	503 (85.8%)
No	36 (7.9%)	47 (35.6%)	83 (14.2%)
Not reported	40	36	76
**Smear conversion after 4 months**	**n = 421**	**n = 116**	**n = 537**
Yes	409 (97.1%)	110 (94.8%)	519 (96.6%)
No	12 (2.9%)	6 (5.2%)	18 (3.4%)
Not reported	73	52	125
**Culture conversion after 2 months**	**n = 448**	**n = 105**	**n = 553**
Yes	405 (90.4%)	10 (9.5%)	415 (75.0%)
No	43 (9.6%)	95 (90.5%)	138 (25.0%)
Not reported	46	63	109
**Culture conversion after 4 months**	**n = 420**	**n = 108**	**n = 528**
Yes	402 (95.7%)	86 (79.6%)	488 (92.4%)
No	18 (4.3%)	22 (20.4%)	40 (7.6%)
Not reported	74	60	134
**Side effects during treatment**			
** At least one inpatient side effect reported**	**n = 308**	**n = 144**	**n = 452**
Yes	23 (7.5%)	16 (11.1%)	39 (8.6%)
No	285 (92.5%)	128 (88.9%)	413 (91.4%)
Not reported	186	24	210
** At least one outpatient side effect reported**	**n = 447**	**n = 125**	**n = 572**
Yes	75 (16.8%)	32 (25.6%)	107 (18.7%)
No	372 (83.2%)	93 (74.4%)	465 (81.3%)
Not reported	47	43	90
** Outpatient side effects reported**^**a**^	**n = 195**	**n = 52**	**n = 247**
Blood disorder	6 (3.1%)	0 (0%)	6 (2.4%)
Bowel pain	3 (1.5%)	2 (3.8%)	5 (2.0%)
Hearing loss	9 (4.6%)	3 (5.8%)	12 (4.9%)
Hepatotoxicity	35 (17.9%)	0 (0%)	35 (14.2%)
High blood sugar	10 (5.1%)	0 (0%)	10 (4.0%)
High uric acid	42 (21.5%)	0 (0%)	42 (17.0%)
Hypokalemia	15 (7.7%)	0 (0%)	15 (6.1%)
Joint pain	19 (9.7%)	16 (30.8%)	35 (14.2%)
Loss of appetite	11 (5.6%)	0 (0%)	11 (4.5%)
Nephrotoxicity	17 (8.7%)	0 (0%)	17 (6.9%)
Neurotoxicity	7 (3.6%)	1 (1.9%)	8 (3.2%)
Nausea	0 (0%)	23 (44.2%)	23 (9.3%)
Vision loss	7 (3.6%)	2 (3.8%)	9 (3.6%)
Vertigo	14 (7.2%)	5 (9.6%)	19 (7.7%)
Not reported	299	116	415

Bolded numbers indicate the number of individuals for whom data were available (percentages exclude those for whom data were not reported)

^a^Patients could report more than one side effect

Treatment success was reported in 401 (65.5%) of the 612 patients for whom treatment outcome was evaluated (Table 3). Of these, 96.0% were cured and 4.0% completed treatment. Of the 211 patients who did not achieve a successful outcome, 54 (8.2%) experienced treatment failure, 107 (16.2%) were lost to follow-up, and 50 (7.5%) patients died. Of the 50 (7.5%) patients for whom a final outcome was not available, 31 were transferred to other provinces. Additional file 1 (Tables S6 and S7) shows the characteristics of patients for whom a treatment outcome was ‘not evaluated’, compared with those for whom a treatment outcome was reported. Assuming that all 50 patients without an evaluated outcome had been successfully treated, the overall proportion of patients with treatment success could be as high as 68.1%. Alternatively, if these patients all had unsuccessful treatment, as few as 60.6% of patients may have been treated successfully.

**Table 3:** Patient treatment outcomes by province

	Hanoi province	Thanh Hoa province	Total
**Total**	**n = 494**	**n = 168**	**n = 662**
**Treatment outcomes**	**n = 494**	**n = 168**	**n = 662**
Cure	314 (63.6%)	71 (42.3%)	385 (58.2%)
Treatment completion	12 (2.4%)	4 (2.4%)	16 (2.4%)
Treatment failure	43 (8.7%)	11 (6.6%)	54 (8.2%)
Loss to follow-up	71 (14.4%)	36 (21.4%)	107 (16.2%)
Death	30 (6.1%)	20 (11.9%)	50 (7.5%)
Not evaluated	24 (4.8%)	26 (15.5%)	50 (7.5%)
Transferred out	10 (2.0%)	21 (12.5%)	31 (4.7%)
Unknown outcome	14 (2.8%)	5 (3.0%)	19 (2.8%)
**Treatment success**^**a**^	**n = 470**	**n = 142**	**n = 612**
Successful outcome (cure or completion)	326 (69.4%)	75 (52.3%)	401 (65.5%)
Unsuccessful outcome (failure, loss to follow-up, or death)	144 (30.6%)	67(47.7%)	211 (34.5%)

Bolded numbers indicate the number of individuals for whom data were available (percentages exclude those for whom data were not reported)

^a^Reported for patients in whom treatment outcomes were reported. Successful treatment was defined as an outcome of either ‘cured’ or ‘treatment completed’; Unsuccessful treatment was defined as ‘treatment failure’, ‘loss to follow-up’, or ‘death’. 24 patients from Hanoi and 26 patients from Thanh Hoa were lacking treatment outcomes

### Factors associated with treatment success

Univariate analyses identified 15 variables (Table 4) for which there was some evidence (at the level of *p* ≤ 0.20) of an association with a successful treatment outcome (cure or treatment completion). Only 230 of 612 cases were complete (containing data for all 15 variables) and multiple imputation was thus used to complete the dataset (see Additional file 3). The pooled value for each variable was derived from 50 imputations.

**Table 4:** Univariate analyses of treatment variables for the outcome of treatment success versus treatment failure, loss to follow-up, or death, using complete case analysis

Variable	Treatment outcome [n (%)]		Odds of treatment success versus failure, loss to follow-up, or death [cOR (95% CI)]	*p*-value
	Success	Failure, loss to follow-up, or death		
**Age (n = 612)**	**n/a**^a^	**n/a**^a^	**0.99 (0.97–1.00)**	**0.013**
**Gender (n = 612)**				
**Female**	**112 (75.7%)**	**36 (24.3%)**	**1.00 (reference)**	**0.003**
**Male**	**289 (62.3%)**	**175 (37.7%)**	**0.53 (0.35–0.81)**	
Previous treatment (n = 594)				
No	65 (62.5%)	39 (37.5%)	1.00 (reference)	0.456
Yes	325 (66.3%)	165 (33.7%)	1.18 (0.76–1.83)	
Financially supported through SHI (n = 426)				
No	72 (62.6%)	43 (37.4%)	1.00 (reference)	0.310
Yes	211 (67.8%)	100 (32.2%)	1.26 (0.81–1.97)	
**Diabetes (n = 424)**				
**No**	**256 (68.6%)**	**117 (31.4%)**	**1.00 (reference)**	**0.053**
**Yes**	**28 (54.9%)**	**23 (45.1%)**	**0.56 (0.31–1.01)**	
**Heart disease (n = 424)**				
**No**	**275 (67.7%)**	**131 (32.3%)**	**1.00 (reference)**	**0.125**
**Yes**	**9 (50.0%)**	**9 (50.0%)**	**0.48 (0.19–1.23)**	
**HIV (n = 500)**				
**No**	**319 (68.8%)**	**145 (31.2%)**	**1.00 (reference)**	**0.001**
**Yes**	**15 (41.7%)**	**21 (58.3%)**	**0.33 (0.16–0.65)**	
Kidney disease (n = 424)				
No	283 (67.1%)	139 (32.9%)	1.00 (reference)	0.616
Yes	1 (50.0%)	1 (50.0%)	0.49 (0.03–7.91)	
Liver disease (n = 424)				
No	273 (67.6%)	131 (32.4%)	1.00 (reference)	0.248
Yes	11 (55.0%)	9 (45.0%)	0.59 (0.24–1.45)	
Malnutrition (n = 424)				
No	277 (67.4%)	134 (32.6%)	1.00 (reference)	0.312
Yes	7 (53.8%)	6 (46.2%)	0.46 (0.19–1.71)	
Psychiatric disorder (n = 424)				
No	274 (66.5%)	138 (33.5%)	1.00 (reference)	0.237
Yes	10 (83.3%)	2 (16.7%)	2.52 (0.54–11.65)	
**Respiratory disorder**^**b**^ **(n = 424)**				
**No**	**256 (68.8%)**	**116 (31.2%)**	**1.00 (reference)**	**0.034**
**Yes**	**28 (53.8%)**	**24 (46.2%)**	**0.53 (0.29–0.95)**	
**Substance abuse (n = 424)**				
**No**	**277 (68.1%)**	**130 (31.9%)**	**1.00 (reference)**	**0.027**
**Yes**	**7 (41.2%)**	**10 (58.8%)**	**0.33 (0.12–0.88**)	
Other comorbidity^c^ (n = 424)				
No	277 (67.4%)	134 (32.6%)	1.00 (reference)	0.312
Yes	7 (53.8%)	6 (46.2%)	0.56 (0.19–1.71)	
Infection site (n = 598)				
Pulmonary	378 (65.4%)	200 (34.6%)	1.00 (reference)	0.777
Extrapulmonary	9 (75.0%)	3 (25.0%)	1.59 (0.43–5.92)	0.492
Both	5 (62.5%)	3 (37.5%)	0.88 (0.21–3.72)	0.864
**Antibiotic resistance (n = 409)**				
**RR-/MDR-TB**	**255 (71.4%)**	**102 (28.6%)**	**1.00 (reference)**	**0.000**
**Pre-XDR or XDR-TB**	**24 (47.2%)**	**28 (52.8%)**	**0.34 (0.19–0.62)**	
**Initial sputum (smear) positivity (n = 584)**				
**No**	**149 (69.6%)**	**65 (30.4%)**	**1.00 (reference)**	**0.152**
**Yes**	**235 (63.7%)**	**134 (36.3%)**	**0.77 (0.54–1.10)**	
**Smear conversion after 2 months (n = 557)**				
**No**	**41 (53.2%)**	**36 (46.8%)**	**1.00 (reference)**	**0.002**
**Yes**	**341 (71.0%)**	**139 (29.0%)**	**2.15 (1.32–3.51)**	
**Culture conversion after 2 months (n = 526)**				
**No**	**75 (62.5%)**	**45 (37.5%)**	**1.00 (reference)**	**0.071**
**Yes**	**289 (71.2%)**	**117 (28.8%)**	**1.48 (0.97–2.27)**	
**Smear conversion after 4 months (n = 518)**				
**No**	**6 (37.5%)**	**10 (62.5%)**	**1.00 (reference)**	**0.005**
**Yes**	**364 (72.5%)**	**138 (27.5%)**	**4.40 (1.57–12.33)**	
**Culture conversion after 4 months (n = 509)**				
**No**	**15 (44.1%)**	**19 (55.9%)**	**1.00 (reference)**	**0.000**
**Yes**	**351 (73.9%)**	**124 (26.1%)**	**3.59 (1.77–7.27)**	
**At least one side effect experienced during inpatient treatment (n = 418)**				
**No**	**261 (67.4%)**	**126 (32.6%)**	**1.00 (reference)**	**0.077**
**Yes**	**16 (51.6%)**	**15 (48.4%)**	**0.52 (0.25–1.08)**	
**At least one side effect experienced during outpatient treatment (n = 545)**				
**No**	**294 (66.2%)**	**150 (33.8%)**	**1.00 (reference)**	**0.120**
**Yes**	**75 (74.3%)**	**26 (25.7%)**	**1.47 (0.90–2.40)**	

cOR = crude (unadjusted) odds ratio

n = number of individuals for whom data were available

RR-/MDR-TB = rifampicin-resistant or multidrug-resistant tuberculosis

Pre-XDR or XDR-TB = pre-extensively drug resistant tuberculosis or extensively drug resistant tuberculosis

Bolded variables were included in the multivariable analysis

^a^Age is represented as a continuous variable in the model

^b^Respiratory disorder refers to either: atelectasis, bronchiectasis, bronchitis, bronchopneumonia, chronic pulmonary disease, lung tumor, pneumonia, pneumothorax, or respiratory failure

^c^Other includes: adrenal failure, anaemia, esophageal cancer, gout, pleural effusion, and a seizure disorder

Four variables were then found to be significantly (*p* ≤ 0.05), independently associated with treatment success in the multivariable analysis (see Additional file 1: Fig. S1). Male patients had lower odds of treatment success (aOR 0.56, 95% CI 0.34–0.90) compared to female patients, and patients also receiving treatment for HIV (aOR 0.44, 95% CI 0.20–1.00) had lower odds of treatment success compared to patients who were not also under treatment for HIV (Table 5). Patients treated for extensive antibiotic resistance (pre-XDR-/XDR-TB vs. other forms of RR-/MDR-TB) were less likely (aOR 0.53, 95% CI 0.29–0.97) to have a successful treatment outcome compared with RR-/MDR-TB cases. However, achieving bacteriological (culture) conversion in the first 4 months of treatment was associated with increased odds (aOR 2.93, 95% CI 1.33–6.45) of treatment success. There was no evidence of multicollinearity between variables in the multivariable model.

**Table 5:** Multivariable analysis of treatment variables for the outcome of treatment success versus treatment failure, loss to follow-up, or death

Variable	Treatment outcome [n (%)]		Odds of treatment success versus failure, loss to follow-up, or death [aOR (95% CI)]	*p*-value
	Success (n = 401)	Failure, loss to follow-up, or death (n = 211)		
Age	n/a^a^	n/a^a^	0.99 (0.98–1.00)	0.079
**Gender**				
**Female**	**112 (75.7%)**	**36 (24.3%)**	**1.00 (reference)**	**0.016**
**Male**	**289 (62.3%)**	**175 (37.7%)**	**0.56 (0.34–0.90)**	
Diabetes				
No	315 (67.5%)	152 (32.5%)	1.00 (reference)	0.286
Yes	86 (59.3%)	59 (40.7%)	0.72 (0.40–1.32)	
Heart disease				
No	339 (66.7%)	169 (33.3%)	1.00 (reference)	0.683
Yes	62 (59.6%)	42 (40.4%)	0.83 (0.33–2.05)	
**HIV**				
**No**	**377 (67.0%)**	**183 (33.0%)**	**1.00 (reference)**	**0.049**
**Yes**	**24 (46.2%)**	**28 (53.8%)**	**0.44 (0.20–1.00)**	
Respiratory disorder^b^				
No	337 (67.0%)	166 (33.0%)	1.00 (reference)	0.395
Yes	64 (58.7%)	45 (41.3%)	0.76 (0.41–1.42)	
Substance abuse				
No	339 (66.9%)	168 (33.1%)	1.00 (reference)	0.607
Yes	62 (59.0%)	43 (41.0%)	0.77 (0.29–2.09)	
**Antibiotic resistance**				
**RR/MDR-TB**^**c**^	**331 (68.0%)**	**156 (32.0%)**	**1.00 (reference)**	**0.039**
**Pre-XDR or XDR-TB**^**d**^	**70 (56.0%)**	**55 (44.0%)**	**0.53 (0.29–0.97)**	
Initial sputum (smear) positivity				
No	155 (69.2%)	69 (30.8%)	1.00 (reference)	0.429
Yes	246 (63.4%)	142 (36.6%)	0.85 (0.57–1.27)	
Smear conversion after 2 months				
No	45 (50.0%)	45 (50.0%)	1.00 (reference)	0.204
Yes	356 (68.2%)	166 (31.8%)	1.47 (0.81–2.67)	
Culture conversion after 2 months				
No	85 (58.2%)	61 (41.8%)	1.00 (reference)	0.361
Yes	316 (67.8%)	150 (32.2%)	1.28 (0.75–2.20)	
Smear conversion after 4 months				
No	12 (34.2%)	23 (65.8%)	1.00 (reference)	0.106
Yes	389 (67.4%)	188 (32.6%)	2.47 (0.82–7.39)	
**Culture conversion after 4 months**				
**No**	**22 (42.3%)**	**30 (57.7%)**	**1.00 (reference)**	**0.008**
**Yes**	**379 (67.7%)**	**181 (32.3%)**	**2.93 (1.33–6.45)**	
At least one side effect experienced during inpatient treatment				
No	336 (66.8%)	167 (33.2%)	1.00 (reference)	0.645
Yes	65 (59.6%)	44 (40.4%)	0.80 (0.32–2.05)	
At least one side effect experienced during outpatient treatment				
No	319 (64.2%)	178 (35.8%)	1.00 (reference)	0.104
Yes	82 (71.3%)	33 (28.7%)	1.54 (0.91–2.61)	

Bolded variables were significantly (*p* ≤ 0.05) associated with the outcome

^a^Age is represented as a continuous variable in the model

^b^Respiratory disorder refers to either: atelectasis, bronchiectasis, bronchitis, bronchopneumonia, chronic pulmonary disease, lung tumor, pneumonia, pneumothorax, or respiratory failure

^c^Rifampicin-resistant or multidrug-resistant tuberculosis

^d^Pre-extensively-resistant or extensively-resistant tuberculosis

## Discussion

This retrospective cohort study of 662 patients from two provinces of Vietnam found an overall treatment success rate of 65.5% and identified several important barriers to successful MDR-TB treatment. While treatment outcomes were influenced by a variety of demographic, financial, and clinical factors, a patient’s gender, HIV status, the extent of antibiotic resistance at diagnosis, and whether bacteriological conversion was achieved within 4 months of commencing treatment were most strongly associated with treatment success.

The reduced odds of treatment success among patients living with HIV in this study was comparable to other Vietnamese [8, 9] and global [10] estimates. Although the HIV burden in Vietnam is relatively low (compared with other high MDR-TB burden counties [1]), and affordable antiretroviral therapy is readily available [11–13], patients receiving concomitant treatment for HIV/TB may be at a greater risk of experiencing drug-related side effects compared with patients who are not being treated for HIV [7]. Patient discomfort resulting from severe side effects or pill burden can increase the chance of voluntary withdrawal from treatment, and this may be reflected in the considerable risk of loss to follow-up associated with patients also under treatment for HIV in this study (see Additional file 1: Table S3). The stigma associated with being under treatment for both infections can also result in social alienation and discrimination, further increasing the risk of voluntary withdrawal from treatment [6, 14]. Additional care for patients living with HIV, including psychosocial support, is thus essential to ensuring that side effects are effectively managed and treatment adherence is optimal [15].

Patients with RR-/MDR-TB in this study were more likely to achieve treatment success in comparison to those with pre-XDR-TB and XDR-TB. This is consistent with global treatment success rates estimates of just 56% and 30% for MDR-TB and XDR-TB patients, respectively [12, 16]. As such, in cases where treatment failure results in additional acquired antibiotic resistance, a prolonged and more complicated retreatment may be required, further reducing the odds of a successful outcome. This is particularly problematic for patients with already substantial resistance as it limits future treatment options to less effective and potentially more toxic regimens. In addition, treatment failure may prolong the period in which the patient is infectious, thus potentially contributing to ongoing community transmission. Although acquired resistance was not specifically recorded in this study, 12% of patients reported considerable antibiotic resistance (pre-XDR-/XDR-TB) at diagnosis. Due to the poor treatment success rate and risks associated with higher levels of resistance, treatment of these patients represents a major clinical and public health challenge for Vietnam’s healthcare system.

Patients in this study who attained culture conversion by the end of 4 months were considerably more likely to ultimately achieve treatment success compared to those whose sputum remained positive after 4 months. This highlights both the utility of routine and accurate testing, and the importance of bacteriological conversion early in treatment. While conversion after 2 months of treatment has been suggested as a possible means for predicting treatment success [17], the results of this study are consistent with evidence showing conversion results after 4 months to be a more useful indicator [18]. While both sputum smear and culture results were acceptable predictors of treatment success, we found culture results to be a more suitable option when adjusted for all variables included in the multivariable model.

The reduced odds of treatment success for males, compared to females, was consistent with studies showing poorer treatment outcomes amongst male patients treated for MDR-TB [19, 20]. This may be a reflection of the higher risk of loss to follow-up among male patients, which in turn may be related to the financial burden associated with MDR-TB treatment. Catastrophic treatment-related costs borne by the household have been shown to be a significant barrier to treatment success [5, 21, 22]. In cases where male patients are the primary breadwinner, loss of employment or time away from work during treatment can result in considerable income reduction, placing financial strain on a patient’s household. This in turn may prompt voluntary withdrawal from treatment in order to return to work, resulting in poorer treatment adherence amongst male patients. As such, financial support through the social health insurance (SHI) program may be an important protective measure against poor treatment outcomes. Although not significantly associated with treatment success, our study showed that patients receiving subsidies through the public social health insurance scheme experienced lower rates of loss to follow-up (see Additional file 1: Table S3), which is consistent with research in other settings [5, 6, 23]. This highlights the importance of financial support for patients during treatment, especially if the patient is the primary breadwinner for their household. As such, this support should include not only direct costs (e.g., medication and transportation), but also indirect costs, such as loss of wages, to compensate for the reduction of household income. This could have an effect on the overall treatment success rate by offering an incentive with which to complete treatment, thus improving patient retention.

This study has a number of limitations. As we relied upon routinely collected programmatic data, a proportion of cases lacked complete data, particularly for comorbidities and treatment toxicity results. Furthermore, screening for treatment toxicity was not standardised. This may have resulted in ascertainment bias, and an underestimation of the frequency of adverse events and their effect upon treatment outcomes. In addition, missing outcome data may have resulted in either an underestimation or overestimation of the overall treatment success rate. Although the characteristics of patients for whom a treatment outcome was unavailable were otherwise similar to that of patients with a reported treatment outcome (see Additional file 1: Tables S6 and S7), it is unclear as to how many of these patients may have achieved treatment success. A lack of complete case data also presented challenges in performing the multivariable analysis. As multiple imputation was used to complete the dataset used in the multivariable analysis, a level of uncertainty with the parameter estimates was unavoidable. However, while 20 imputations are considered sufficient to produce accurate estimates [24], 50 imputations were used in this study to ensure accuracy and preserve statistical testing power. Finally, the findings of this study are applicable to patients treated with regimens that accorded with WHO guidelines at the time. These results are not generalisable to patients treated with newer all-oral regimens.

This study has important policy implications for the treatment of MDR-TB patients in Vietnam. We found that a high proportion (34.5%) of patients did not achieve a successful treatment outcome. This indicates a significant gap between treatment policy and implementation. Unfortunately, the proportion of patients with successful treatment was lower than for the first cohort of patients with MDR-TB treated in Vietnam a decade ago. This may reflect the challenges in scaling-up care for MDR-TB, as well as regional differences in care. Additional interventions are required to retain patients in care, improve reporting of treatment toxicity, and optimize management of co-morbidities. A comprehensive standardized evaluation of co-morbidities at the time of enrolment in treatment may enable clinicians to provide holistic medical care and improve treatment outcomes. In addition, this study provides evidence to expand access to subsidised treatment for all patients, suggesting that further research is required to assess the effects of social health insurance coverage upon loss to follow-up during treatment. Financial support provided through insurance may have an important role in minimizing loss to follow-up, and including as many facets of the treatment process as possible in this scheme may have a beneficial impact on overall treatment adherence.

As advanced drug resistance significantly reduces the odd of success treatment outcome, pre-XDR-/XDR-TB patients should be carefully monitored to ensure treatment adherence, particularly during outpatient care. Drug susceptibility testing for second-line antibiotics should be used to individualise treatment in order to ensure optimal treatment outcomes and avoid acquired antibiotic resistance.

## Conclusions

This study found that among patients with MDR-TB, males, people living with HIV, and those with more extensive antibiotic resistance at diagnosis are at greatest risk of an unsuccessful treatment outcome. Efforts to optimise the management of co-morbidities (such as HIV), ensure rapid bacteriological conversion, and provide financial support for patients promise to improve treatment outcomes and may contribute to a reduction in the risk of community transmission of MDR-TB.

## Supplementary Information

**Additional file 1.** Supplementary figures and tables.

**Additional file 2.** Case report form template used for patient data collection.

**Additional file 3.** Multiple imputation parameters and results.

## Data Availability

The datasets used and/or analyzed during the current study are available from the corresponding author on reasonable request.

## Abbreviations

aOR: Adjusted odds ratio
cOR: Crude (unadjusted) odds ratio
DST: Drug susceptibility testing
HIV: Human immunodeficiency virus
HREC: Human Research Ethics Committee
MDR-TB: Multidrug-resistant tuberculosis
NTP: National tuberculosis programme
PMDT: Programmatic management of drug-resistant tuberculosis
Pre-XDR-TB: Pre-extensively drug-resistant tuberculosis
RR-TB: Rifampicin-resistant tuberculosis
SHI: Social health insurance
TB: Tuberculosis
RR-TB: Rifampicin-resistant tuberculosis
WHO: World Health Organization
XDR-TB: Extensively drug-resistant tuberculosis
