# Supplementary material for: Predictors of treatment outcomes among patients with multidrug-resistant tuberculosis in Vietnam: a retrospective cohort study
Source: BMC Infect Dis. 2022 Jan 20;22:68. doi: 10.1186/s12879-021-06992-x (PMC8772201; doi:10.1186/s12879-021-06992-x)
Supplement: Supplementary file 1 — Additional file 1. Supplementary figures and tables. [file 12879_2021_6992_MOESM1_ESM.docx]

**Additional File 1**

- Figure S1. Variable attrition following univariate and multivariate analyses
- Table S1. Variable definitions
- Table S2. Treatment outcome definitions
- Table S3. Univariate analysis of variable association with treatment failure
- Table S4. Univariate analysis of variable association with loss to follow-up
- Table S5. Univariate analysis of variable association with death
- Table S6. Characteristics of patients for whom treatment outcome was not evaluated
- Table S7. Microbiological outcomes and side effects for patients for whom a treatment outcome was not evaluated


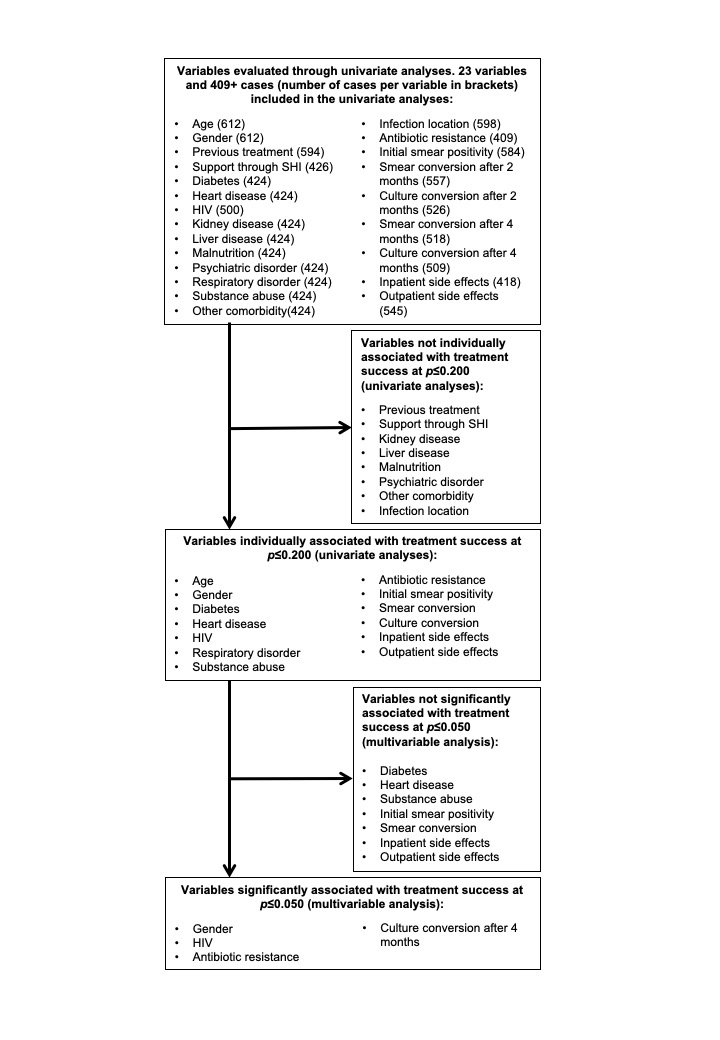


Figure S1. Variable attrition following univariate and multivariate analyses

| **Variable name** | **Variable description** *and options* | **% Missing data** |
| --- | --- | --- |
| Gender | *Male or female* | 0 |
| Age | Age, *in years*, as of the January 01 of the birth year | 0.2 |
| Previous treatment | Was the patient previously treated for any form of TB? *Yes or no* | 2.9 |
| Financially supported through SHI | Was the patient receiving financial assistance through social health insurance (SHI), as opposed to paying out of pocket? *Yes or no* | 30.8 |
| HIV status | Was the patient HIV positive at the commencement of treatment?  *Yes or no* | 20.4 |
| Other comorbidities | Did the patient have any pre-existing conditions at the commencement of treatment (possibilities: diabetes, heart disease, liver disease, kidney disease, malnutrition, psychiatric disorder, respiratory disorder, substance abuse, other comorbidity). *Yes or no* (for each)   - Respiratory disorder can refer to: atelectasis, bronchiectasis, bronchitis, bronchopneumonia, chronic pulmonary disease, lung tumor, pneumonia, pneumothorax, or respiratory failure - Substance abuse includes both drug or alcohol use | 30.8 each |
| Infection location | Where was the TB infection at the commencement of treatment? *Pulmonary, extrapulmonary, or both* | 2.6 |
| Antibiotic resistance | Was the patient classified as rifampicin-resistant (RR-TB), multidrug-resistant (MDR-TB), pre-extensively drug resistant (pre-XDR-TB), or extensively drug resistant (XDR-TB)? *RR/MDR-TB or Pre-XDR/XDR-TB*   - MDR-TB: resistance to rifampicin and isoniazid - Pre-XDR-TB: resistance to rifampicin, isoniazid, and either an injectable or fluoroquinolone - XDR-TB: resistance to rifampicin, isoniazid, a fluoquinolone, and at least one injectable | 33.4 |
| Sputum (smear) positivity | Did the initial sputum smear (month 0) have a positive result? *Yes or no*   - Smears were graded as either: negative, scanty, 1+, 2+, 3+, as per Word Health Organization and the International Union against Tuberculosis and Lung Disease (WHO-IUATLD) guidelines. Positivity included all results except negative. | 6.3 |
| Smear conversion after 2 months | Was the patient’s sputum smear at the end of the second month of treatment negative? *Yes or no*   - Smears were graded as either: negative, scanty, 1+, 2+, 3+, as per Word Health Organization and the International Union against Tuberculosis and Lung Disease (WHO-IUATLD) guidelines. Only a negative result satisfied an answer of yes. | 11.5 |
| Culture conversion after 2 months | Was the patient’s sputum culture result at the end of the second month of treatment negative? *Yes or no*   - Culture results were classified as: negative, 1+, 2+, 3+, as per Word Health Organization and the International Union against Tuberculosis and Lung Disease (WHO-IUATLD) guidelines. Only a negative result satisfied an answer of yes. | 16.5 |
| Side effect experienced – inpatient | Were any of the following side effects experienced while under inpatient treatment? *Yes or no*   - Nausea, vertigo, hearing loss, vision loss, hepatotoxicity, nephrotoxicity, neurotoxicity, or other side effect | 31.7 |
| Side effect experienced – outpatient | Were any of the following side effects experienced while under outpatient treatment? *Yes or no*   - Nausea, vertigo, hearing loss, vision loss, hepatotoxicity, nephrotoxicity, neurotoxicity, or other side effect | 13.6 |

Table S1: Variable definitions

Table S2: Treatment outcome definitions

| **Study Treatment**  **Outcome** | **Vietnamese PMDT Definitions**^a^ |
| --- | --- |
| Success | *Cured* - Patient has completed treatment according to the programme protocol and has at least 5 consecutive negative cultures from samples collected at least 30 days apart in the final 12 months of treatment. If only one positive culture is reported during that time, and there is no concomitant clinical evidence of deterioration, a patient may still be considered cured, provided that this positive culture is followed by a minimum of three consecutive negative cultures taken at least 30 days apart  OR  *Treatment completed* - Patient who has completed treatment according to the programme protocol but does not meet the definition for cure because of lack of bacteriological results (i.e., <5 cultures were performed in the final 12 months of treatment) |
| Failure | *Treatment failed* - Patient failed if two or more of the five cultures recorded in the final 12 months of therapy are positive, or if any one of the final three cultures is positive. (Treatment will also be considered to have failed if a clinical decision has been made to terminate treatment early due to poor clinical or radiological response or adverse events) |
| Loss to follow-up | *Loss to follow-up* - Treatment was interrupted for ≥2 consecutive months for any reason without medical approval (also referred to as ‘Defaulted’) |
| Death | *Died* - Patient died for any reason during MDR-TB treatment |
| Not evaluated | *Transferred out* - Patient was transferred to another reporting and recording unit and for whom the treatment outcome is unknown |

^a^ Vietnam National Tuberculosis Control Programme. Guidelines for the management and treatment of MDR-TB. Hanoi: Vietnam NTP; 2009.

Table S3: Univariate analysis of variable association with loss to follow-up (versus success, failure, or death)

| **Variable (number of cases reporting data)** | **Treatment outcome [n (%)]** | | **Odds of loss to follow-up versus success, failure, or death**  [cOR (95% CI)] | ***p*-value** |
| --- | --- | --- | --- | --- |
|  | **Loss to follow-up** | **Success, failure, or death** |  |  |
| Age (n=612)  ***Gender (n=612)***  ***Female***  ***Male***  Previous treatment (n=594)  *No*  *Yes*  ***Financially supported through SHI (n=426)***  ***No***  ***Yes***  ***Diabetes (n=424)***  ***No***  ***Yes***  ***Heart disease (n=424)***  ***No***  ***Yes***  ***HIV (n=500)***  ***No***  ***Yes***  Kidney disease (n=424)  *No*  *Yes*  ***Liver disease (n=424)***  ***No***  ***Yes***  ***Malnutrition (n=424)***  ***No***  ***Yes***  Psychiatric disorder (n=424)  *No*  *Yes*  ***Respiratory disorder***^b^ ***(n=424)***  ***No***  ***Yes***  Substance abuse (n=424)  *No*  *Yes*  Other comorbidity^c^ (n=424)  *No*  *Yes*  Infection location (n=598)  *Pulmonary*  *Extrapulmonary*  *Both*  ***Antibiotic resistance (n=409)***  ***RR/MDR-TB***^d^  ***Pre-XDR/XDR-TB***^e^  Initial sputum (smear) positivity (n=584)  *No*  *Yes*  ***Smear conversion after 2 months (n=557)***  ***No***  ***Yes***  Culture conversion after 2 months (n=526)  *No*  *Yes*  ***Smear conversion after 4 months (n=518)***  ***No***  ***Yes***  ***Culture conversion after 4 months (n=509)***  ***No***  ***Yes***  At least one side effect experienced during inpatient treatment (n=418)  *No*  *Yes*  At least one side effect experienced during outpatient treatment (n=545)  *No*  *Yes* | n/a^a^  ***16 (10.8%)***  ***91 (19.6%)***  19 (18.3%)  86 (17.6%)  ***26 (22.6%)***  ***43 (13.8%)***  ***58 (15.5%)***  ***12 (23.5%)***  ***63 (15.5%)***  ***7 (38.9%)***  ***71 (15.3%)***  ***13 (36.1%)***  69 (16.4%)  1 (50.0%)  ***63 (15.6%)***  ***7 (35.0%)***  ***65 (15.8%)***  ***5 (38.5%)***  69 (16.7%)  1 (8.3%)  ***56 (15.1%)***  ***14 (26.9%)***  66 (16.2%)  4 (23.5%)  67 (16.3%)  3 (23.1%)  98 (17.0%)  2 (16.7%)  2 (25.0%)  ***60 (16.8%)***  ***4 (7.7%)***  34 (15.9%)  65 (17.6%)  ***16 (20.8%)***  ***68 (14.2%)***  21 (17.5%)  57 (14.0%)  ***5 (31.3%)***  ***69 (13.7%)***  ***8 (23.5%)***  ***63 (13.3%)***  66 (17.1%)  6 (19.4%)  71 (16.0%)  18 (17.8%) | n/a^a^  ***132 (89.2%)***  ***373 (80.4%)***  85 (81.7%)  404 (82.4%)  ***89 (77.4%)***  ***268 (86.2%)***  ***315 (84.5%)***  ***39 (76.5%)***  ***343 (84.5%)***  ***11 (61.1%)***  ***393 (74.7%)***  ***23 (63.9%)***  353 (83.6%)  1 (50.0%)  ***341 (84.4%)***  ***13 (65.0%)***  ***346 (84.2%)***  ***8 (61.5%)***  343 (83.3%)  11 (91.7%)  ***316 (84.9%)***  ***38 (73.1%)***  341 (83.8%)  13 (76.5%)  344 (83.7%)  10 (76.9%)  480 (83.0%)  10 (83.3%)  6 (75.0%)  ***297 (83.2%)***  ***48 (92.3%)***  180 (84.1%)  305 (82.4%)  ***61 (79.2%)***  ***412 (85.8%)***  99 (82.5%)  349 (86.0%)  ***11 (68.7%)***  ***433 (86.3%)***  ***26 (76.5%)***  ***412 (86.7%)***  321 (82.9%)  25 (80.6%)  373 (84.0%)  83 (82.2%) | 1.01 (0.99 – 1.02)  ***1.00 (reference)***  ***2.01 (1.14 – 3.55)***  1.00 (reference)  0.95 (0.55 – 1.65)  ***1.00 (reference)***  ***0.55 (0.32 – 0.95)***  ***1.00 (reference)***  ***1.67 (0.83 – 3.38)***  ***1.00 (reference)***  ***3.47 (1.29 – 9.28)***  ***1.00 (reference)***  ***3.13 (1.51 – 6.46)***  1.00 (reference)  5.12 (0.32 – 82.78)  ***1.00 (reference)***  ***2.92 (1.12 – 7.59)***  ***1.00 (reference)***  ***3.33 (1.06 – 10.49)***  1.00 (reference)  0.45 (0.06 – 3.56)  ***1.00 (reference)***  ***2.08 (1.06 – 4.09)***  1.00 (reference)  1.59 (0.50 – 5.03)  1.00 (reference)  1.54 (0.41 – 5.75)  1.00 (reference)  0.98 (0.21 – 4.54)  1.63 (0.33 – 8.21)  ***1.00 (reference)***  ***0.41 (0.14 – 1.19)***  1.00 (reference)  1.13 (0.72 – 1.78)  ***1.00 (reference)***  ***0.63 (0.34 – 1.16)***  1.00 (reference)  0.77 (0.45 – 1.33)  ***1.00 (reference)***  ***0.35 (0.12 – 1.04)***  ***1.00 (reference)***  ***0.50 (0.22 – 1.15)***  1.00 (reference)  1.17 (0.46 – 2.96)  1.00 (reference)  1.14 (0.65 – 2.01) | 0.231  ***0.016***  0.862  ***0.030***  ***0.153***  ***0.013***  ***0.002***  0.250  ***0.029***  ***0.040***  0.451  ***0.034***  0.430  0.520  0.837  0.979  0.552  ***0.101***  0.602  ***0.135***  0.350  ***0.059***  ***0.101***  0.744  0.653 |

^a^ Age is represented as a continuous variable

^b^ Respiratory disorder refers to either: atelectasis, bronchiectasis, bronchitis, bronchopneumonia, chronic pulmonary disease, lung tumor, pneumonia, pneumothorax, or respiratory failure

^c^ Other comorbidity refers to either: adrenal failure, anemia, esophageal cancer, gout, pleural effusion, or seizure

^d^ Rifampicin-resistant or multidrug-resistant tuberculosis

^e^ Pre-extensively-resistant or extensively-resistant tuberculosis

Table S4: Univariate analysis of variable association with death (versus success, failure, or loss to follow-up)

| **Variable (number of cases reporting data)** | **Treatment outcome [n (%)]** | | **Odds of death versus success, failure, or death**  [cOR (95% CI)] | ***p*-value** |
| --- | --- | --- | --- | --- |
|  | **Death** | **Success, failure, or loss to follow-up** |  |  |
| ***Age (n=612)***  ***Gender (n=612)***  ***Female***  ***Male***  Previous treatment (n=594)  *No*  *Yes*  ***Financially supported through SHI (n=426)***  ***No***  ***Yes***  ***Diabetes (n=424)***  ***No***  ***Yes***  Heart disease (n=424)  *No*  *Yes*  ***HIV (n=500)***  ***No***  ***Yes***  Kidney disease (n=424)  *No*  *Yes*  Liver disease (n=424)  *No*  *Yes*  Malnutrition (n=424)  *No*  *Yes*  Psychiatric disorder (n=424)  *No*  *Yes*  ***Respiratory disorder***^c^ ***(n=424)***  ***No***  ***Yes***  ***Substance abuse (n=424)***  ***No***  ***Yes***  Other comorbidity^d^ (n=424)  *No*  *Yes*  Infection location (n=598)  *Pulmonary*  *Extrapulmonary*  *Both*  Antibiotic resistance (n=409)  *RR/MDR-TB*^e^  *Pre-XDR/XDR-TB*^f^  ***Initial sputum (smear) positivity (n=584)***  ***No***  ***Yes***  ***Smear conversion after 2 months (n=557)***  ***No***  ***Yes***  ***Culture conversion after 2 months (n=526)***  ***No***  ***Yes***  ***Smear conversion after 4 months (n=518)***  ***No***  ***Yes***  ***Culture conversion after 4 months (n=509)***  ***No***  ***Yes***  ***At least one side effect experienced during inpatient treatment (n=418)***  ***No***  ***Yes***  ***At least one side effect experienced during outpatient treatment (n=545)***  ***No***  ***Yes*** | ***n/a***^a^  ***8 (5.4%)***  ***42 (9.1%)***  8 (7.7%)  38 (7.8%)  ***5 (4.3%)***  ***31 (10.0%)***  ***26 (7.0%)***  ***7 (13.7%)***  32 (7.9%)  1 (5.6%)  ***31 (6.7%)***  ***5 (13.9%)***  33 (7.8%)  0  32 (7.9%)  1 (5.0%)  32 (7.8%)  1 (7.7%)  33 (8.0%)  0  ***23 (6.2%)***  ***10 (19.2%)***  ***29 (7.1%)***  ***4 (23.5%)***  32 (7.8%)  1 (7.7%)  49 (8.5%)  0  1 (12.5%)  27 (7.6%)  6 (11.5%)  ***10 (4.7%)***  ***36 (9.7%)***  ***10 (13.0%)***  ***29 (6.0%)***  ***14 (11.7%)***  ***21 (5.2%)***  ***3 (18.8%)***  ***35 (7.0%)***  ***5 (14.7%)***  ***31 (6.5%)***  ***28 (7.2%)***  ***5 (16.1%)***  ***36 (8.1%)***  ***4 (4.0%)*** | ***n/a***^a^  ***140 (94.6%)***  ***422 (90.9%)***  96 (92.3%)  452 (92.2%)  ***110 (95.7%)***  ***280 (90.0%)***  ***347 (93.0%)***  ***44 (86.3%)***  374 (92.1%)  17 (94.4%)  ***433 (93.3%)***  ***31 (86.1%)***  389 (92.2%)  2 (100%)  372 (92.1%)  19 (95.0%)  379 (92.2%)  12 (92.3%)  379 (92.0%)  12 (100%)  ***349 (93.8%)***  ***42 (80.8%)***  ***378 (92.9%)***  ***13 (76.5%)***  379 (92.2%)  12 (92.3%)  529 (91.5%)  12 (100%)  7 (87.5%)  330 (92.4%)  46 (88.5%)  ***204 (95.3%)***  ***334 (90.3%)***  ***67 (87.0%)***  ***451 (94.0%)***  ***106 (88.3%)***  ***385 (94.8%)***  ***13 (81.2%)***  ***467 (93.0%)***  ***29 (85.3%)***  ***444 (93.5%)***  ***359 (92.8%)***  ***26 (83.9%)***  ***408 (91.9%)***  ***97 (96.0%)*** | ***1.04 (1.02 – 1.06)***  ***1.00 (reference)***  ***1.74 (0.80 – 3.80)***  1.00 (reference)  1.01 (0.46 – 2.23)  ***1.00 (reference)***  ***2.44 (0.92 – 6.43)***  ***1.00 (reference)***  ***2.12 (0.87 – 5.18)***  1.00 (reference)  0.69 (0.09 – 5.33)  ***1.00 (reference)***  ***2.25 (0.82 – 6.20)***  n/a^b^  1.00 (reference)  0.61 (0.08 – 4.72)  1.00 (reference)  0.99 (0.12 – 7.84)  n/a^b^  ***1.00 (reference)***  ***3.61 (1.61 – 8.11)***  ***1.00 (reference)***  ***4.01 (1.23 – 13.09)***  1.00 (reference)  0.99 (0.12 – 7.84)  1.00 (reference)  n/a^2^  1.54 (0.19 – 12.79)  1.00 (reference)  1.59 (0.63 – 4.07)  ***1.00 (reference)***  ***2.20 (1.07 – 4.53)***  ***1.00 (reference)***  ***0.43 (0.20 – 0.92)***  ***1.00 (reference)***  ***0.41 (0.20 – 0.84)***  ***1.00 (reference)***  ***0.33 (0.09 – 1.19)***  ***1.00 (reference)***  ***0.41 (0.15 – 1.12)***  ***1.00 (reference)***  ***2.47 (0.88 – 6.92)***  ***1.00 (reference)***  ***0.47 (0.16 – 1.34)*** | ***0.000***  ***0.163***  0.983  ***0.072***  ***0.098***  0.720  ***0.116***  0.637  0.990  ***0.002***  ***0.021***  0.990  0.923  0.688  0.329  ***0.032***  ***0.031***  ***0.015***  ***0.090***  ***0.081***  ***0.086***  ***0.158*** |

^a^ Age is represented as a continuous variable

^b^ Insufficient cases with which to calculate an odds ratio

^c^ Respiratory disorder refers to either: atelectasis, bronchiectasis, bronchitis, bronchopneumonia, chronic pulmonary disease, lung tumor, pneumonia, pneumothorax, or respiratory failure

^d^ Other comorbidity refers to either: adrenal failure, anemia, esophageal cancer, gout, pleural effusion, or seizure

^e^ Rifampicin-resistant or multidrug-resistant tuberculosis

^f^ Pre-extensively-resistant or extensively-resistant tuberculosis

Table S5: Univariate analysis of variable association with treatment failure (versus success, loss to follow-up, or death)

| **Variable (number of cases reporting data)** | **Treatment outcome [n (%)]** | | **Odds of treatment failure versus success, loss to follow-up or death**  [cOR (95% CI)] | ***p*-value** |
| --- | --- | --- | --- | --- |
|  | **Treatment failure** | **Success, loss to follow-up, or death** |  |  |
| Age (n=612)  Gender (n=612)  *Female*  *Male*  Previous treatment (n=594)  *No*  *Yes*  Financially supported through SHI (n=426)  *No*  *Yes*  Diabetes (n=424)  *No*  *Yes*  Heart disease (n=424)  *No*  *Yes*  HIV (n=500)  *No*  *Yes*  Kidney disease (n=424)  *No*  *Yes*  Liver disease (n=424)  *No*  *Yes*  Malnutrition (n=424)  *No*  *Yes*  Psychiatric disorder (n=424)  *No*  *Yes*  Respiratory disorder^3^ (n=424)  *No*  *Yes*  Substance abuse (n=424)  *No*  *Yes*  Other comorbidity^4^ (n=424)  *No*  *Yes*  Infection location (n=598)  *Pulmonary*  *Extrapulmonary*  *Both*  ***Antibiotic resistance (n=409)***  ***RR/MDR-TB^5^***  ***Pre-XDR/XDR-TB^6^***  Initial sputum (smear) positivity (n=584)  *No*  *Yes*  Smear conversion after 2 months (n=557)  *No*  *Yes*  Culture conversion after 2 months (n=526)  *No*  *Yes*  Smear conversion after 4 months (n=518)  *No*  *Yes*  ***Culture conversion after 4 months (n=509)***  ***No***  ***Yes***  At least one side effect experienced during inpatient treatment (n=418)  *No*  *Yes*  ***At least one side effect experienced during outpatient treatment (n=545)***  ***No***  ***Yes*** | n/a^1^  12 (8.1%)  42 (9.1%)  12 (11.5%)  41 (8.4%)  12 (10.4%)  26 (8.4%)  33 (8.8%)  4 (7.8%)  36 (8.9%)  1 (5.6%)  43 (9.3%)  3 (8.3%)  37 (8.8%)  0  36 (8.9%)  1 (5.0%)  37 (9.0%)  0  36 (8.7%)  1 (8.3%)  37 (9.9%)  0  35 (8.6%)  2 (11.8%)  35 (8.5%)  2 (15.4%)  53 (9.2%)  1 (8.3%)  0  ***15 (4.2%)***  ***18 (34.6%)***  21 (9.8%)  33 (8.9%)  10 (13.0%)  42 (8.8%)  10 (8.3%)  39 (9.6%)  2 (12.5%)  34 (6.8%)  ***6 (17.6%)***  ***30 (6.3%)***  32 (8.3%)  4 (12.9%)  ***43 (9.7%)***  ***4 (4.0%)*** | n/a^1^  136 (91.9%)  422 (90.9%)  92 (88.5%)  449 (91.6%)  103 (89.6%)  285 (%91.6)  340 (91.2%)  47 (92.2%)  370 (91.1%)  17 (94.4%)  421 (90.7%)  33 (91.7%)  385 (91.2%)  2 (100%)  368 (91.1%)  19 (95.0%)  374 (91.0%)  13 (100%)  376 (91.3%)  11 (91.7%)  335 (90.1%)  52 (100%)  372 (91.4%)  15 (88.2%)  376 (91.5%)  11 (84.6%)  525 (90.8%)  11 (91.7%)  8 (100%)  ***342 (95.8%)***  ***34 (65.4%)***  193 (90.2%)  337 (91.1%)  67 (87.0%)  438 (91.2%)  110 (91.7%)  367 (90.4%)  14 (87.5%)  468 (93.2%)  ***28 (82.4%)***  ***445 (93.7%)***  355 (91.7%)  27 (87.1%)  ***401 (90.3%)***  ***97 (96.0%)*** | 0.99 (0.97 – 1.01)  1.00 (reference)  1.13 (0.58 – 2.20)  1.00 (reference)  0.70 (0.35 – 1.38)  1.00 (reference)  0.78 (0.38 – 1.61)  1.00 (reference)  0.88 (0.30 – 2.59)  1.00 (reference)  0.61 (0.08 – 4.68)  1.00 (reference)  0.89 (0.26 – 3.02)  n/a^2^  1.00 (reference)  0.54 (0.07 – 4.14)  n/a^2^  1.00 (reference)  0.95 (0.12 – 7.57)  n/a^2^  1.00 (reference)  1.42 (0.31 – 6.45)  1.00 (reference)  1.95 (0.42 – 9.17)  1.00 (reference)  0.90 (0.11 – 7.11)  n/a^2^  ***1.00 (reference)***  ***12.07 (5.59 – 26.08)***  1.00 (reference)  0.90 (0.51 – 1.60)  1.00 (reference)  0.64 (0.31 – 1.34)  1.00 (reference)  1.17 (0.57 – 2.42)  1.00 (reference)  0.51 (0.11 – 2.33)  ***1.00 (reference)***  ***0.32 (0.12 – 0.82)***  1.00 (reference)  1.64 (0.54 – 4.99)  ***1.00 (reference)***  ***0.39 (0.14 – 1.10)*** | 0.219  0.725  0.305  0.506  0.812  0.630  0.852  0.551  0.961  0.652  0.396  0.995  0.921  ***0.000***  0.719  0.239  0.674  0.384  ***0.018***  0.381  ***0.074*** |

^a^ Age is represented as a continuous variable

^b^ Insufficient cases with which to calculate an odds ratio

^c^ Respiratory disorder refers to either: atelectasis, bronchiectasis, bronchitis, bronchopneumonia, chronic pulmonary disease, lung tumor, pneumonia, pneumothorax, or respiratory failure

^d^ Other comorbidity refers to either: adrenal failure, anemia, esophageal cancer, gout, pleural effusion, or seizure

^e^ Rifampicin-resistant or multidrug-resistant tuberculosis

^f^ Pre-extensively-resistant or extensively-resistant tuberculosis

Table S6. Characteristics of patients for whom treatment outcome was not evaluated

|  | **Patients for whom a treatment outcome was ‘not evaluated’** | | | **Patients with an evaluated treatment outcome – success, failure, loss to follow-up, or death (both provinces)** |
| --- | --- | --- | --- | --- |
|  | **Hanoi Province** | **Thanh Hoa Province** | **Both Provinces** |  |
| **Total** | **24** | **26** | **50** | **612** |
| **Gender**  *Male*  *Female* | **n = 24**  19 (79.2%)  5 (20.8%) | **n = 26**  23 (88.5%)  3 (11.5%) | **n = 50**  42 (84.0%)  8 (16.0%) | **n = 612**  464 (76.4%)  148 (23.6%) |
| **Age (years)**  *Mean (IQR)*  *By group (years)*  *≤19*  *20-39*  *40-59*  *60-79*  *≥80*  *Not reported* | **n = 24**  44.0 (31.3-60.0)  0 (0.0%)  10 (41.7%)  8 (33.3%)  6 (25.0%)  0 (0.0%)  0 | **n = 25**  38.0 (32.0-48.5)  0 (0.0%)  13 (52.0%)  10 (40.0%)  2 (8.0%)  0 (1.0%)  1 | **n = 49**  40.0 (31.5-51.5)  0 (0.0%)  23 (46.9%)  18 (36.7%)  8 (16.4%)  0 (0.0%)  1 | **n = 612**  43.0 (32.0-55.0)  13 (2.0%)  245 (40.5%)  260 (42.1%)  90 (14.8%)  4 (0.6%)  0 |
| **Previous TB treatment**  *Yes*  *No*  *Not reported* | **n = 24**  20 (83.3%)  4 (16.7%)  0 | **n = 25**  22 (88.0%)  3 (12.0%)  0 | **n = 49**  42 (85.7%)  7 (14.3%)  0 | **n = 612**  490 (80.1%)  104 (19.9%)  18 |
| **Financially supported through social health insurance**  *Yes*  *No*  *Not reported* | **n = 15**  11 (73.3%)  4 (26.7%)  9 | **n = 17**  11 (64.7%)  6 (35.3%)  8 | **n = 32**  22 (68.8%)  10 (31.4%)  17 | **n = 426**  311 (73.0%)  115 (27.0%)  186 |
| **HIV status**  *Positive*  *Negative*  *Not reported* | **n = 19**  1 (5.2%)  18 (94.8%)  5 | **n = 8**  0 (0.0%)  8 (100.0%)  18 | **n = 27**  1 (3.7%)  26 (96.3%)  23 | **n = 500**  36 (7.2%)  464 (92.8%)  112 |
| **Other comorbidities**  *Diabetes*  *Heart disease*  *Kidney disease*  *Liver disease*  *Malnutrition*  *Psychiatric disorder*  *Respiratory disorder*^a^  *Substance abuse*  *Other*^b^  *Not reported* | **n = 15**  1 (6.7%)  0 (0.0%)  0 (0.0%)  0 (0.0%)  0 (0.0%)  1 (6.7%)  0 (0.0%)  0 (0.0%)  0 (0.0%)  9 | **n = 19**  0 (0.0%)  0 (0.0%)  0 (0.0%)  0 (0.0%)  1 (5.3%)  0 (0.0%)  2 (10.6%)  1 (5.3%)  0 (0.0%)  7 | **n = 34**  1 (2.9%)  0 (0.0%)  0 (0.0%)  0 (0.0%)  1 (2.9%)  1 (2.9%)  2 (5.8%)  1 (2.9%)  0 (0.0%)  16 | **n = 424**  51 (12.0%)  18 (4.2%)  2 (0.5%)  20 (4.7%)  13 (3.1%)  12 (2.8%)  52 (12.3%)  17 (4.0%)  13 (3.1%)  188 |
| **Infection site**  *Pulmonary*  *Extra-pulmonary*  *Both*  *Not reported* | **n = 24**  21 (87.5%)  0 (0.0%)  3 (12.5%)  0 | **n =23**  23 (100.0%)  0 (0.0%)  0 (0.0%)  3 | **n = 47**  44 (93.6%)  0 (0.0%)  3 (6.4%)  3 | **n = 598**  578 (96.7%)  12 (2.0%)  8 (1.3%)  14 |
| **Initial sputum smear grade**  *Negative*  *Scanty*  *1+*  *2+*  *3+*  *Not reported* | **n = 17**  1 (5.9%)  0 (0.0%)  13 (76.5%)  0 (0.0%)  3 (17.6%)  7 | **n = 19**  9 (47.4%)  1 (5.3%)  5 (26.3%)  3 (15.7%)  1 (5.3%)  7 | **n = 36**  10 (27.8%)  1 (2.8%)  18 (50.0%)  3 (8.3%)  4 (11.1%)  14 | **n = 584**  218 (37.3%)  13 (2.2%)  190 (32.5%)  77 (13.2%)  86 (20.3%)  28 |
| **Antibiotic resistance at enrolment**  *RR-/MDR-TB only*  *Pre-XDR-TB*  *XDR-TB*  *Not reported* | **n = 13**  12 (92.3%)  0 (0.0%)  1 (7.6%)  11 | **n = 19**  19 (100.0%)  0 (0.0%)  0 (0.0%)  7 | **n = 32**  31 (96.9%)  0 (0.0%)  1 (3.1%)  18 | **n = 409**  357 (87.3%)  36 (8.8%)  16 (3.9%)  203 |

Notes: **bolded numbers** indicate the number of individuals for whom data were available (percentages exclude those for whom data were not reported).

^a^ Respiratory disorder refers to either: atelectasis, bronchiectasis, bronchitis, bronchopneumonia, chronic pulmonary disease, lung tumor, pneumonia, pneumothorax, or respiratory failure

^b^ Other comorbidity refers to either: adrenal failure, anemia, esophageal cancer, gout, pleural effusion, or seizure

Table S7. Microbiological outcomes and side effects for patients for whom a treatment outcome was not evaluated

|  | **Patients for whom a treatment outcome was ‘not evaluated’** | | | **Patients with an evaluated treatment outcome – success, failure, loss to follow-up, or death (both provinces)** |
| --- | --- | --- | --- | --- |
|  | **Hanoi Province** | **Thanh Hoa Province** | **Both Provinces** |  |
| **Total** | **n = 24** | **n = 26** | **n = 50** | **n = 612** |
| **Treatment duration [median (IQR)]**  *Start to completion (months)*  *Inpatient regimen (days)* | 1.1 (1.0-13.6)  26 (13-35) | 20.0 (9.7-21.3)  10 (6-12) | 18.3 (2.6-33.3)  13 (10-28) | 19.0 (12.0-20.0)  21 (15-30) |
| **Smear conversion after 2 months**  *Yes*  *No*  *Not reported*  **Smear conversion after 4 months**  *Yes*  *No*  *Not reported*  **Culture conversion after 2 months**  *Yes*  *No*  *Not reported*  **Culture conversion after 4 months**  *Yes*  *No*  *Not reported* | **n = 11**  9 (81.8%)  2 (18.2%)  13  **n = 8**  7 (87.5%)  1 (12.5%)  16  **n = 11**  9 (81.8%)  2 (18.2%)  13  **n = 8**  7 (87.5%)  1 (12.5%)  16 | **n = 18**  14 (77.8%)  4 (22.2%)  8  **n = 11**  10 (90.9%)  1 (9.1%)  15  **n = 16**  0 (0.0%)  16 (100.0%)  10  **n = 11**  6 (54.5%)  5 (45.5%)  15 | **n = 29**  23 (79.3%)  6 (20.7%)  21  **n = 19**  17 (89.5%)  2 (10.5%)  31  **n = 27**  9 (33.3%)  18 (66.7%)  23  **n = 19**  13 (68.4%)  6 (31.6%)  31 | **n = 557**  480 (86.2%)  77 (13.8%)  55  **n = 518**  502 (96.9%)  16 (3.1%)  94  **n = 526**  406 (77.2%)  120 (22.8%)  86  **n = 509**  475 (93.3%)  34 (6.7%)  103 |
| **Side effects during treatment**  ***At least one inpatient side effect reported***  *Yes*  *No*  *Not reported*  ***At least one outpatient side effect reported***  *Yes*  *No*  *Not reported* | **n = 15**  4 (26.7%)  11 (73.3%)  9  **n = 16**  1 (6.3%)  15 (93.7%)  8 | **n = 19**  4 (21.1%)  15 (78.9%)  7  **n = 11**  5 (45.5%)  6 (54.5%)  15 | **n = 34**  8 (23.5%)  26 (76.5%)  16  **n = 27**  6 (22.2%)  21 (77.8%)  23 | **n = 418**  31 (7.4%)  387 (92.6%)  194  **n = 545**  101 (18.5%)  444 (81.5%)  67 |
| **Outpatient side effects reported^a^**  *Blood disorder*  *Bowel pain*  *Hearing loss*  *Hepatotoxicity*  *High blood sugar*  *High uric acid*  *Hypokalemia*  *Joint pain*  *Loss of appetite*  *Nephrotoxicity*  *Neurotoxicity*  *Nausea*  *Vision loss*  *Vertigo*  *Not reported* | **n = 3**  0 (0%)  0 (0%)  0 (0%)  0 (0%)  0 (0%)  0 (0%)  0 (0%)  1 (33.3%)  1 (33.3%)  0 (0%)  0 (0%)  0 (0%)  0 (0%)  1 (33.3%)  21 | **n = 6**  0 (0%)  0 (0%)  0 (0%)  0 (0%)  0 (0%)  0 (0%)  0 (0%)  3 (27.3%)  0 (0%)  0 (0%)  0 (0%)  2 (18.2%)  0 (0%)  1 (9.1%)  20 | **n = 9**  0 (0%)  0 (0%)  0 (0%)  0 (0%)  0 (0%)  0 (0%)  0 (0%)  4 (14.8%)  1 (3.7%)  0 (0%)  0 (0%)  2 (7.4%)  0 (0%)  2 (7.4%)  41 | **n = 238**  6 (2.5%)  5 (2.0%)  12 (6.0%)  35 (14.7%)  10 (4.2%)  42 (17.6%)  15 (6.3%)  31 (13.0%)  10 (4.2%)  17 (7.1%)  8 (3.4%)  21 (8.8%)  9 (3.8%)  17 (7.1%)  374 |

Notes: **bolded numbers** indicate the number of individuals for whom data were available (percentages exclude those for whom data were not reported).

^a^ Patients could report more than one side effect
