# Supplementary material for: Predictors of treatment outcomes among patients with multidrug-resistant tuberculosis in Vietnam: a retrospective cohort study
Source: BMC Infect Dis. 2022 Jan 20;22:68. doi: 10.1186/s12879-021-06992-x (PMC8772201; doi:10.1186/s12879-021-06992-x)
Supplement: Supplementary file 2 — Additional file 2. Case report form template used for patient data collection. [file 12879_2021_6992_MOESM2_ESM.docx]

**Additional File 2 - Case report form template**

**Người thu thập / *Staff name:________________________***

**Ngày thu thập / *Date completed*:____**/____/______

**THÔNG TIN BỆNH NHÂN / PATIENT DETAILS**

1. **STT PĐ-IV / *Patient ID (from registration book)*:_*______________________________________***
2. **Họ tên bệnh nhân / *Patient's name*:________________________________________________**
3. **Giới tính / *Gender***: Nam/*Male* [ ] Nữ/*Female* [ ] No data [ ]
4. **Ngày sinh / *Birthdate* (dd/mm/yyyy): _______**/_______ /_______
5. **Tuổi / *Age*: ____________________________________________________________________**
6. **Quốc tịch / *Nationality*:** Việt Nam [ ] Khác/*Other* [ ] No data [ ]
7. **Địa chỉ / *Address*:_______________________________________________________________**
8. **Số điện thoại / *Phone number*:____________________________________________________**
9. **Nghề nghiệp / *Job*:______________________________________________________________**
10. **Đối tượng / *Payment option*:**

BHYT*/Insurance* [ ] Tự trả*/Self-paid* [ ] Miễn*/Free* [ ] Khác/*Other* [ ] No data [ ]

Đối tượng khác, nếu có:__________________________________________________________

1. **Đến khám tại / *Notification health unit*:_____________________________________________**

**BỆNH KÈM THEO */ COMORBIDITIES***

1. **Tình trạng nhiễm HIV của bệnh nhân? / *HIV status*:**

HIV dương tính/*Positive* [ ] HIV âm tính/*Negative* [ ] Không rõ/*Unclear* [ ] No data [ ]

1. **Ngày bệnh nhân có kết quả xét nghiệm HIV? / *HIV diagnosis date*: _______**/_______ /_______

Nếu HIV dương tính, trả lời các câu từ 14 đến 18. Nếu không, bỏ qua.

1. **Bệnh nhân có được điều trị ARV trong thời gian điều trị lao? / *HIV treatment – ARV*:**

No [ ] Yes [ ] No data [ ]

1. **Ngày bệnh nhân bắt đầu điều trị ARV? / *ARV treatment date*: _______**/_______ /_______
2. **Bệnh nhân có được điều trị Cotrimoxazol trong thời gian điều trị lao? / *HIV treatment – CPT*:**

No [ ] Yes [ ] No data [ ]

1. **Ngày bệnh nhân bắt đầu điều trị Cotrimoxazol? / *CPT treatment date*:_____**/_____ /______

***Bệnh kèm theo tại thời điểm bắt đầu điều trị:***

1. **Bệnh nhân có mắc đái tháo đường? / Comorbidities – Diabetes**: No [ ] Yes [ ] No data [ ]
2. **Bệnh nhân có mắc bệnh tim? / *Comorbidities - Heart disease:*** No [ ] Yes [ ] No data [ ]
3. **Bệnh nhân có mắc COPD? / *Comorbidities – COPD*:** No [ ] Yes [ ] No data [ ]
4. **Bệnh nhân có mắc bệnh gan? / *Comorbidities - Liver disease*:** No [ ] Yes [ ] No data [ ]
5. **Bệnh nhân có mắc bệnh thận? / *Comorbidities - Kidney disease*:** No [ ] Yes [ ] No data [ ]
6. **Bệnh nhân có thiếu dinh dưỡng? /*Comorbidities - Malnutrition*:** No [ ] Yes [ ] No data [ ]
7. **Bệnh nhân có mắc bệnh nào khác? / *Comorbidities – Other*:**_____________________________

**THÔNG TIN VỀ ĐIỀU TRỊ */ TREATMENT DETAILS***

1. **Phân loại bệnh nhân khi đăng ký điều trị / *Patient treatment classification*:**

Mới/*New* [ ] Tái phát/*Relapse* [ ] Điều trị lại sau bỏ trị/*Retreatment* [ ]
Thất bại phác đồ I/*First failed regimen* [ ] Thất bại phác đồ II/*Second failed regimen* [ ]
Chuyển đến/*Transfer in* [ ] Khác / *Other* [ ] No data [ ]

Phân loại khác, nếu có:___________________________________________________________

1. **Vị trí tổn thương lao / *Infection location*:**

Tại phổi/*Pulmonary* [ ] Ngoài phổi/*Extrapulmonary* [ ] Cả hai/*Both* [ ] No data [ ]

1. **Ngày bệnh nhân đến khám / *Registration date*: ______**/______ /______
2. **Ngày thực hiện XN chẩn đoán MDR-TB/ *Diagnostic test date*: ______**/______ /______ (Tên XN:__________)
3. **Ngày có kết quả chẩn đoán MRD-TB / *Diagnosis date*: ______**/______ /______
4. **Ngày bắt đầu điều trị / *Start treatment date*: ______**/______ /______
5. **Ngày kết thúc điều trị / *Finish treatment date*: ______**/______ /______
6. **Kết quả điều trị / *Treatment outcome*:______________________________________________**

**______________________________________________________________________________**

1. **Tổng thời gian điều trị giai đoạn tấn công / *Total length of intensive phase*:_______________** (tháng)
2. **Tổng thời gian điều trị giai đoạn duy trì / *Total length of continuation phase*:______________** (tháng)
3. **Bệnh nhân có bệnh gì kèm theo tại thời điểm kết thúc điều trị? / *Comorbidities at completion of treatment*:_____________________________________________________________________________________________________________________________________________________________**

**PHÁC ĐỒ ĐIỀU TRỊ / *DRUG REGIMENS***

***NỘI TRÚ***

1. **Bệnh nhân sử dụng phác đồ lao nào khi điều trị nội trú? / *Inpatient drug regimen:*** ________________

**Các thuốc được sử dụng trong phác đồ điều trị nội trú? / *Inpatient drug regimen:***

H [ ] R [ ] E [ ] Z [ ] S [ ] Th [ ] Am [ ] Km [ ] Cm [ ] Cfx [ ] Ofx [ ] Mfx [ ] Gfx [ ] Pto [ ] Eto [ ] Cs [ ] PAS [ ] Fq [ ] Khác/*Other* [ ] No data [ ]

Thuốc khác, nếu có:__________________________________________________________

1. **Ngày bắt đầu điều trị nội trú bằng phác đồ lao / *Regimen start date*: _______**/_______ /_______
2. **Khoa điều trị nội trú / *Treatment facility*:____________________________________________**
3. **Bệnh nhân có kết quả kháng các loại thuốc nào trước điều trị nội trú? / *Type of TB drug resistance:***

H [ ] R [ ] E [ ] Z [ ] S [ ] Th [ ] Am [ ] Km [ ] Cm [ ] Cfx [ ] Ofx [ ] Mfx [ ] Gfx [ ] Pto [ ] Eto [ ] Cs [ ] PAS [ ] Fq [ ]

Khác/*Other* [ ] No data [ ]

Thuốc khác, nếu có:______________________________________________________________

1. **Các tác dụng phụ gặp phải trong khi điều trị nội trú / *Side effects*:**

| **Tác dụng phụ / *Side effects*** | **Xử trí */ Treatment for side effects*** |
| --- | --- |
| Buồn nôn*/Severe nausea* [ ]  Ngày:________________________ | Ngừng thuốc/Discontinued drug:______ [    ] Không xử trí/No change [    ] Ngừng điều trị/Discontinued treatment [    ]    No data [    ] |
| Chóng mặt/*Vertigo* [ ]  Ngày:________________________ | Ngừng thuốc/Discontinued drug:______ [    ] Không xử trí/No change [    ] Ngừng điều trị/Discontinued treatment [    ]    No data [    ] |
| Nghe kém*/Hearing loss* [ ]  Ngày:________________________ | Ngừng thuốc/Discontinued drug:______ [    ] Không xử trí/No change [    ] Ngừng điều trị/Discontinued treatment [    ]    No data [    ] |
| Nhìn mờ*/Vision loss* [ ]  Ngày:________________________ | Ngừng thuốc/Discontinued drug:______ [    ] Không xử trí/No change [    ] Ngừng điều trị/Discontinued treatment [    ]    No data [    ] |
| Độc gan*/Hepatotoxicity* [ ]  Ngày:________________________ | Ngừng thuốc/Discontinued drug:______ [    ] Không xử trí/No change [    ] Ngừng điều trị/Discontinued treatment [    ]    No data [    ] |
| Độc thận*/Nephrotoxicity* [ ]  Ngày:________________________ | Ngừng thuốc/Discontinued drug:______ [    ] Không xử trí/No change [    ] Ngừng điều trị/Discontinued treatment [    ]    No data [    ] |
| Độc thần kinh*/Neurotoxicity* [ ]  Ngày:________________________ | Ngừng thuốc/Discontinued drug:______ [    ] Không xử trí/No change [    ] Ngừng điều trị/Discontinued treatment [    ]    No data [    ] |
| Khác*/Other* [ ]  ____________________________ Ngày:________________________ | Ngừng thuốc/Discontinued drug:______ [    ] Không xử trí/No change [    ] Ngừng điều trị/Discontinued treatment [    ]    No data [    ] |
| No data [ ] | |

***NGOẠI TRÚ***

1. **Bệnh nhân sử dụng phác đồ lao nào khi điều trị ngoại trú? / *Outpatient drug regimen:*** _____________

**Các thuốc được sử dụng trong phác đồ điều trị ngoại trú? / *Outpatient drug regimen:***

H [ ] R [ ] E [ ] Z [ ] S [ ] Th [ ] Am [ ] Km [ ] Cm [ ] Cfx [ ] Ofx [ ] Mfx [ ] Gfx [ ] Pto [ ] Eto [ ] Cs [ ] PAS [ ] Fq [ ] Khác/*Other* [ ] No data [ ]

Thuốc khác, nếu có:______________________________________________________________

1. **Ngày bắt đầu điều trị ngoại trú / *Regimen start date*: _______**/_______ /_______
2. **Nơi điều trị ngoại trú / *Treatment facility*:___________________________________________**
3. **Bệnh nhân có kết quả kháng các loại thuốc nào trước điều trị ngoại trú? / *Type of TB drug resistance:***

H [ ] R [ ] E [ ] Z [ ] S [ ] Th [ ] Am [ ] Km [ ] Cm [ ] Cfx [ ] Ofx [ ] Mfx [ ] Gfx [ ] Pto [ ] Eto [ ] Cs [ ] PAS [ ] Fq [ ] Khác/*Other* [ ] No data [ ]

Thuốc khác, nếu có:______________________________________________________________

1. **Các tác dụng phụ gặp phải trong khi điều trị ngoại trú / *Side effects*:**

| **Tác dụng phụ / *Side effects*** | **Xử trí */ Treatment for side effects*** |
| --- | --- |
| Buồn nôn*/Severe nausea* [ ]  Ngày:________________________ | Ngừng thuốc/Discontinued drug:______ [    ] Không xử trí/No change [    ] Ngừng điều trị/Discontinued treatment [    ]    No data [    ] |
| Chóng mặt/*Vertigo* [ ]  Ngày:________________________ | Ngừng thuốc/Discontinued drug:______ [    ] Không xử trí/No change [    ] Ngừng điều trị/Discontinued treatment [    ]    No data [    ] |
| Nghe kém*/Hearing loss* [ ]  Ngày:________________________ | Ngừng thuốc/Discontinued drug:______ [    ] Không xử trí/No change [    ] Ngừng điều trị/Discontinued treatment [    ]    No data [    ] |
| Nhìn mờ*/Vision loss* [ ]  Ngày:________________________ | Ngừng thuốc/Discontinued drug:______ [    ] Không xử trí/No change [    ] Ngừng điều trị/Discontinued treatment [    ]    No data [    ] |
| Độc gan*/Hepatotoxicity* [ ]  Ngày:________________________ | Ngừng thuốc/Discontinued drug:______ [    ] Không xử trí/No change [    ] Ngừng điều trị/Discontinued treatment [    ]    No data [    ] |
| Độc thận*/Nephrotoxicity* [ ]  Ngày:________________________ | Ngừng thuốc/Discontinued drug:______ [    ] Không xử trí/No change [    ] Ngừng điều trị/Discontinued treatment [    ]    No data [    ] |
| Độc thần kinh*/Neurotoxicity* [ ]  Ngày:________________________ | Ngừng thuốc/Discontinued drug:______ [    ] Không xử trí/No change [    ] Ngừng điều trị/Discontinued treatment [    ]    No data [    ] |
| Khác*/Other* [ ]  ____________________________ Ngày:________________________ | Ngừng thuốc/Discontinued drug:______ [    ] Không xử trí/No change [    ] Ngừng điều trị/Discontinued treatment [    ]    No data [    ] |
| No data [ ] | |

***THAY ĐỔI PHÁC ĐỒ***

1. **Bệnh nhân có được đổi phác đồ nào khác trong quá trình điều trị? / *Drug regimen* change:** __________________________________________________________________________________

**Các thuốc được sử dụng trong phác đồ điều trị mới? / *Drug regimen* change*:***

H [ ] R [ ] E [ ] Z [ ] S [ ] Th [ ] Am [ ] Km [ ] Cm [ ] Cfx [ ] Ofx [ ] Mfx [ ] Gfx [ ] Pto [ ] Eto [ ] Cs [ ] PAS [ ] Fq [ ] Khác/*Other* [ ] No data [ ]

Thuốc khác, nếu có:_______________________________________________________________

1. **Lý do thay đổi phác đồ / *Reason for change*:________________________________________________________________________________________________________________________________________________________**
2. **Ngày bắt đầu thay đổi phác đồ / *Regimen start date*: _______**/_______ /_______
3. **Nơi bệnh nhân nhận thuốc mới / *Treatment facility*:____________________________________**
4. **Bệnh nhân có kết quả kháng các loại thuốc nào trước khi dùng phác đồ mới? / *Type of TB drug resistance*:**

H [ ] R [ ] E [ ] Z [ ] S [ ] Th [ ] Am [ ] Km [ ] Cm [ ] Cfx [ ] Ofx [ ] Mfx [ ] Gfx [ ] Pto [ ] Eto [ ] Cs [ ] PAS [ ] Fq [ ] Khác/*Other* [ ] No data [ ]

Thuốc khác, nếu có:_______________________________________________________________

1. **Các tác dụng phụ gặp phải khi sử dụng phác đồ mới / *Side effects*:**

| **Tác dụng phụ / *Side effects*** | **Xử trí */ Treatment for side effects*** |
| --- | --- |
| Buồn nôn*/Severe nausea* [ ]  Ngày:________________________ | Ngừng thuốc/Discontinued drug:______ [    ] Không xử trí/No change [    ] Ngừng điều trị/Discontinued treatment [    ]    No data [    ] |
| Chóng mặt/*Vertigo* [ ]  Ngày:________________________ | Ngừng thuốc/Discontinued drug:______ [    ] Không xử trí/No change [    ] Ngừng điều trị/Discontinued treatment [    ]    No data [    ] |
| Nghe kém*/Hearing loss* [ ]  Ngày:________________________ | Ngừng thuốc/Discontinued drug:______ [    ] Không xử trí/No change [    ] Ngừng điều trị/Discontinued treatment [    ]    No data [    ] |
| Nhìn mờ*/Vision loss* [ ]  Ngày:________________________ | Ngừng thuốc/Discontinued drug:______ [    ] Không xử trí/No change [    ] Ngừng điều trị/Discontinued treatment [    ]    No data [    ] |
| Độc gan*/Hepatotoxicity* [ ]  Ngày:________________________ | Ngừng thuốc/Discontinued drug:______ [    ] Không xử trí/No change [    ] Ngừng điều trị/Discontinued treatment [    ]    No data [    ] |
| Độc thận*/Nephrotoxicity* [ ]  Ngày:________________________ | Ngừng thuốc/Discontinued drug:______ [    ] Không xử trí/No change [    ] Ngừng điều trị/Discontinued treatment [    ]    No data [    ] |
| Độc thần kinh*/Neurotoxicity* [ ]  Ngày:________________________ | Ngừng thuốc/Discontinued drug:______ [    ] Không xử trí/No change [    ] Ngừng điều trị/Discontinued treatment [    ]    No data [    ] |
| Khác*/Other* [ ]  ____________________________ Ngày:________________________ | Ngừng thuốc/Discontinued drug:______ [    ] Không xử trí/No change [    ] Ngừng điều trị/Discontinued treatment [    ]    No data [    ] |
| No data [ ] | |

**GHI CHÚ / *NOTES***

| **Tên thuốc viết tắt / *Drug Key*** | | | | |
| --- | --- | --- | --- | --- |
| H - Isoniazid | R - Rifampicin | E - Ethambutol | Z - Pyrazinamide | S - Streptomycin |
| Th - Thioacetazone | Am - Amikacin | Km - Kanamycin | Cm - Capreomycin | Cfx - Ciprofloxacin |
| Ofx - Ofloxacin | Mfx - Moxifloxacin | Gfx - Gatifloxacin | Pto - Protionamide | Eto - Ethionamide |
| Cs - Cycloserine | PAS - Para-aminosalicylic acid | | Fq -fluoroquinolone |  |

Multiple imputation results
