# Supplementary material for: Predictors of treatment outcomes among patients with multidrug-resistant tuberculosis in Vietnam: a retrospective cohort study
Source: BMC Infect Dis. 2022 Jan 20;22:68. doi: 10.1186/s12879-021-06992-x (PMC8772201; doi:10.1186/s12879-021-06992-x)
Supplement: Supplementary file 3 — Additional file 3. Multiple imputation parameters and results. [file 12879_2021_6992_MOESM3_ESM.docx]

**Additional File 3 – Multiple imputation results**

| **Imputation Specifications** | |
| --- | --- |
| Imputation Method | Automatic |
| Number of Imputations | 50 |
| Model for Scale Variables | Linear Regression |
| Interactions Included in Models | (none) |
| Maximum Percentage of Missing Values | 100.0% |
| Maximum Number of Parameters in Imputation Model | 100 |

| **Imputation Constraints** | | | | |
| --- | --- | --- | --- | --- |
|  | Role in Imputation | | Imputed Values | |
|  | Dependent | Predictor | Minimum | Maximum |
| Age | Yes | Yes | (none) | (none) |
| Gender | Yes | Yes |  |  |
| Previous_treatment | No | Yes |  |  |
| Payment_option | No | Yes |  |  |
| Comorbidity_diabetes | Yes | Yes |  |  |
| Comorbidity_heart_disease | Yes | Yes |  |  |
| Comorbidity_HIV | Yes | Yes |  |  |
| Comorbidity_kidney_disease | No | Yes |  |  |
| Comorbidity_liver_disease | No | Yes |  |  |
| Comorbidity_malnutrition | No | Yes |  |  |
| Comorbidity_psychiatric_disorder | No | Yes |  |  |
| Comorbidity_respiratory_disorder | Yes | Yes |  |  |
| Comorbidity_drugs_alcohol | Yes | Yes |  |  |
| Comorbidity_other | No | Yes |  |  |
| Infection_location | No | Yes |  |  |
| RR_MDRorPreXDR_XDR | Yes | Yes |  |  |
| Smear_positive | Yes | Yes |  |  |
| Smear_SCR_2months | Yes | Yes |  |  |
| Culture_SCR_2months | Yes | Yes |  |  |
| Smear_conv_4months | Yes | Yes |  |  |
| Culture_conv_4months | Yes | Yes |  |  |
| Inpatient_side_effect_experienced | Yes | Yes |  |  |
| Outpatient_side_effect_experienced | Yes | Yes |  |  |

**Imputed Values**

| **Imputation Results** | | |
| --- | --- | --- |
| Imputation Method | | Fully Conditional Specification |
| Fully Conditional Specification Method Iterations | | 10 |
| Dependent Variables | Imputed | Age,Comorbidity_diabetes,Comorbidity_heart_disease,Comorbidity_HIV,Comorbidity_respiratory_disorder,Comorbidity_drugs_alcohol,RR_MDRorPreXDR_XDR,Smear_positive,Smear_SCR_2months,Culture_SCR_2months,Smear_conv_4months,Culture_conv_4months,Inpatient_side_effect_experienced,Outpatient_side_effect_experienced |
|  | Not Imputed(Too Many Missing Values) |  |
|  | Not Imputed(No Missing Values) | Gender |
| Imputation Sequence | | Gender,Age,Infection_location,Previous_treatment,Smear_positive,Smear_SCR_2months,Outpatient_side_effect_experienced,Culture_SCR_2months,Smear_conv_4months,Culture_conv_4months,Comorbidity_HIV,Payment_option,Comorbidity_diabetes,Comorbidity_heart_disease,Comorbidity_kidney_disease,Comorbidity_liver_disease,Comorbidity_malnutrition,Comorbidity_psychiatric_disorder,Comorbidity_respiratory_disorder,Comorbidity_drugs_alcohol,Comorbidity_other,Inpatient_side_effect_experienced,RR_MDRorPreXDR_XDR |

| **Imputation Models** | | | | |
| --- | --- | --- | --- | --- |
|  | Model | | Missing Values | Imputed Values |
|  | Type | Effects |  |  |
| Age | Linear Regression | Gender,Infection_location,Previous_treatment,Smear_positive,Smear_SCR_2months,Outpatient_side_effect_experienced,Culture_SCR_2months,Smear_conv_4months,Culture_conv_4months,Comorbidity_HIV,Payment_option,Comorbidity_diabetes,Comorbidity_heart_disease,Comorbidity_kidney_disease,Comorbidity_liver_disease,Comorbidity_malnutrition,Comorbidity_psychiatric_disorder,Comorbidity_respiratory_disorder,Comorbidity_drugs_alcohol,Comorbidity_other,Inpatient_side_effect_experienced,RR_MDRorPreXDR_XDR | 1 | 50 |
| Infection_location | Logistic Regression | Gender,Previous_treatment,Smear_positive,Smear_SCR_2months,Outpatient_side_effect_experienced,Culture_SCR_2months,Smear_conv_4months,Culture_conv_4months,Comorbidity_HIV,Payment_option,Comorbidity_diabetes,Comorbidity_heart_disease,Comorbidity_kidney_disease,Comorbidity_liver_disease,Comorbidity_malnutrition,Comorbidity_psychiatric_disorder,Comorbidity_respiratory_disorder,Comorbidity_drugs_alcohol,Comorbidity_other,Inpatient_side_effect_experienced,RR_MDRorPreXDR_XDR,Age | 17 | 850^a^ |
| Previous_treatment | Logistic Regression | Gender,Infection_location,Smear_positive,Smear_SCR_2months,Outpatient_side_effect_experienced,Culture_SCR_2months,Smear_conv_4months,Culture_conv_4months,Comorbidity_HIV,Payment_option,Comorbidity_diabetes,Comorbidity_heart_disease,Comorbidity_kidney_disease,Comorbidity_liver_disease,Comorbidity_malnutrition,Comorbidity_psychiatric_disorder,Comorbidity_respiratory_disorder,Comorbidity_drugs_alcohol,Comorbidity_other,Inpatient_side_effect_experienced,RR_MDRorPreXDR_XDR,Age | 19 | 950^a^ |
| Smear_positive | Logistic Regression | Gender,Infection_location,Previous_treatment,Smear_SCR_2months,Outpatient_side_effect_experienced,Culture_SCR_2months,Smear_conv_4months,Culture_conv_4months,Comorbidity_HIV,Payment_option,Comorbidity_diabetes,Comorbidity_heart_disease,Comorbidity_kidney_disease,Comorbidity_liver_disease,Comorbidity_malnutrition,Comorbidity_psychiatric_disorder,Comorbidity_respiratory_disorder,Comorbidity_drugs_alcohol,Comorbidity_other,Inpatient_side_effect_experienced,RR_MDRorPreXDR_XDR,Age | 42 | 2100 |
| Smear_SCR_2months | Logistic Regression | Gender,Infection_location,Previous_treatment,Smear_positive,Outpatient_side_effect_experienced,Culture_SCR_2months,Smear_conv_4months,Culture_conv_4months,Comorbidity_HIV,Payment_option,Comorbidity_diabetes,Comorbidity_heart_disease,Comorbidity_kidney_disease,Comorbidity_liver_disease,Comorbidity_malnutrition,Comorbidity_psychiatric_disorder,Comorbidity_respiratory_disorder,Comorbidity_drugs_alcohol,Comorbidity_other,Inpatient_side_effect_experienced,RR_MDRorPreXDR_XDR,Age | 76 | 3800 |
| Outpatient_side_effect_experienced | Logistic Regression | Gender,Infection_location,Previous_treatment,Smear_positive,Smear_SCR_2months,Culture_SCR_2months,Smear_conv_4months,Culture_conv_4months,Comorbidity_HIV,Payment_option,Comorbidity_diabetes,Comorbidity_heart_disease,Comorbidity_kidney_disease,Comorbidity_liver_disease,Comorbidity_malnutrition,Comorbidity_psychiatric_disorder,Comorbidity_respiratory_disorder,Comorbidity_drugs_alcohol,Comorbidity_other,Inpatient_side_effect_experienced,RR_MDRorPreXDR_XDR,Age | 90 | 4500 |
| Culture_SCR_2months | Logistic Regression | Gender,Infection_location,Previous_treatment,Smear_positive,Smear_SCR_2months,Outpatient_side_effect_experienced,Smear_conv_4months,Culture_conv_4months,Comorbidity_HIV,Payment_option,Comorbidity_diabetes,Comorbidity_heart_disease,Comorbidity_kidney_disease,Comorbidity_liver_disease,Comorbidity_malnutrition,Comorbidity_psychiatric_disorder,Comorbidity_respiratory_disorder,Comorbidity_drugs_alcohol,Comorbidity_other,Inpatient_side_effect_experienced,RR_MDRorPreXDR_XDR,Age | 109 | 5450 |
| Smear_conv_4months | Logistic Regression | Gender,Infection_location,Previous_treatment,Smear_positive,Smear_SCR_2months,Outpatient_side_effect_experienced,Culture_SCR_2months,Culture_conv_4months,Comorbidity_HIV,Payment_option,Comorbidity_diabetes,Comorbidity_heart_disease,Comorbidity_kidney_disease,Comorbidity_liver_disease,Comorbidity_malnutrition,Comorbidity_psychiatric_disorder,Comorbidity_respiratory_disorder,Comorbidity_drugs_alcohol,Comorbidity_other,Inpatient_side_effect_experienced,RR_MDRorPreXDR_XDR,Age | 125 | 6250 |
| Culture_conv_4months | Logistic Regression | Gender,Infection_location,Previous_treatment,Smear_positive,Smear_SCR_2months,Outpatient_side_effect_experienced,Culture_SCR_2months,Smear_conv_4months,Comorbidity_HIV,Payment_option,Comorbidity_diabetes,Comorbidity_heart_disease,Comorbidity_kidney_disease,Comorbidity_liver_disease,Comorbidity_malnutrition,Comorbidity_psychiatric_disorder,Comorbidity_respiratory_disorder,Comorbidity_drugs_alcohol,Comorbidity_other,Inpatient_side_effect_experienced,RR_MDRorPreXDR_XDR,Age | 134 | 6700 |
| Comorbidity_HIV | Logistic Regression | Gender,Infection_location,Previous_treatment,Smear_positive,Smear_SCR_2months,Outpatient_side_effect_experienced,Culture_SCR_2months,Smear_conv_4months,Culture_conv_4months,Payment_option,Comorbidity_diabetes,Comorbidity_heart_disease,Comorbidity_kidney_disease,Comorbidity_liver_disease,Comorbidity_malnutrition,Comorbidity_psychiatric_disorder,Comorbidity_respiratory_disorder,Comorbidity_drugs_alcohol,Comorbidity_other,Inpatient_side_effect_experienced,RR_MDRorPreXDR_XDR,Age | 135 | 6750 |
| Payment_option | Logistic Regression | Gender,Infection_location,Previous_treatment,Smear_positive,Smear_SCR_2months,Outpatient_side_effect_experienced,Culture_SCR_2months,Smear_conv_4months,Culture_conv_4months,Comorbidity_HIV,Comorbidity_diabetes,Comorbidity_heart_disease,Comorbidity_kidney_disease,Comorbidity_liver_disease,Comorbidity_malnutrition,Comorbidity_psychiatric_disorder,Comorbidity_respiratory_disorder,Comorbidity_drugs_alcohol,Comorbidity_other,Inpatient_side_effect_experienced,RR_MDRorPreXDR_XDR,Age | 204 | 10200^a^ |
| Comorbidity_diabetes | Logistic Regression | Gender,Infection_location,Previous_treatment,Smear_positive,Smear_SCR_2months,Outpatient_side_effect_experienced,Culture_SCR_2months,Smear_conv_4months,Culture_conv_4months,Comorbidity_HIV,Payment_option,Comorbidity_heart_disease,Comorbidity_kidney_disease,Comorbidity_liver_disease,Comorbidity_malnutrition,Comorbidity_psychiatric_disorder,Comorbidity_respiratory_disorder,Comorbidity_drugs_alcohol,Comorbidity_other,Inpatient_side_effect_experienced,RR_MDRorPreXDR_XDR,Age | 204 | 10200 |
| Comorbidity_heart_disease | Logistic Regression | Gender,Infection_location,Previous_treatment,Smear_positive,Smear_SCR_2months,Outpatient_side_effect_experienced,Culture_SCR_2months,Smear_conv_4months,Culture_conv_4months,Comorbidity_HIV,Payment_option,Comorbidity_diabetes,Comorbidity_kidney_disease,Comorbidity_liver_disease,Comorbidity_malnutrition,Comorbidity_psychiatric_disorder,Comorbidity_respiratory_disorder,Comorbidity_drugs_alcohol,Comorbidity_other,Inpatient_side_effect_experienced,RR_MDRorPreXDR_XDR,Age | 204 | 10200 |
| Comorbidity_kidney_disease | Logistic Regression | Gender,Infection_location,Previous_treatment,Smear_positive,Smear_SCR_2months,Outpatient_side_effect_experienced,Culture_SCR_2months,Smear_conv_4months,Culture_conv_4months,Comorbidity_HIV,Payment_option,Comorbidity_diabetes,Comorbidity_heart_disease,Comorbidity_liver_disease,Comorbidity_malnutrition,Comorbidity_psychiatric_disorder,Comorbidity_respiratory_disorder,Comorbidity_drugs_alcohol,Comorbidity_other,Inpatient_side_effect_experienced,RR_MDRorPreXDR_XDR,Age | 204 | 10200^a^ |
| Comorbidity_liver_disease | Logistic Regression | Gender,Infection_location,Previous_treatment,Smear_positive,Smear_SCR_2months,Outpatient_side_effect_experienced,Culture_SCR_2months,Smear_conv_4months,Culture_conv_4months,Comorbidity_HIV,Payment_option,Comorbidity_diabetes,Comorbidity_heart_disease,Comorbidity_kidney_disease,Comorbidity_malnutrition,Comorbidity_psychiatric_disorder,Comorbidity_respiratory_disorder,Comorbidity_drugs_alcohol,Comorbidity_other,Inpatient_side_effect_experienced,RR_MDRorPreXDR_XDR,Age | 204 | 10200^a^ |
| Comorbidity_malnutrition | Logistic Regression | Gender,Infection_location,Previous_treatment,Smear_positive,Smear_SCR_2months,Outpatient_side_effect_experienced,Culture_SCR_2months,Smear_conv_4months,Culture_conv_4months,Comorbidity_HIV,Payment_option,Comorbidity_diabetes,Comorbidity_heart_disease,Comorbidity_kidney_disease,Comorbidity_liver_disease,Comorbidity_psychiatric_disorder,Comorbidity_respiratory_disorder,Comorbidity_drugs_alcohol,Comorbidity_other,Inpatient_side_effect_experienced,RR_MDRorPreXDR_XDR,Age | 204 | 10200^a^ |
| Comorbidity_psychiatric_disorder | Logistic Regression | Gender,Infection_location,Previous_treatment,Smear_positive,Smear_SCR_2months,Outpatient_side_effect_experienced,Culture_SCR_2months,Smear_conv_4months,Culture_conv_4months,Comorbidity_HIV,Payment_option,Comorbidity_diabetes,Comorbidity_heart_disease,Comorbidity_kidney_disease,Comorbidity_liver_disease,Comorbidity_malnutrition,Comorbidity_respiratory_disorder,Comorbidity_drugs_alcohol,Comorbidity_other,Inpatient_side_effect_experienced,RR_MDRorPreXDR_XDR,Age | 204 | 10200^a^ |
| Comorbidity_respiratory_disorder | Logistic Regression | Gender,Infection_location,Previous_treatment,Smear_positive,Smear_SCR_2months,Outpatient_side_effect_experienced,Culture_SCR_2months,Smear_conv_4months,Culture_conv_4months,Comorbidity_HIV,Payment_option,Comorbidity_diabetes,Comorbidity_heart_disease,Comorbidity_kidney_disease,Comorbidity_liver_disease,Comorbidity_malnutrition,Comorbidity_psychiatric_disorder,Comorbidity_drugs_alcohol,Comorbidity_other,Inpatient_side_effect_experienced,RR_MDRorPreXDR_XDR,Age | 204 | 10200 |
| Comorbidity_drugs_alcohol | Logistic Regression | Gender,Infection_location,Previous_treatment,Smear_positive,Smear_SCR_2months,Outpatient_side_effect_experienced,Culture_SCR_2months,Smear_conv_4months,Culture_conv_4months,Comorbidity_HIV,Payment_option,Comorbidity_diabetes,Comorbidity_heart_disease,Comorbidity_kidney_disease,Comorbidity_liver_disease,Comorbidity_malnutrition,Comorbidity_psychiatric_disorder,Comorbidity_respiratory_disorder,Comorbidity_other,Inpatient_side_effect_experienced,RR_MDRorPreXDR_XDR,Age | 204 | 10200 |
| Comorbidity_other | Logistic Regression | Gender,Infection_location,Previous_treatment,Smear_positive,Smear_SCR_2months,Outpatient_side_effect_experienced,Culture_SCR_2months,Smear_conv_4months,Culture_conv_4months,Comorbidity_HIV,Payment_option,Comorbidity_diabetes,Comorbidity_heart_disease,Comorbidity_kidney_disease,Comorbidity_liver_disease,Comorbidity_malnutrition,Comorbidity_psychiatric_disorder,Comorbidity_respiratory_disorder,Comorbidity_drugs_alcohol,Inpatient_side_effect_experienced,RR_MDRorPreXDR_XDR,Age | 204 | 10200^a^ |
| Inpatient_side_effect_experienced | Logistic Regression | Gender,Infection_location,Previous_treatment,Smear_positive,Smear_SCR_2months,Outpatient_side_effect_experienced,Culture_SCR_2months,Smear_conv_4months,Culture_conv_4months,Comorbidity_HIV,Payment_option,Comorbidity_diabetes,Comorbidity_heart_disease,Comorbidity_kidney_disease,Comorbidity_liver_disease,Comorbidity_malnutrition,Comorbidity_psychiatric_disorder,Comorbidity_respiratory_disorder,Comorbidity_drugs_alcohol,Comorbidity_other,RR_MDRorPreXDR_XDR,Age | 210 | 10500 |
| RR_MDRorPreXDR_XDR | Logistic Regression | Gender,Infection_location,Previous_treatment,Smear_positive,Smear_SCR_2months,Outpatient_side_effect_experienced,Culture_SCR_2months,Smear_conv_4months,Culture_conv_4months,Comorbidity_HIV,Payment_option,Comorbidity_diabetes,Comorbidity_heart_disease,Comorbidity_kidney_disease,Comorbidity_liver_disease,Comorbidity_malnutrition,Comorbidity_psychiatric_disorder,Comorbidity_respiratory_disorder,Comorbidity_drugs_alcohol,Comorbidity_other,Inpatient_side_effect_experienced,Age | 221 | 11050 |

| a. This variable with role as predictor only has missing values which were imputed for internal purposes. |
| --- |

**Descriptive Statistics**

| **Age** | | | | | | |
| --- | --- | --- | --- | --- | --- | --- |
| Data | Imputation | N | Mean | Std. Deviation | Minimum | Maximum |
| Original Data |  | 661 | 43.89 | 14.582 | 15.00 | 85.00 |
| Imputed Values | 1 | 1 | 31.57 | . | 31.57 | 31.57 |
|  | 2 | 1 | 55.95 | . | 55.95 | 55.95 |
|  | 3 | 1 | 32.61 | . | 32.61 | 32.61 |
|  | 4 | 1 | 48.03 | . | 48.03 | 48.03 |
|  | 5 | 1 | 35.98 | . | 35.98 | 35.98 |
|  | 6 | 1 | 44.93 | . | 44.93 | 44.93 |
|  | 7 | 1 | 40.93 | . | 40.93 | 40.93 |
|  | 8 | 1 | 28.61 | . | 28.61 | 28.61 |
|  | 9 | 1 | 73.41 | . | 73.41 | 73.41 |
|  | 10 | 1 | 51.10 | . | 51.10 | 51.10 |
|  | 11 | 1 | 40.39 | . | 40.39 | 40.39 |
|  | 12 | 1 | 55.64 | . | 55.64 | 55.64 |
|  | 13 | 1 | 43.72 | . | 43.72 | 43.72 |
|  | 14 | 1 | 39.99 | . | 39.99 | 39.99 |
|  | 15 | 1 | 38.82 | . | 38.82 | 38.82 |
|  | 16 | 1 | 61.50 | . | 61.50 | 61.50 |
|  | 17 | 1 | 45.81 | . | 45.81 | 45.81 |
|  | 18 | 1 | 21.42 | . | 21.42 | 21.42 |
|  | 19 | 1 | 47.03 | . | 47.03 | 47.03 |
|  | 20 | 1 | 44.15 | . | 44.15 | 44.15 |
|  | 21 | 1 | 54.45 | . | 54.45 | 54.45 |
|  | 22 | 1 | 47.63 | . | 47.63 | 47.63 |
|  | 23 | 1 | 13.88 | . | 13.88 | 13.88 |
|  | 24 | 1 | 39.52 | . | 39.52 | 39.52 |
|  | 25 | 1 | 30.47 | . | 30.47 | 30.47 |
|  | 26 | 1 | 46.87 | . | 46.87 | 46.87 |
|  | 27 | 1 | 37.34 | . | 37.34 | 37.34 |
|  | 28 | 1 | 52.55 | . | 52.55 | 52.55 |
|  | 29 | 1 | 27.16 | . | 27.16 | 27.16 |
|  | 30 | 1 | 37.86 | . | 37.86 | 37.86 |
|  | 31 | 1 | 50.13 | . | 50.13 | 50.13 |
|  | 32 | 1 | 36.53 | . | 36.53 | 36.53 |
|  | 33 | 1 | 24.31 | . | 24.31 | 24.31 |
|  | 34 | 1 | 32.48 | . | 32.48 | 32.48 |
|  | 35 | 1 | 53.83 | . | 53.83 | 53.83 |
|  | 36 | 1 | 22.96 | . | 22.96 | 22.96 |
|  | 37 | 1 | 63.72 | . | 63.72 | 63.72 |
|  | 38 | 1 | 69.42 | . | 69.42 | 69.42 |
|  | 39 | 1 | 27.21 | . | 27.21 | 27.21 |
|  | 40 | 1 | 34.67 | . | 34.67 | 34.67 |
|  | 41 | 1 | 55.18 | . | 55.18 | 55.18 |
|  | 42 | 1 | 27.61 | . | 27.61 | 27.61 |
|  | 43 | 1 | 40.39 | . | 40.39 | 40.39 |
|  | 44 | 1 | 13.00 | . | 13.00 | 13.00 |
|  | 45 | 1 | 40.89 | . | 40.89 | 40.89 |
|  | 46 | 1 | 39.02 | . | 39.02 | 39.02 |
|  | 47 | 1 | 66.54 | . | 66.54 | 66.54 |
|  | 48 | 1 | 40.37 | . | 40.37 | 40.37 |
|  | 49 | 1 | 45.68 | . | 45.68 | 45.68 |
|  | 50 | 1 | 74.89 | . | 74.89 | 74.89 |
| Complete Data After Imputation | 1 | 662 | 43.87 | 14.579 | 15.00 | 85.00 |
|  | 2 | 662 | 43.91 | 14.578 | 15.00 | 85.00 |
|  | 3 | 662 | 43.87 | 14.577 | 15.00 | 85.00 |
|  | 4 | 662 | 43.89 | 14.572 | 15.00 | 85.00 |
|  | 5 | 662 | 43.88 | 14.574 | 15.00 | 85.00 |
|  | 6 | 662 | 43.89 | 14.571 | 15.00 | 85.00 |
|  | 7 | 662 | 43.88 | 14.571 | 15.00 | 85.00 |
|  | 8 | 662 | 43.86 | 14.583 | 15.00 | 85.00 |
|  | 9 | 662 | 43.93 | 14.616 | 15.00 | 85.00 |
|  | 10 | 662 | 43.90 | 14.573 | 15.00 | 85.00 |
|  | 11 | 662 | 43.88 | 14.571 | 15.00 | 85.00 |
|  | 12 | 662 | 43.91 | 14.578 | 15.00 | 85.00 |
|  | 13 | 662 | 43.89 | 14.571 | 15.00 | 85.00 |
|  | 14 | 662 | 43.88 | 14.571 | 15.00 | 85.00 |
|  | 15 | 662 | 43.88 | 14.572 | 15.00 | 85.00 |
|  | 16 | 662 | 43.91 | 14.587 | 15.00 | 85.00 |
|  | 17 | 662 | 43.89 | 14.571 | 15.00 | 85.00 |
|  | 18 | 662 | 43.85 | 14.597 | 15.00 | 85.00 |
|  | 19 | 662 | 43.89 | 14.571 | 15.00 | 85.00 |
|  | 20 | 662 | 43.89 | 14.571 | 15.00 | 85.00 |
|  | 21 | 662 | 43.90 | 14.576 | 15.00 | 85.00 |
|  | 22 | 662 | 43.89 | 14.571 | 15.00 | 85.00 |
|  | 23 | 662 | 43.84 | 14.617 | 13.88 | 85.00 |
|  | 24 | 662 | 43.88 | 14.572 | 15.00 | 85.00 |
|  | 25 | 662 | 43.87 | 14.580 | 15.00 | 85.00 |
|  | 26 | 662 | 43.89 | 14.571 | 15.00 | 85.00 |
|  | 27 | 662 | 43.88 | 14.573 | 15.00 | 85.00 |
|  | 28 | 662 | 43.90 | 14.575 | 15.00 | 85.00 |
|  | 29 | 662 | 43.86 | 14.585 | 15.00 | 85.00 |
|  | 30 | 662 | 43.88 | 14.573 | 15.00 | 85.00 |
|  | 31 | 662 | 43.90 | 14.573 | 15.00 | 85.00 |
|  | 32 | 662 | 43.88 | 14.574 | 15.00 | 85.00 |
|  | 33 | 662 | 43.86 | 14.591 | 15.00 | 85.00 |
|  | 34 | 662 | 43.87 | 14.577 | 15.00 | 85.00 |
|  | 35 | 662 | 43.90 | 14.576 | 15.00 | 85.00 |
|  | 36 | 662 | 43.86 | 14.593 | 15.00 | 85.00 |
|  | 37 | 662 | 43.92 | 14.591 | 15.00 | 85.00 |
|  | 38 | 662 | 43.93 | 14.604 | 15.00 | 85.00 |
|  | 39 | 662 | 43.86 | 14.585 | 15.00 | 85.00 |
|  | 40 | 662 | 43.87 | 14.575 | 15.00 | 85.00 |
|  | 41 | 662 | 43.91 | 14.577 | 15.00 | 85.00 |
|  | 42 | 662 | 43.86 | 14.584 | 15.00 | 85.00 |
|  | 43 | 662 | 43.88 | 14.571 | 15.00 | 85.00 |
|  | 44 | 662 | 43.84 | 14.620 | 13.00 | 85.00 |
|  | 45 | 662 | 43.88 | 14.571 | 15.00 | 85.00 |
|  | 46 | 662 | 43.88 | 14.572 | 15.00 | 85.00 |
|  | 47 | 662 | 43.92 | 14.597 | 15.00 | 85.00 |
|  | 48 | 662 | 43.88 | 14.571 | 15.00 | 85.00 |
|  | 49 | 662 | 43.89 | 14.571 | 15.00 | 85.00 |
|  | 50 | 662 | 43.93 | 14.620 | 15.00 | 85.00 |

| **Comorbidity_diabetes** | | | | |
| --- | --- | --- | --- | --- |
| Data | Imputation | Category | N | Percent |
| Original Data |  | 0 | 406 | 88.6 |
|  |  | 1 | 52 | 11.4 |
| Imputed Values | 1 | 0 | 18 | 8.8 |
|  |  | 1 | 186 | 91.2 |
|  | 2 | 0 | 192 | 94.1 |
|  |  | 1 | 12 | 5.9 |
|  | 3 | 0 | 168 | 82.4 |
|  |  | 1 | 36 | 17.6 |
|  | 4 | 0 | 191 | 93.6 |
|  |  | 1 | 13 | 6.4 |
|  | 5 | 0 | 194 | 95.1 |
|  |  | 1 | 10 | 4.9 |
|  | 6 | 0 | 137 | 67.2 |
|  |  | 1 | 67 | 32.8 |
|  | 7 | 0 | 16 | 7.8 |
|  |  | 1 | 188 | 92.2 |
|  | 8 | 0 | 114 | 55.9 |
|  |  | 1 | 90 | 44.1 |
|  | 9 | 0 | 151 | 74.0 |
|  |  | 1 | 53 | 26.0 |
|  | 10 | 0 | 80 | 39.2 |
|  |  | 1 | 124 | 60.8 |
|  | 11 | 0 | 83 | 40.7 |
|  |  | 1 | 121 | 59.3 |
|  | 12 | 0 | 186 | 91.2 |
|  |  | 1 | 18 | 8.8 |
|  | 13 | 0 | 199 | 97.5 |
|  |  | 1 | 5 | 2.5 |
|  | 14 | 0 | 88 | 43.1 |
|  |  | 1 | 116 | 56.9 |
|  | 15 | 0 | 86 | 42.2 |
|  |  | 1 | 118 | 57.8 |
|  | 16 | 0 | 94 | 46.1 |
|  |  | 1 | 110 | 53.9 |
|  | 17 | 0 | 31 | 15.2 |
|  |  | 1 | 173 | 84.8 |
|  | 18 | 0 | 151 | 74.0 |
|  |  | 1 | 53 | 26.0 |
|  | 19 | 0 | 172 | 84.3 |
|  |  | 1 | 32 | 15.7 |
|  | 20 | 0 | 64 | 31.4 |
|  |  | 1 | 140 | 68.6 |
|  | 21 | 0 | 204 | 100.0 |
|  | 22 | 0 | 67 | 32.8 |
|  |  | 1 | 137 | 67.2 |
|  | 23 | 0 | 176 | 86.3 |
|  |  | 1 | 28 | 13.7 |
|  | 24 | 0 | 16 | 7.8 |
|  |  | 1 | 188 | 92.2 |
|  | 25 | 0 | 12 | 5.9 |
|  |  | 1 | 192 | 94.1 |
|  | 26 | 0 | 180 | 88.2 |
|  |  | 1 | 24 | 11.8 |
|  | 27 | 0 | 194 | 95.1 |
|  |  | 1 | 10 | 4.9 |
|  | 28 | 0 | 85 | 41.7 |
|  |  | 1 | 119 | 58.3 |
|  | 29 | 0 | 68 | 33.3 |
|  |  | 1 | 136 | 66.7 |
|  | 30 | 0 | 154 | 75.5 |
|  |  | 1 | 50 | 24.5 |
|  | 31 | 0 | 32 | 15.7 |
|  |  | 1 | 172 | 84.3 |
|  | 32 | 0 | 65 | 31.9 |
|  |  | 1 | 139 | 68.1 |
|  | 33 | 0 | 188 | 92.2 |
|  |  | 1 | 16 | 7.8 |
|  | 34 | 0 | 162 | 79.4 |
|  |  | 1 | 42 | 20.6 |
|  | 35 | 0 | 167 | 81.9 |
|  |  | 1 | 37 | 18.1 |
|  | 36 | 0 | 21 | 10.3 |
|  |  | 1 | 183 | 89.7 |
|  | 37 | 0 | 169 | 82.8 |
|  |  | 1 | 35 | 17.2 |
|  | 38 | 0 | 21 | 10.3 |
|  |  | 1 | 183 | 89.7 |
|  | 39 | 0 | 4 | 2.0 |
|  |  | 1 | 200 | 98.0 |
|  | 40 | 0 | 17 | 8.3 |
|  |  | 1 | 187 | 91.7 |
|  | 41 | 0 | 13 | 6.4 |
|  |  | 1 | 191 | 93.6 |
|  | 42 | 0 | 136 | 66.7 |
|  |  | 1 | 68 | 33.3 |
|  | 43 | 0 | 109 | 53.4 |
|  |  | 1 | 95 | 46.6 |
|  | 44 | 0 | 42 | 20.6 |
|  |  | 1 | 162 | 79.4 |
|  | 45 | 0 | 47 | 23.0 |
|  |  | 1 | 157 | 77.0 |
|  | 46 | 1 | 204 | 100.0 |
|  | 47 | 0 | 198 | 97.1 |
|  |  | 1 | 6 | 2.9 |
|  | 48 | 0 | 5 | 2.5 |
|  |  | 1 | 199 | 97.5 |
|  | 49 | 0 | 33 | 16.2 |
|  |  | 1 | 171 | 83.8 |
|  | 50 | 0 | 74 | 36.3 |
|  |  | 1 | 130 | 63.7 |
| Complete Data After Imputation | 1 | 0 | 424 | 64.0 |
|  |  | 1 | 238 | 36.0 |
|  | 2 | 0 | 598 | 90.3 |
|  |  | 1 | 64 | 9.7 |
|  | 3 | 0 | 574 | 86.7 |
|  |  | 1 | 88 | 13.3 |
|  | 4 | 0 | 597 | 90.2 |
|  |  | 1 | 65 | 9.8 |
|  | 5 | 0 | 600 | 90.6 |
|  |  | 1 | 62 | 9.4 |
|  | 6 | 0 | 543 | 82.0 |
|  |  | 1 | 119 | 18.0 |
|  | 7 | 0 | 422 | 63.7 |
|  |  | 1 | 240 | 36.3 |
|  | 8 | 0 | 520 | 78.5 |
|  |  | 1 | 142 | 21.5 |
|  | 9 | 0 | 557 | 84.1 |
|  |  | 1 | 105 | 15.9 |
|  | 10 | 0 | 486 | 73.4 |
|  |  | 1 | 176 | 26.6 |
|  | 11 | 0 | 489 | 73.9 |
|  |  | 1 | 173 | 26.1 |
|  | 12 | 0 | 592 | 89.4 |
|  |  | 1 | 70 | 10.6 |
|  | 13 | 0 | 605 | 91.4 |
|  |  | 1 | 57 | 8.6 |
|  | 14 | 0 | 494 | 74.6 |
|  |  | 1 | 168 | 25.4 |
|  | 15 | 0 | 492 | 74.3 |
|  |  | 1 | 170 | 25.7 |
|  | 16 | 0 | 500 | 75.5 |
|  |  | 1 | 162 | 24.5 |
|  | 17 | 0 | 437 | 66.0 |
|  |  | 1 | 225 | 34.0 |
|  | 18 | 0 | 557 | 84.1 |
|  |  | 1 | 105 | 15.9 |
|  | 19 | 0 | 578 | 87.3 |
|  |  | 1 | 84 | 12.7 |
|  | 20 | 0 | 470 | 71.0 |
|  |  | 1 | 192 | 29.0 |
|  | 21 | 0 | 610 | 92.1 |
|  |  | 1 | 52 | 7.9 |
|  | 22 | 0 | 473 | 71.5 |
|  |  | 1 | 189 | 28.5 |
|  | 23 | 0 | 582 | 87.9 |
|  |  | 1 | 80 | 12.1 |
|  | 24 | 0 | 422 | 63.7 |
|  |  | 1 | 240 | 36.3 |
|  | 25 | 0 | 418 | 63.1 |
|  |  | 1 | 244 | 36.9 |
|  | 26 | 0 | 586 | 88.5 |
|  |  | 1 | 76 | 11.5 |
|  | 27 | 0 | 600 | 90.6 |
|  |  | 1 | 62 | 9.4 |
|  | 28 | 0 | 491 | 74.2 |
|  |  | 1 | 171 | 25.8 |
|  | 29 | 0 | 474 | 71.6 |
|  |  | 1 | 188 | 28.4 |
|  | 30 | 0 | 560 | 84.6 |
|  |  | 1 | 102 | 15.4 |
|  | 31 | 0 | 438 | 66.2 |
|  |  | 1 | 224 | 33.8 |
|  | 32 | 0 | 471 | 71.1 |
|  |  | 1 | 191 | 28.9 |
|  | 33 | 0 | 594 | 89.7 |
|  |  | 1 | 68 | 10.3 |
|  | 34 | 0 | 568 | 85.8 |
|  |  | 1 | 94 | 14.2 |
|  | 35 | 0 | 573 | 86.6 |
|  |  | 1 | 89 | 13.4 |
|  | 36 | 0 | 427 | 64.5 |
|  |  | 1 | 235 | 35.5 |
|  | 37 | 0 | 575 | 86.9 |
|  |  | 1 | 87 | 13.1 |
|  | 38 | 0 | 427 | 64.5 |
|  |  | 1 | 235 | 35.5 |
|  | 39 | 0 | 410 | 61.9 |
|  |  | 1 | 252 | 38.1 |
|  | 40 | 0 | 423 | 63.9 |
|  |  | 1 | 239 | 36.1 |
|  | 41 | 0 | 419 | 63.3 |
|  |  | 1 | 243 | 36.7 |
|  | 42 | 0 | 542 | 81.9 |
|  |  | 1 | 120 | 18.1 |
|  | 43 | 0 | 515 | 77.8 |
|  |  | 1 | 147 | 22.2 |
|  | 44 | 0 | 448 | 67.7 |
|  |  | 1 | 214 | 32.3 |
|  | 45 | 0 | 453 | 68.4 |
|  |  | 1 | 209 | 31.6 |
|  | 46 | 0 | 406 | 61.3 |
|  |  | 1 | 256 | 38.7 |
|  | 47 | 0 | 604 | 91.2 |
|  |  | 1 | 58 | 8.8 |
|  | 48 | 0 | 411 | 62.1 |
|  |  | 1 | 251 | 37.9 |
|  | 49 | 0 | 439 | 66.3 |
|  |  | 1 | 223 | 33.7 |
|  | 50 | 0 | 480 | 72.5 |
|  |  | 1 | 182 | 27.5 |

| **Comorbidity_heart_disease** | | | | |
| --- | --- | --- | --- | --- |
| Data | Imputation | Category | N | Percent |
| Original Data |  | 0 | 440 | 96.1 |
|  |  | 1 | 18 | 3.9 |
| Imputed Values | 1 | 0 | 3 | 1.5 |
|  |  | 1 | 201 | 98.5 |
|  | 2 | 0 | 204 | 100.0 |
|  | 3 | 0 | 1 | .5 |
|  |  | 1 | 203 | 99.5 |
|  | 4 | 1 | 204 | 100.0 |
|  | 5 | 0 | 107 | 52.5 |
|  |  | 1 | 97 | 47.5 |
|  | 6 | 0 | 39 | 19.1 |
|  |  | 1 | 165 | 80.9 |
|  | 7 | 0 | 169 | 82.8 |
|  |  | 1 | 35 | 17.2 |
|  | 8 | 0 | 27 | 13.2 |
|  |  | 1 | 177 | 86.8 |
|  | 9 | 1 | 204 | 100.0 |
|  | 10 | 0 | 45 | 22.1 |
|  |  | 1 | 159 | 77.9 |
|  | 11 | 0 | 115 | 56.4 |
|  |  | 1 | 89 | 43.6 |
|  | 12 | 0 | 173 | 84.8 |
|  |  | 1 | 31 | 15.2 |
|  | 13 | 0 | 187 | 91.7 |
|  |  | 1 | 17 | 8.3 |
|  | 14 | 0 | 151 | 74.0 |
|  |  | 1 | 53 | 26.0 |
|  | 15 | 0 | 150 | 73.5 |
|  |  | 1 | 54 | 26.5 |
|  | 16 | 0 | 186 | 91.2 |
|  |  | 1 | 18 | 8.8 |
|  | 17 | 0 | 162 | 79.4 |
|  |  | 1 | 42 | 20.6 |
|  | 18 | 0 | 7 | 3.4 |
|  |  | 1 | 197 | 96.6 |
|  | 19 | 0 | 99 | 48.5 |
|  |  | 1 | 105 | 51.5 |
|  | 20 | 0 | 196 | 96.1 |
|  |  | 1 | 8 | 3.9 |
|  | 21 | 0 | 183 | 89.7 |
|  |  | 1 | 21 | 10.3 |
|  | 22 | 0 | 165 | 80.9 |
|  |  | 1 | 39 | 19.1 |
|  | 23 | 0 | 163 | 79.9 |
|  |  | 1 | 41 | 20.1 |
|  | 24 | 0 | 204 | 100.0 |
|  | 25 | 0 | 167 | 81.9 |
|  |  | 1 | 37 | 18.1 |
|  | 26 | 0 | 76 | 37.3 |
|  |  | 1 | 128 | 62.7 |
|  | 27 | 0 | 88 | 43.1 |
|  |  | 1 | 116 | 56.9 |
|  | 28 | 0 | 8 | 3.9 |
|  |  | 1 | 196 | 96.1 |
|  | 29 | 0 | 203 | 99.5 |
|  |  | 1 | 1 | .5 |
|  | 30 | 0 | 203 | 99.5 |
|  |  | 1 | 1 | .5 |
|  | 31 | 0 | 100 | 49.0 |
|  |  | 1 | 104 | 51.0 |
|  | 32 | 0 | 202 | 99.0 |
|  |  | 1 | 2 | 1.0 |
|  | 33 | 0 | 86 | 42.2 |
|  |  | 1 | 118 | 57.8 |
|  | 34 | 0 | 83 | 40.7 |
|  |  | 1 | 121 | 59.3 |
|  | 35 | 0 | 148 | 72.5 |
|  |  | 1 | 56 | 27.5 |
|  | 36 | 0 | 3 | 1.5 |
|  |  | 1 | 201 | 98.5 |
|  | 37 | 0 | 98 | 48.0 |
|  |  | 1 | 106 | 52.0 |
|  | 38 | 0 | 140 | 68.6 |
|  |  | 1 | 64 | 31.4 |
|  | 39 | 0 | 185 | 90.7 |
|  |  | 1 | 19 | 9.3 |
|  | 40 | 0 | 23 | 11.3 |
|  |  | 1 | 181 | 88.7 |
|  | 41 | 0 | 187 | 91.7 |
|  |  | 1 | 17 | 8.3 |
|  | 42 | 0 | 68 | 33.3 |
|  |  | 1 | 136 | 66.7 |
|  | 43 | 0 | 200 | 98.0 |
|  |  | 1 | 4 | 2.0 |
|  | 44 | 0 | 40 | 19.6 |
|  |  | 1 | 164 | 80.4 |
|  | 45 | 0 | 87 | 42.6 |
|  |  | 1 | 117 | 57.4 |
|  | 46 | 0 | 23 | 11.3 |
|  |  | 1 | 181 | 88.7 |
|  | 47 | 0 | 16 | 7.8 |
|  |  | 1 | 188 | 92.2 |
|  | 48 | 0 | 127 | 62.3 |
|  |  | 1 | 77 | 37.7 |
|  | 49 | 0 | 71 | 34.8 |
|  |  | 1 | 133 | 65.2 |
|  | 50 | 0 | 169 | 82.8 |
|  |  | 1 | 35 | 17.2 |
| Complete Data After Imputation | 1 | 0 | 443 | 66.9 |
|  |  | 1 | 219 | 33.1 |
|  | 2 | 0 | 644 | 97.3 |
|  |  | 1 | 18 | 2.7 |
|  | 3 | 0 | 441 | 66.6 |
|  |  | 1 | 221 | 33.4 |
|  | 4 | 0 | 440 | 66.5 |
|  |  | 1 | 222 | 33.5 |
|  | 5 | 0 | 547 | 82.6 |
|  |  | 1 | 115 | 17.4 |
|  | 6 | 0 | 479 | 72.4 |
|  |  | 1 | 183 | 27.6 |
|  | 7 | 0 | 609 | 92.0 |
|  |  | 1 | 53 | 8.0 |
|  | 8 | 0 | 467 | 70.5 |
|  |  | 1 | 195 | 29.5 |
|  | 9 | 0 | 440 | 66.5 |
|  |  | 1 | 222 | 33.5 |
|  | 10 | 0 | 485 | 73.3 |
|  |  | 1 | 177 | 26.7 |
|  | 11 | 0 | 555 | 83.8 |
|  |  | 1 | 107 | 16.2 |
|  | 12 | 0 | 613 | 92.6 |
|  |  | 1 | 49 | 7.4 |
|  | 13 | 0 | 627 | 94.7 |
|  |  | 1 | 35 | 5.3 |
|  | 14 | 0 | 591 | 89.3 |
|  |  | 1 | 71 | 10.7 |
|  | 15 | 0 | 590 | 89.1 |
|  |  | 1 | 72 | 10.9 |
|  | 16 | 0 | 626 | 94.6 |
|  |  | 1 | 36 | 5.4 |
|  | 17 | 0 | 602 | 90.9 |
|  |  | 1 | 60 | 9.1 |
|  | 18 | 0 | 447 | 67.5 |
|  |  | 1 | 215 | 32.5 |
|  | 19 | 0 | 539 | 81.4 |
|  |  | 1 | 123 | 18.6 |
|  | 20 | 0 | 636 | 96.1 |
|  |  | 1 | 26 | 3.9 |
|  | 21 | 0 | 623 | 94.1 |
|  |  | 1 | 39 | 5.9 |
|  | 22 | 0 | 605 | 91.4 |
|  |  | 1 | 57 | 8.6 |
|  | 23 | 0 | 603 | 91.1 |
|  |  | 1 | 59 | 8.9 |
|  | 24 | 0 | 644 | 97.3 |
|  |  | 1 | 18 | 2.7 |
|  | 25 | 0 | 607 | 91.7 |
|  |  | 1 | 55 | 8.3 |
|  | 26 | 0 | 516 | 77.9 |
|  |  | 1 | 146 | 22.1 |
|  | 27 | 0 | 528 | 79.8 |
|  |  | 1 | 134 | 20.2 |
|  | 28 | 0 | 448 | 67.7 |
|  |  | 1 | 214 | 32.3 |
|  | 29 | 0 | 643 | 97.1 |
|  |  | 1 | 19 | 2.9 |
|  | 30 | 0 | 643 | 97.1 |
|  |  | 1 | 19 | 2.9 |
|  | 31 | 0 | 540 | 81.6 |
|  |  | 1 | 122 | 18.4 |
|  | 32 | 0 | 642 | 97.0 |
|  |  | 1 | 20 | 3.0 |
|  | 33 | 0 | 526 | 79.5 |
|  |  | 1 | 136 | 20.5 |
|  | 34 | 0 | 523 | 79.0 |
|  |  | 1 | 139 | 21.0 |
|  | 35 | 0 | 588 | 88.8 |
|  |  | 1 | 74 | 11.2 |
|  | 36 | 0 | 443 | 66.9 |
|  |  | 1 | 219 | 33.1 |
|  | 37 | 0 | 538 | 81.3 |
|  |  | 1 | 124 | 18.7 |
|  | 38 | 0 | 580 | 87.6 |
|  |  | 1 | 82 | 12.4 |
|  | 39 | 0 | 625 | 94.4 |
|  |  | 1 | 37 | 5.6 |
|  | 40 | 0 | 463 | 69.9 |
|  |  | 1 | 199 | 30.1 |
|  | 41 | 0 | 627 | 94.7 |
|  |  | 1 | 35 | 5.3 |
|  | 42 | 0 | 508 | 76.7 |
|  |  | 1 | 154 | 23.3 |
|  | 43 | 0 | 640 | 96.7 |
|  |  | 1 | 22 | 3.3 |
|  | 44 | 0 | 480 | 72.5 |
|  |  | 1 | 182 | 27.5 |
|  | 45 | 0 | 527 | 79.6 |
|  |  | 1 | 135 | 20.4 |
|  | 46 | 0 | 463 | 69.9 |
|  |  | 1 | 199 | 30.1 |
|  | 47 | 0 | 456 | 68.9 |
|  |  | 1 | 206 | 31.1 |
|  | 48 | 0 | 567 | 85.6 |
|  |  | 1 | 95 | 14.4 |
|  | 49 | 0 | 511 | 77.2 |
|  |  | 1 | 151 | 22.8 |
|  | 50 | 0 | 609 | 92.0 |
|  |  | 1 | 53 | 8.0 |

| **Comorbidity_HIV** | | | | |
| --- | --- | --- | --- | --- |
| Data | Imputation | Category | N | Percent |
| Original Data |  | 0 | 490 | 93.0 |
|  |  | 1 | 37 | 7.0 |
| Imputed Values | 1 | 0 | 125 | 92.6 |
|  |  | 1 | 10 | 7.4 |
|  | 2 | 0 | 118 | 87.4 |
|  |  | 1 | 17 | 12.6 |
|  | 3 | 0 | 107 | 79.3 |
|  |  | 1 | 28 | 20.7 |
|  | 4 | 0 | 113 | 83.7 |
|  |  | 1 | 22 | 16.3 |
|  | 5 | 0 | 127 | 94.1 |
|  |  | 1 | 8 | 5.9 |
|  | 6 | 0 | 121 | 89.6 |
|  |  | 1 | 14 | 10.4 |
|  | 7 | 0 | 121 | 89.6 |
|  |  | 1 | 14 | 10.4 |
|  | 8 | 0 | 116 | 85.9 |
|  |  | 1 | 19 | 14.1 |
|  | 9 | 0 | 104 | 77.0 |
|  |  | 1 | 31 | 23.0 |
|  | 10 | 0 | 128 | 94.8 |
|  |  | 1 | 7 | 5.2 |
|  | 11 | 0 | 122 | 90.4 |
|  |  | 1 | 13 | 9.6 |
|  | 12 | 0 | 106 | 78.5 |
|  |  | 1 | 29 | 21.5 |
|  | 13 | 0 | 115 | 85.2 |
|  |  | 1 | 20 | 14.8 |
|  | 14 | 0 | 103 | 76.3 |
|  |  | 1 | 32 | 23.7 |
|  | 15 | 0 | 104 | 77.0 |
|  |  | 1 | 31 | 23.0 |
|  | 16 | 0 | 125 | 92.6 |
|  |  | 1 | 10 | 7.4 |
|  | 17 | 0 | 118 | 87.4 |
|  |  | 1 | 17 | 12.6 |
|  | 18 | 0 | 112 | 83.0 |
|  |  | 1 | 23 | 17.0 |
|  | 19 | 0 | 128 | 94.8 |
|  |  | 1 | 7 | 5.2 |
|  | 20 | 0 | 123 | 91.1 |
|  |  | 1 | 12 | 8.9 |
|  | 21 | 0 | 125 | 92.6 |
|  |  | 1 | 10 | 7.4 |
|  | 22 | 0 | 121 | 89.6 |
|  |  | 1 | 14 | 10.4 |
|  | 23 | 0 | 122 | 90.4 |
|  |  | 1 | 13 | 9.6 |
|  | 24 | 0 | 129 | 95.6 |
|  |  | 1 | 6 | 4.4 |
|  | 25 | 0 | 119 | 88.1 |
|  |  | 1 | 16 | 11.9 |
|  | 26 | 0 | 104 | 77.0 |
|  |  | 1 | 31 | 23.0 |
|  | 27 | 0 | 125 | 92.6 |
|  |  | 1 | 10 | 7.4 |
|  | 28 | 0 | 113 | 83.7 |
|  |  | 1 | 22 | 16.3 |
|  | 29 | 0 | 125 | 92.6 |
|  |  | 1 | 10 | 7.4 |
|  | 30 | 0 | 114 | 84.4 |
|  |  | 1 | 21 | 15.6 |
|  | 31 | 0 | 117 | 86.7 |
|  |  | 1 | 18 | 13.3 |
|  | 32 | 0 | 125 | 92.6 |
|  |  | 1 | 10 | 7.4 |
|  | 33 | 0 | 121 | 89.6 |
|  |  | 1 | 14 | 10.4 |
|  | 34 | 0 | 120 | 88.9 |
|  |  | 1 | 15 | 11.1 |
|  | 35 | 0 | 119 | 88.1 |
|  |  | 1 | 16 | 11.9 |
|  | 36 | 0 | 130 | 96.3 |
|  |  | 1 | 5 | 3.7 |
|  | 37 | 0 | 122 | 90.4 |
|  |  | 1 | 13 | 9.6 |
|  | 38 | 0 | 129 | 95.6 |
|  |  | 1 | 6 | 4.4 |
|  | 39 | 0 | 88 | 65.2 |
|  |  | 1 | 47 | 34.8 |
|  | 40 | 0 | 122 | 90.4 |
|  |  | 1 | 13 | 9.6 |
|  | 41 | 0 | 89 | 65.9 |
|  |  | 1 | 46 | 34.1 |
|  | 42 | 0 | 109 | 80.7 |
|  |  | 1 | 26 | 19.3 |
|  | 43 | 0 | 105 | 77.8 |
|  |  | 1 | 30 | 22.2 |
|  | 44 | 0 | 116 | 85.9 |
|  |  | 1 | 19 | 14.1 |
|  | 45 | 0 | 116 | 85.9 |
|  |  | 1 | 19 | 14.1 |
|  | 46 | 0 | 112 | 83.0 |
|  |  | 1 | 23 | 17.0 |
|  | 47 | 0 | 98 | 72.6 |
|  |  | 1 | 37 | 27.4 |
|  | 48 | 0 | 108 | 80.0 |
|  |  | 1 | 27 | 20.0 |
|  | 49 | 0 | 114 | 84.4 |
|  |  | 1 | 21 | 15.6 |
|  | 50 | 0 | 105 | 77.8 |
|  |  | 1 | 30 | 22.2 |
| Complete Data After Imputation | 1 | 0 | 615 | 92.9 |
|  |  | 1 | 47 | 7.1 |
|  | 2 | 0 | 608 | 91.8 |
|  |  | 1 | 54 | 8.2 |
|  | 3 | 0 | 597 | 90.2 |
|  |  | 1 | 65 | 9.8 |
|  | 4 | 0 | 603 | 91.1 |
|  |  | 1 | 59 | 8.9 |
|  | 5 | 0 | 617 | 93.2 |
|  |  | 1 | 45 | 6.8 |
|  | 6 | 0 | 611 | 92.3 |
|  |  | 1 | 51 | 7.7 |
|  | 7 | 0 | 611 | 92.3 |
|  |  | 1 | 51 | 7.7 |
|  | 8 | 0 | 606 | 91.5 |
|  |  | 1 | 56 | 8.5 |
|  | 9 | 0 | 594 | 89.7 |
|  |  | 1 | 68 | 10.3 |
|  | 10 | 0 | 618 | 93.4 |
|  |  | 1 | 44 | 6.6 |
|  | 11 | 0 | 612 | 92.4 |
|  |  | 1 | 50 | 7.6 |
|  | 12 | 0 | 596 | 90.0 |
|  |  | 1 | 66 | 10.0 |
|  | 13 | 0 | 605 | 91.4 |
|  |  | 1 | 57 | 8.6 |
|  | 14 | 0 | 593 | 89.6 |
|  |  | 1 | 69 | 10.4 |
|  | 15 | 0 | 594 | 89.7 |
|  |  | 1 | 68 | 10.3 |
|  | 16 | 0 | 615 | 92.9 |
|  |  | 1 | 47 | 7.1 |
|  | 17 | 0 | 608 | 91.8 |
|  |  | 1 | 54 | 8.2 |
|  | 18 | 0 | 602 | 90.9 |
|  |  | 1 | 60 | 9.1 |
|  | 19 | 0 | 618 | 93.4 |
|  |  | 1 | 44 | 6.6 |
|  | 20 | 0 | 613 | 92.6 |
|  |  | 1 | 49 | 7.4 |
|  | 21 | 0 | 615 | 92.9 |
|  |  | 1 | 47 | 7.1 |
|  | 22 | 0 | 611 | 92.3 |
|  |  | 1 | 51 | 7.7 |
|  | 23 | 0 | 612 | 92.4 |
|  |  | 1 | 50 | 7.6 |
|  | 24 | 0 | 619 | 93.5 |
|  |  | 1 | 43 | 6.5 |
|  | 25 | 0 | 609 | 92.0 |
|  |  | 1 | 53 | 8.0 |
|  | 26 | 0 | 594 | 89.7 |
|  |  | 1 | 68 | 10.3 |
|  | 27 | 0 | 615 | 92.9 |
|  |  | 1 | 47 | 7.1 |
|  | 28 | 0 | 603 | 91.1 |
|  |  | 1 | 59 | 8.9 |
|  | 29 | 0 | 615 | 92.9 |
|  |  | 1 | 47 | 7.1 |
|  | 30 | 0 | 604 | 91.2 |
|  |  | 1 | 58 | 8.8 |
|  | 31 | 0 | 607 | 91.7 |
|  |  | 1 | 55 | 8.3 |
|  | 32 | 0 | 615 | 92.9 |
|  |  | 1 | 47 | 7.1 |
|  | 33 | 0 | 611 | 92.3 |
|  |  | 1 | 51 | 7.7 |
|  | 34 | 0 | 610 | 92.1 |
|  |  | 1 | 52 | 7.9 |
|  | 35 | 0 | 609 | 92.0 |
|  |  | 1 | 53 | 8.0 |
|  | 36 | 0 | 620 | 93.7 |
|  |  | 1 | 42 | 6.3 |
|  | 37 | 0 | 612 | 92.4 |
|  |  | 1 | 50 | 7.6 |
|  | 38 | 0 | 619 | 93.5 |
|  |  | 1 | 43 | 6.5 |
|  | 39 | 0 | 578 | 87.3 |
|  |  | 1 | 84 | 12.7 |
|  | 40 | 0 | 612 | 92.4 |
|  |  | 1 | 50 | 7.6 |
|  | 41 | 0 | 579 | 87.5 |
|  |  | 1 | 83 | 12.5 |
|  | 42 | 0 | 599 | 90.5 |
|  |  | 1 | 63 | 9.5 |
|  | 43 | 0 | 595 | 89.9 |
|  |  | 1 | 67 | 10.1 |
|  | 44 | 0 | 606 | 91.5 |
|  |  | 1 | 56 | 8.5 |
|  | 45 | 0 | 606 | 91.5 |
|  |  | 1 | 56 | 8.5 |
|  | 46 | 0 | 602 | 90.9 |
|  |  | 1 | 60 | 9.1 |
|  | 47 | 0 | 588 | 88.8 |
|  |  | 1 | 74 | 11.2 |
|  | 48 | 0 | 598 | 90.3 |
|  |  | 1 | 64 | 9.7 |
|  | 49 | 0 | 604 | 91.2 |
|  |  | 1 | 58 | 8.8 |
|  | 50 | 0 | 595 | 89.9 |
|  |  | 1 | 67 | 10.1 |

| **Comorbidity_respiratory_disorder** | | | | |
| --- | --- | --- | --- | --- |
| Data | Imputation | Category | N | Percent |
| Original Data |  | 0 | 404 | 88.2 |
|  |  | 1 | 54 | 11.8 |
| Imputed Values | 1 | 0 | 171 | 83.8 |
|  |  | 1 | 33 | 16.2 |
|  | 2 | 0 | 57 | 27.9 |
|  |  | 1 | 147 | 72.1 |
|  | 3 | 0 | 149 | 73.0 |
|  |  | 1 | 55 | 27.0 |
|  | 4 | 0 | 185 | 90.7 |
|  |  | 1 | 19 | 9.3 |
|  | 5 | 0 | 188 | 92.2 |
|  |  | 1 | 16 | 7.8 |
|  | 6 | 0 | 124 | 60.8 |
|  |  | 1 | 80 | 39.2 |
|  | 7 | 0 | 162 | 79.4 |
|  |  | 1 | 42 | 20.6 |
|  | 8 | 0 | 170 | 83.3 |
|  |  | 1 | 34 | 16.7 |
|  | 9 | 0 | 167 | 81.9 |
|  |  | 1 | 37 | 18.1 |
|  | 10 | 0 | 71 | 34.8 |
|  |  | 1 | 133 | 65.2 |
|  | 11 | 0 | 190 | 93.1 |
|  |  | 1 | 14 | 6.9 |
|  | 12 | 0 | 182 | 89.2 |
|  |  | 1 | 22 | 10.8 |
|  | 13 | 0 | 201 | 98.5 |
|  |  | 1 | 3 | 1.5 |
|  | 14 | 0 | 180 | 88.2 |
|  |  | 1 | 24 | 11.8 |
|  | 15 | 0 | 100 | 49.0 |
|  |  | 1 | 104 | 51.0 |
|  | 16 | 0 | 134 | 65.7 |
|  |  | 1 | 70 | 34.3 |
|  | 17 | 0 | 152 | 74.5 |
|  |  | 1 | 52 | 25.5 |
|  | 18 | 0 | 176 | 86.3 |
|  |  | 1 | 28 | 13.7 |
|  | 19 | 0 | 182 | 89.2 |
|  |  | 1 | 22 | 10.8 |
|  | 20 | 0 | 160 | 78.4 |
|  |  | 1 | 44 | 21.6 |
|  | 21 | 0 | 172 | 84.3 |
|  |  | 1 | 32 | 15.7 |
|  | 22 | 0 | 173 | 84.8 |
|  |  | 1 | 31 | 15.2 |
|  | 23 | 0 | 176 | 86.3 |
|  |  | 1 | 28 | 13.7 |
|  | 24 | 0 | 152 | 74.5 |
|  |  | 1 | 52 | 25.5 |
|  | 25 | 0 | 83 | 40.7 |
|  |  | 1 | 121 | 59.3 |
|  | 26 | 0 | 170 | 83.3 |
|  |  | 1 | 34 | 16.7 |
|  | 27 | 0 | 128 | 62.7 |
|  |  | 1 | 76 | 37.3 |
|  | 28 | 0 | 131 | 64.2 |
|  |  | 1 | 73 | 35.8 |
|  | 29 | 0 | 152 | 74.5 |
|  |  | 1 | 52 | 25.5 |
|  | 30 | 0 | 32 | 15.7 |
|  |  | 1 | 172 | 84.3 |
|  | 31 | 0 | 181 | 88.7 |
|  |  | 1 | 23 | 11.3 |
|  | 32 | 0 | 108 | 52.9 |
|  |  | 1 | 96 | 47.1 |
|  | 33 | 0 | 177 | 86.8 |
|  |  | 1 | 27 | 13.2 |
|  | 34 | 0 | 116 | 56.9 |
|  |  | 1 | 88 | 43.1 |
|  | 35 | 0 | 167 | 81.9 |
|  |  | 1 | 37 | 18.1 |
|  | 36 | 0 | 147 | 72.1 |
|  |  | 1 | 57 | 27.9 |
|  | 37 | 0 | 78 | 38.2 |
|  |  | 1 | 126 | 61.8 |
|  | 38 | 0 | 145 | 71.1 |
|  |  | 1 | 59 | 28.9 |
|  | 39 | 0 | 34 | 16.7 |
|  |  | 1 | 170 | 83.3 |
|  | 40 | 0 | 132 | 64.7 |
|  |  | 1 | 72 | 35.3 |
|  | 41 | 0 | 187 | 91.7 |
|  |  | 1 | 17 | 8.3 |
|  | 42 | 0 | 85 | 41.7 |
|  |  | 1 | 119 | 58.3 |
|  | 43 | 0 | 179 | 87.7 |
|  |  | 1 | 25 | 12.3 |
|  | 44 | 0 | 31 | 15.2 |
|  |  | 1 | 173 | 84.8 |
|  | 45 | 0 | 173 | 84.8 |
|  |  | 1 | 31 | 15.2 |
|  | 46 | 0 | 110 | 53.9 |
|  |  | 1 | 94 | 46.1 |
|  | 47 | 0 | 176 | 86.3 |
|  |  | 1 | 28 | 13.7 |
|  | 48 | 0 | 149 | 73.0 |
|  |  | 1 | 55 | 27.0 |
|  | 49 | 0 | 91 | 44.6 |
|  |  | 1 | 113 | 55.4 |
|  | 50 | 0 | 106 | 52.0 |
|  |  | 1 | 98 | 48.0 |
| Complete Data After Imputation | 1 | 0 | 575 | 86.9 |
|  |  | 1 | 87 | 13.1 |
|  | 2 | 0 | 461 | 69.6 |
|  |  | 1 | 201 | 30.4 |
|  | 3 | 0 | 553 | 83.5 |
|  |  | 1 | 109 | 16.5 |
|  | 4 | 0 | 589 | 89.0 |
|  |  | 1 | 73 | 11.0 |
|  | 5 | 0 | 592 | 89.4 |
|  |  | 1 | 70 | 10.6 |
|  | 6 | 0 | 528 | 79.8 |
|  |  | 1 | 134 | 20.2 |
|  | 7 | 0 | 566 | 85.5 |
|  |  | 1 | 96 | 14.5 |
|  | 8 | 0 | 574 | 86.7 |
|  |  | 1 | 88 | 13.3 |
|  | 9 | 0 | 571 | 86.3 |
|  |  | 1 | 91 | 13.7 |
|  | 10 | 0 | 475 | 71.8 |
|  |  | 1 | 187 | 28.2 |
|  | 11 | 0 | 594 | 89.7 |
|  |  | 1 | 68 | 10.3 |
|  | 12 | 0 | 586 | 88.5 |
|  |  | 1 | 76 | 11.5 |
|  | 13 | 0 | 605 | 91.4 |
|  |  | 1 | 57 | 8.6 |
|  | 14 | 0 | 584 | 88.2 |
|  |  | 1 | 78 | 11.8 |
|  | 15 | 0 | 504 | 76.1 |
|  |  | 1 | 158 | 23.9 |
|  | 16 | 0 | 538 | 81.3 |
|  |  | 1 | 124 | 18.7 |
|  | 17 | 0 | 556 | 84.0 |
|  |  | 1 | 106 | 16.0 |
|  | 18 | 0 | 580 | 87.6 |
|  |  | 1 | 82 | 12.4 |
|  | 19 | 0 | 586 | 88.5 |
|  |  | 1 | 76 | 11.5 |
|  | 20 | 0 | 564 | 85.2 |
|  |  | 1 | 98 | 14.8 |
|  | 21 | 0 | 576 | 87.0 |
|  |  | 1 | 86 | 13.0 |
|  | 22 | 0 | 577 | 87.2 |
|  |  | 1 | 85 | 12.8 |
|  | 23 | 0 | 580 | 87.6 |
|  |  | 1 | 82 | 12.4 |
|  | 24 | 0 | 556 | 84.0 |
|  |  | 1 | 106 | 16.0 |
|  | 25 | 0 | 487 | 73.6 |
|  |  | 1 | 175 | 26.4 |
|  | 26 | 0 | 574 | 86.7 |
|  |  | 1 | 88 | 13.3 |
|  | 27 | 0 | 532 | 80.4 |
|  |  | 1 | 130 | 19.6 |
|  | 28 | 0 | 535 | 80.8 |
|  |  | 1 | 127 | 19.2 |
|  | 29 | 0 | 556 | 84.0 |
|  |  | 1 | 106 | 16.0 |
|  | 30 | 0 | 436 | 65.9 |
|  |  | 1 | 226 | 34.1 |
|  | 31 | 0 | 585 | 88.4 |
|  |  | 1 | 77 | 11.6 |
|  | 32 | 0 | 512 | 77.3 |
|  |  | 1 | 150 | 22.7 |
|  | 33 | 0 | 581 | 87.8 |
|  |  | 1 | 81 | 12.2 |
|  | 34 | 0 | 520 | 78.5 |
|  |  | 1 | 142 | 21.5 |
|  | 35 | 0 | 571 | 86.3 |
|  |  | 1 | 91 | 13.7 |
|  | 36 | 0 | 551 | 83.2 |
|  |  | 1 | 111 | 16.8 |
|  | 37 | 0 | 482 | 72.8 |
|  |  | 1 | 180 | 27.2 |
|  | 38 | 0 | 549 | 82.9 |
|  |  | 1 | 113 | 17.1 |
|  | 39 | 0 | 438 | 66.2 |
|  |  | 1 | 224 | 33.8 |
|  | 40 | 0 | 536 | 81.0 |
|  |  | 1 | 126 | 19.0 |
|  | 41 | 0 | 591 | 89.3 |
|  |  | 1 | 71 | 10.7 |
|  | 42 | 0 | 489 | 73.9 |
|  |  | 1 | 173 | 26.1 |
|  | 43 | 0 | 583 | 88.1 |
|  |  | 1 | 79 | 11.9 |
|  | 44 | 0 | 435 | 65.7 |
|  |  | 1 | 227 | 34.3 |
|  | 45 | 0 | 577 | 87.2 |
|  |  | 1 | 85 | 12.8 |
|  | 46 | 0 | 514 | 77.6 |
|  |  | 1 | 148 | 22.4 |
|  | 47 | 0 | 580 | 87.6 |
|  |  | 1 | 82 | 12.4 |
|  | 48 | 0 | 553 | 83.5 |
|  |  | 1 | 109 | 16.5 |
|  | 49 | 0 | 495 | 74.8 |
|  |  | 1 | 167 | 25.2 |
|  | 50 | 0 | 510 | 77.0 |
|  |  | 1 | 152 | 23.0 |

| **Comorbidity_drugs_alcohol** | | | | |
| --- | --- | --- | --- | --- |
| Data | Imputation | Category | N | Percent |
| Original Data |  | 0 | 440 | 96.1 |
|  |  | 1 | 18 | 3.9 |
| Imputed Values | 1 | 0 | 204 | 100.0 |
|  | 2 | 0 | 160 | 78.4 |
|  |  | 1 | 44 | 21.6 |
|  | 3 | 0 | 204 | 100.0 |
|  | 4 | 0 | 127 | 62.3 |
|  |  | 1 | 77 | 37.7 |
|  | 5 | 0 | 66 | 32.4 |
|  |  | 1 | 138 | 67.6 |
|  | 6 | 0 | 79 | 38.7 |
|  |  | 1 | 125 | 61.3 |
|  | 7 | 0 | 151 | 74.0 |
|  |  | 1 | 53 | 26.0 |
|  | 8 | 0 | 204 | 100.0 |
|  | 9 | 0 | 204 | 100.0 |
|  | 10 | 0 | 71 | 34.8 |
|  |  | 1 | 133 | 65.2 |
|  | 11 | 0 | 3 | 1.5 |
|  |  | 1 | 201 | 98.5 |
|  | 12 | 0 | 109 | 53.4 |
|  |  | 1 | 95 | 46.6 |
|  | 13 | 0 | 97 | 47.5 |
|  |  | 1 | 107 | 52.5 |
|  | 14 | 0 | 17 | 8.3 |
|  |  | 1 | 187 | 91.7 |
|  | 15 | 0 | 196 | 96.1 |
|  |  | 1 | 8 | 3.9 |
|  | 16 | 0 | 14 | 6.9 |
|  |  | 1 | 190 | 93.1 |
|  | 17 | 0 | 172 | 84.3 |
|  |  | 1 | 32 | 15.7 |
|  | 18 | 0 | 2 | 1.0 |
|  |  | 1 | 202 | 99.0 |
|  | 19 | 0 | 11 | 5.4 |
|  |  | 1 | 193 | 94.6 |
|  | 20 | 0 | 154 | 75.5 |
|  |  | 1 | 50 | 24.5 |
|  | 21 | 0 | 66 | 32.4 |
|  |  | 1 | 138 | 67.6 |
|  | 22 | 0 | 21 | 10.3 |
|  |  | 1 | 183 | 89.7 |
|  | 23 | 0 | 167 | 81.9 |
|  |  | 1 | 37 | 18.1 |
|  | 24 | 0 | 204 | 100.0 |
|  | 25 | 0 | 157 | 77.0 |
|  |  | 1 | 47 | 23.0 |
|  | 26 | 0 | 203 | 99.5 |
|  |  | 1 | 1 | .5 |
|  | 27 | 0 | 59 | 28.9 |
|  |  | 1 | 145 | 71.1 |
|  | 28 | 0 | 8 | 3.9 |
|  |  | 1 | 196 | 96.1 |
|  | 29 | 0 | 19 | 9.3 |
|  |  | 1 | 185 | 90.7 |
|  | 30 | 0 | 179 | 87.7 |
|  |  | 1 | 25 | 12.3 |
|  | 31 | 0 | 3 | 1.5 |
|  |  | 1 | 201 | 98.5 |
|  | 32 | 0 | 43 | 21.1 |
|  |  | 1 | 161 | 78.9 |
|  | 33 | 0 | 110 | 53.9 |
|  |  | 1 | 94 | 46.1 |
|  | 34 | 0 | 66 | 32.4 |
|  |  | 1 | 138 | 67.6 |
|  | 35 | 0 | 86 | 42.2 |
|  |  | 1 | 118 | 57.8 |
|  | 36 | 0 | 194 | 95.1 |
|  |  | 1 | 10 | 4.9 |
|  | 37 | 0 | 199 | 97.5 |
|  |  | 1 | 5 | 2.5 |
|  | 38 | 0 | 56 | 27.5 |
|  |  | 1 | 148 | 72.5 |
|  | 39 | 1 | 204 | 100.0 |
|  | 40 | 0 | 153 | 75.0 |
|  |  | 1 | 51 | 25.0 |
|  | 41 | 0 | 12 | 5.9 |
|  |  | 1 | 192 | 94.1 |
|  | 42 | 0 | 203 | 99.5 |
|  |  | 1 | 1 | .5 |
|  | 43 | 0 | 161 | 78.9 |
|  |  | 1 | 43 | 21.1 |
|  | 44 | 0 | 19 | 9.3 |
|  |  | 1 | 185 | 90.7 |
|  | 45 | 0 | 65 | 31.9 |
|  |  | 1 | 139 | 68.1 |
|  | 46 | 0 | 10 | 4.9 |
|  |  | 1 | 194 | 95.1 |
|  | 47 | 0 | 190 | 93.1 |
|  |  | 1 | 14 | 6.9 |
|  | 48 | 0 | 150 | 73.5 |
|  |  | 1 | 54 | 26.5 |
|  | 49 | 0 | 176 | 86.3 |
|  |  | 1 | 28 | 13.7 |
|  | 50 | 0 | 172 | 84.3 |
|  |  | 1 | 32 | 15.7 |
| Complete Data After Imputation | 1 | 0 | 644 | 97.3 |
|  |  | 1 | 18 | 2.7 |
|  | 2 | 0 | 600 | 90.6 |
|  |  | 1 | 62 | 9.4 |
|  | 3 | 0 | 644 | 97.3 |
|  |  | 1 | 18 | 2.7 |
|  | 4 | 0 | 567 | 85.6 |
|  |  | 1 | 95 | 14.4 |
|  | 5 | 0 | 506 | 76.4 |
|  |  | 1 | 156 | 23.6 |
|  | 6 | 0 | 519 | 78.4 |
|  |  | 1 | 143 | 21.6 |
|  | 7 | 0 | 591 | 89.3 |
|  |  | 1 | 71 | 10.7 |
|  | 8 | 0 | 644 | 97.3 |
|  |  | 1 | 18 | 2.7 |
|  | 9 | 0 | 644 | 97.3 |
|  |  | 1 | 18 | 2.7 |
|  | 10 | 0 | 511 | 77.2 |
|  |  | 1 | 151 | 22.8 |
|  | 11 | 0 | 443 | 66.9 |
|  |  | 1 | 219 | 33.1 |
|  | 12 | 0 | 549 | 82.9 |
|  |  | 1 | 113 | 17.1 |
|  | 13 | 0 | 537 | 81.1 |
|  |  | 1 | 125 | 18.9 |
|  | 14 | 0 | 457 | 69.0 |
|  |  | 1 | 205 | 31.0 |
|  | 15 | 0 | 636 | 96.1 |
|  |  | 1 | 26 | 3.9 |
|  | 16 | 0 | 454 | 68.6 |
|  |  | 1 | 208 | 31.4 |
|  | 17 | 0 | 612 | 92.4 |
|  |  | 1 | 50 | 7.6 |
|  | 18 | 0 | 442 | 66.8 |
|  |  | 1 | 220 | 33.2 |
|  | 19 | 0 | 451 | 68.1 |
|  |  | 1 | 211 | 31.9 |
|  | 20 | 0 | 594 | 89.7 |
|  |  | 1 | 68 | 10.3 |
|  | 21 | 0 | 506 | 76.4 |
|  |  | 1 | 156 | 23.6 |
|  | 22 | 0 | 461 | 69.6 |
|  |  | 1 | 201 | 30.4 |
|  | 23 | 0 | 607 | 91.7 |
|  |  | 1 | 55 | 8.3 |
|  | 24 | 0 | 644 | 97.3 |
|  |  | 1 | 18 | 2.7 |
|  | 25 | 0 | 597 | 90.2 |
|  |  | 1 | 65 | 9.8 |
|  | 26 | 0 | 643 | 97.1 |
|  |  | 1 | 19 | 2.9 |
|  | 27 | 0 | 499 | 75.4 |
|  |  | 1 | 163 | 24.6 |
|  | 28 | 0 | 448 | 67.7 |
|  |  | 1 | 214 | 32.3 |
|  | 29 | 0 | 459 | 69.3 |
|  |  | 1 | 203 | 30.7 |
|  | 30 | 0 | 619 | 93.5 |
|  |  | 1 | 43 | 6.5 |
|  | 31 | 0 | 443 | 66.9 |
|  |  | 1 | 219 | 33.1 |
|  | 32 | 0 | 483 | 73.0 |
|  |  | 1 | 179 | 27.0 |
|  | 33 | 0 | 550 | 83.1 |
|  |  | 1 | 112 | 16.9 |
|  | 34 | 0 | 506 | 76.4 |
|  |  | 1 | 156 | 23.6 |
|  | 35 | 0 | 526 | 79.5 |
|  |  | 1 | 136 | 20.5 |
|  | 36 | 0 | 634 | 95.8 |
|  |  | 1 | 28 | 4.2 |
|  | 37 | 0 | 639 | 96.5 |
|  |  | 1 | 23 | 3.5 |
|  | 38 | 0 | 496 | 74.9 |
|  |  | 1 | 166 | 25.1 |
|  | 39 | 0 | 440 | 66.5 |
|  |  | 1 | 222 | 33.5 |
|  | 40 | 0 | 593 | 89.6 |
|  |  | 1 | 69 | 10.4 |
|  | 41 | 0 | 452 | 68.3 |
|  |  | 1 | 210 | 31.7 |
|  | 42 | 0 | 643 | 97.1 |
|  |  | 1 | 19 | 2.9 |
|  | 43 | 0 | 601 | 90.8 |
|  |  | 1 | 61 | 9.2 |
|  | 44 | 0 | 459 | 69.3 |
|  |  | 1 | 203 | 30.7 |
|  | 45 | 0 | 505 | 76.3 |
|  |  | 1 | 157 | 23.7 |
|  | 46 | 0 | 450 | 68.0 |
|  |  | 1 | 212 | 32.0 |
|  | 47 | 0 | 630 | 95.2 |
|  |  | 1 | 32 | 4.8 |
|  | 48 | 0 | 590 | 89.1 |
|  |  | 1 | 72 | 10.9 |
|  | 49 | 0 | 616 | 93.1 |
|  |  | 1 | 46 | 6.9 |
|  | 50 | 0 | 612 | 92.4 |
|  |  | 1 | 50 | 7.6 |

| **RR_MDRorPreXDR_XDR** | | | | |
| --- | --- | --- | --- | --- |
| Data | Imputation | Category | N | Percent |
| Original Data |  | 0 | 388 | 88.0 |
|  |  | 1 | 53 | 12.0 |
| Imputed Values | 1 | 0 | 128 | 57.9 |
|  |  | 1 | 93 | 42.1 |
|  | 2 | 0 | 111 | 50.2 |
|  |  | 1 | 110 | 49.8 |
|  | 3 | 0 | 154 | 69.7 |
|  |  | 1 | 67 | 30.3 |
|  | 4 | 0 | 124 | 56.1 |
|  |  | 1 | 97 | 43.9 |
|  | 5 | 0 | 173 | 78.3 |
|  |  | 1 | 48 | 21.7 |
|  | 6 | 0 | 124 | 56.1 |
|  |  | 1 | 97 | 43.9 |
|  | 7 | 0 | 105 | 47.5 |
|  |  | 1 | 116 | 52.5 |
|  | 8 | 0 | 150 | 67.9 |
|  |  | 1 | 71 | 32.1 |
|  | 9 | 0 | 126 | 57.0 |
|  |  | 1 | 95 | 43.0 |
|  | 10 | 0 | 153 | 69.2 |
|  |  | 1 | 68 | 30.8 |
|  | 11 | 0 | 157 | 71.0 |
|  |  | 1 | 64 | 29.0 |
|  | 12 | 0 | 120 | 54.3 |
|  |  | 1 | 101 | 45.7 |
|  | 13 | 0 | 165 | 74.7 |
|  |  | 1 | 56 | 25.3 |
|  | 14 | 0 | 161 | 72.9 |
|  |  | 1 | 60 | 27.1 |
|  | 15 | 0 | 127 | 57.5 |
|  |  | 1 | 94 | 42.5 |
|  | 16 | 0 | 157 | 71.0 |
|  |  | 1 | 64 | 29.0 |
|  | 17 | 0 | 137 | 62.0 |
|  |  | 1 | 84 | 38.0 |
|  | 18 | 0 | 126 | 57.0 |
|  |  | 1 | 95 | 43.0 |
|  | 19 | 0 | 143 | 64.7 |
|  |  | 1 | 78 | 35.3 |
|  | 20 | 0 | 123 | 55.7 |
|  |  | 1 | 98 | 44.3 |
|  | 21 | 0 | 145 | 65.6 |
|  |  | 1 | 76 | 34.4 |
|  | 22 | 0 | 118 | 53.4 |
|  |  | 1 | 103 | 46.6 |
|  | 23 | 0 | 156 | 70.6 |
|  |  | 1 | 65 | 29.4 |
|  | 24 | 0 | 155 | 70.1 |
|  |  | 1 | 66 | 29.9 |
|  | 25 | 0 | 152 | 68.8 |
|  |  | 1 | 69 | 31.2 |
|  | 26 | 0 | 139 | 62.9 |
|  |  | 1 | 82 | 37.1 |
|  | 27 | 0 | 181 | 81.9 |
|  |  | 1 | 40 | 18.1 |
|  | 28 | 0 | 150 | 67.9 |
|  |  | 1 | 71 | 32.1 |
|  | 29 | 0 | 125 | 56.6 |
|  |  | 1 | 96 | 43.4 |
|  | 30 | 0 | 172 | 77.8 |
|  |  | 1 | 49 | 22.2 |
|  | 31 | 0 | 158 | 71.5 |
|  |  | 1 | 63 | 28.5 |
|  | 32 | 0 | 129 | 58.4 |
|  |  | 1 | 92 | 41.6 |
|  | 33 | 0 | 114 | 51.6 |
|  |  | 1 | 107 | 48.4 |
|  | 34 | 0 | 167 | 75.6 |
|  |  | 1 | 54 | 24.4 |
|  | 35 | 0 | 155 | 70.1 |
|  |  | 1 | 66 | 29.9 |
|  | 36 | 0 | 131 | 59.3 |
|  |  | 1 | 90 | 40.7 |
|  | 37 | 0 | 141 | 63.8 |
|  |  | 1 | 80 | 36.2 |
|  | 38 | 0 | 123 | 55.7 |
|  |  | 1 | 98 | 44.3 |
|  | 39 | 0 | 132 | 59.7 |
|  |  | 1 | 89 | 40.3 |
|  | 40 | 0 | 138 | 62.4 |
|  |  | 1 | 83 | 37.6 |
|  | 41 | 0 | 120 | 54.3 |
|  |  | 1 | 101 | 45.7 |
|  | 42 | 0 | 121 | 54.8 |
|  |  | 1 | 100 | 45.2 |
|  | 43 | 0 | 122 | 55.2 |
|  |  | 1 | 99 | 44.8 |
|  | 44 | 0 | 130 | 58.8 |
|  |  | 1 | 91 | 41.2 |
|  | 45 | 0 | 158 | 71.5 |
|  |  | 1 | 63 | 28.5 |
|  | 46 | 0 | 128 | 57.9 |
|  |  | 1 | 93 | 42.1 |
|  | 47 | 0 | 147 | 66.5 |
|  |  | 1 | 74 | 33.5 |
|  | 48 | 0 | 145 | 65.6 |
|  |  | 1 | 76 | 34.4 |
|  | 49 | 0 | 131 | 59.3 |
|  |  | 1 | 90 | 40.7 |
|  | 50 | 0 | 167 | 75.6 |
|  |  | 1 | 54 | 24.4 |
| Complete Data After Imputation | 1 | 0 | 516 | 77.9 |
|  |  | 1 | 146 | 22.1 |
|  | 2 | 0 | 499 | 75.4 |
|  |  | 1 | 163 | 24.6 |
|  | 3 | 0 | 542 | 81.9 |
|  |  | 1 | 120 | 18.1 |
|  | 4 | 0 | 512 | 77.3 |
|  |  | 1 | 150 | 22.7 |
|  | 5 | 0 | 561 | 84.7 |
|  |  | 1 | 101 | 15.3 |
|  | 6 | 0 | 512 | 77.3 |
|  |  | 1 | 150 | 22.7 |
|  | 7 | 0 | 493 | 74.5 |
|  |  | 1 | 169 | 25.5 |
|  | 8 | 0 | 538 | 81.3 |
|  |  | 1 | 124 | 18.7 |
|  | 9 | 0 | 514 | 77.6 |
|  |  | 1 | 148 | 22.4 |
|  | 10 | 0 | 541 | 81.7 |
|  |  | 1 | 121 | 18.3 |
|  | 11 | 0 | 545 | 82.3 |
|  |  | 1 | 117 | 17.7 |
|  | 12 | 0 | 508 | 76.7 |
|  |  | 1 | 154 | 23.3 |
|  | 13 | 0 | 553 | 83.5 |
|  |  | 1 | 109 | 16.5 |
|  | 14 | 0 | 549 | 82.9 |
|  |  | 1 | 113 | 17.1 |
|  | 15 | 0 | 515 | 77.8 |
|  |  | 1 | 147 | 22.2 |
|  | 16 | 0 | 545 | 82.3 |
|  |  | 1 | 117 | 17.7 |
|  | 17 | 0 | 525 | 79.3 |
|  |  | 1 | 137 | 20.7 |
|  | 18 | 0 | 514 | 77.6 |
|  |  | 1 | 148 | 22.4 |
|  | 19 | 0 | 531 | 80.2 |
|  |  | 1 | 131 | 19.8 |
|  | 20 | 0 | 511 | 77.2 |
|  |  | 1 | 151 | 22.8 |
|  | 21 | 0 | 533 | 80.5 |
|  |  | 1 | 129 | 19.5 |
|  | 22 | 0 | 506 | 76.4 |
|  |  | 1 | 156 | 23.6 |
|  | 23 | 0 | 544 | 82.2 |
|  |  | 1 | 118 | 17.8 |
|  | 24 | 0 | 543 | 82.0 |
|  |  | 1 | 119 | 18.0 |
|  | 25 | 0 | 540 | 81.6 |
|  |  | 1 | 122 | 18.4 |
|  | 26 | 0 | 527 | 79.6 |
|  |  | 1 | 135 | 20.4 |
|  | 27 | 0 | 569 | 86.0 |
|  |  | 1 | 93 | 14.0 |
|  | 28 | 0 | 538 | 81.3 |
|  |  | 1 | 124 | 18.7 |
|  | 29 | 0 | 513 | 77.5 |
|  |  | 1 | 149 | 22.5 |
|  | 30 | 0 | 560 | 84.6 |
|  |  | 1 | 102 | 15.4 |
|  | 31 | 0 | 546 | 82.5 |
|  |  | 1 | 116 | 17.5 |
|  | 32 | 0 | 517 | 78.1 |
|  |  | 1 | 145 | 21.9 |
|  | 33 | 0 | 502 | 75.8 |
|  |  | 1 | 160 | 24.2 |
|  | 34 | 0 | 555 | 83.8 |
|  |  | 1 | 107 | 16.2 |
|  | 35 | 0 | 543 | 82.0 |
|  |  | 1 | 119 | 18.0 |
|  | 36 | 0 | 519 | 78.4 |
|  |  | 1 | 143 | 21.6 |
|  | 37 | 0 | 529 | 79.9 |
|  |  | 1 | 133 | 20.1 |
|  | 38 | 0 | 511 | 77.2 |
|  |  | 1 | 151 | 22.8 |
|  | 39 | 0 | 520 | 78.5 |
|  |  | 1 | 142 | 21.5 |
|  | 40 | 0 | 526 | 79.5 |
|  |  | 1 | 136 | 20.5 |
|  | 41 | 0 | 508 | 76.7 |
|  |  | 1 | 154 | 23.3 |
|  | 42 | 0 | 509 | 76.9 |
|  |  | 1 | 153 | 23.1 |
|  | 43 | 0 | 510 | 77.0 |
|  |  | 1 | 152 | 23.0 |
|  | 44 | 0 | 518 | 78.2 |
|  |  | 1 | 144 | 21.8 |
|  | 45 | 0 | 546 | 82.5 |
|  |  | 1 | 116 | 17.5 |
|  | 46 | 0 | 516 | 77.9 |
|  |  | 1 | 146 | 22.1 |
|  | 47 | 0 | 535 | 80.8 |
|  |  | 1 | 127 | 19.2 |
|  | 48 | 0 | 533 | 80.5 |
|  |  | 1 | 129 | 19.5 |
|  | 49 | 0 | 519 | 78.4 |
|  |  | 1 | 143 | 21.6 |
|  | 50 | 0 | 555 | 83.8 |
|  |  | 1 | 107 | 16.2 |

| **Smear_positive** | | | | |
| --- | --- | --- | --- | --- |
| Data | Imputation | Category | N | Percent |
| Original Data |  | 0 | 228 | 36.8 |
|  |  | 1 | 392 | 63.2 |
| Imputed Values | 1 | 0 | 13 | 31.0 |
|  |  | 1 | 29 | 69.0 |
|  | 2 | 0 | 15 | 35.7 |
|  |  | 1 | 27 | 64.3 |
|  | 3 | 0 | 18 | 42.9 |
|  |  | 1 | 24 | 57.1 |
|  | 4 | 0 | 11 | 26.2 |
|  |  | 1 | 31 | 73.8 |
|  | 5 | 0 | 12 | 28.6 |
|  |  | 1 | 30 | 71.4 |
|  | 6 | 0 | 15 | 35.7 |
|  |  | 1 | 27 | 64.3 |
|  | 7 | 0 | 16 | 38.1 |
|  |  | 1 | 26 | 61.9 |
|  | 8 | 0 | 7 | 16.7 |
|  |  | 1 | 35 | 83.3 |
|  | 9 | 0 | 17 | 40.5 |
|  |  | 1 | 25 | 59.5 |
|  | 10 | 0 | 15 | 35.7 |
|  |  | 1 | 27 | 64.3 |
|  | 11 | 0 | 17 | 40.5 |
|  |  | 1 | 25 | 59.5 |
|  | 12 | 0 | 15 | 35.7 |
|  |  | 1 | 27 | 64.3 |
|  | 13 | 0 | 20 | 47.6 |
|  |  | 1 | 22 | 52.4 |
|  | 14 | 0 | 15 | 35.7 |
|  |  | 1 | 27 | 64.3 |
|  | 15 | 0 | 14 | 33.3 |
|  |  | 1 | 28 | 66.7 |
|  | 16 | 0 | 12 | 28.6 |
|  |  | 1 | 30 | 71.4 |
|  | 17 | 0 | 16 | 38.1 |
|  |  | 1 | 26 | 61.9 |
|  | 18 | 0 | 13 | 31.0 |
|  |  | 1 | 29 | 69.0 |
|  | 19 | 0 | 16 | 38.1 |
|  |  | 1 | 26 | 61.9 |
|  | 20 | 0 | 22 | 52.4 |
|  |  | 1 | 20 | 47.6 |
|  | 21 | 0 | 16 | 38.1 |
|  |  | 1 | 26 | 61.9 |
|  | 22 | 0 | 17 | 40.5 |
|  |  | 1 | 25 | 59.5 |
|  | 23 | 0 | 19 | 45.2 |
|  |  | 1 | 23 | 54.8 |
|  | 24 | 0 | 13 | 31.0 |
|  |  | 1 | 29 | 69.0 |
|  | 25 | 0 | 16 | 38.1 |
|  |  | 1 | 26 | 61.9 |
|  | 26 | 0 | 15 | 35.7 |
|  |  | 1 | 27 | 64.3 |
|  | 27 | 0 | 16 | 38.1 |
|  |  | 1 | 26 | 61.9 |
|  | 28 | 0 | 17 | 40.5 |
|  |  | 1 | 25 | 59.5 |
|  | 29 | 0 | 16 | 38.1 |
|  |  | 1 | 26 | 61.9 |
|  | 30 | 0 | 17 | 40.5 |
|  |  | 1 | 25 | 59.5 |
|  | 31 | 0 | 13 | 31.0 |
|  |  | 1 | 29 | 69.0 |
|  | 32 | 0 | 19 | 45.2 |
|  |  | 1 | 23 | 54.8 |
|  | 33 | 0 | 17 | 40.5 |
|  |  | 1 | 25 | 59.5 |
|  | 34 | 0 | 16 | 38.1 |
|  |  | 1 | 26 | 61.9 |
|  | 35 | 0 | 19 | 45.2 |
|  |  | 1 | 23 | 54.8 |
|  | 36 | 0 | 14 | 33.3 |
|  |  | 1 | 28 | 66.7 |
|  | 37 | 0 | 16 | 38.1 |
|  |  | 1 | 26 | 61.9 |
|  | 38 | 0 | 19 | 45.2 |
|  |  | 1 | 23 | 54.8 |
|  | 39 | 0 | 17 | 40.5 |
|  |  | 1 | 25 | 59.5 |
|  | 40 | 0 | 14 | 33.3 |
|  |  | 1 | 28 | 66.7 |
|  | 41 | 0 | 13 | 31.0 |
|  |  | 1 | 29 | 69.0 |
|  | 42 | 0 | 13 | 31.0 |
|  |  | 1 | 29 | 69.0 |
|  | 43 | 0 | 17 | 40.5 |
|  |  | 1 | 25 | 59.5 |
|  | 44 | 0 | 19 | 45.2 |
|  |  | 1 | 23 | 54.8 |
|  | 45 | 0 | 15 | 35.7 |
|  |  | 1 | 27 | 64.3 |
|  | 46 | 0 | 17 | 40.5 |
|  |  | 1 | 25 | 59.5 |
|  | 47 | 0 | 11 | 26.2 |
|  |  | 1 | 31 | 73.8 |
|  | 48 | 0 | 8 | 19.0 |
|  |  | 1 | 34 | 81.0 |
|  | 49 | 0 | 16 | 38.1 |
|  |  | 1 | 26 | 61.9 |
|  | 50 | 0 | 16 | 38.1 |
|  |  | 1 | 26 | 61.9 |
| Complete Data After Imputation | 1 | 0 | 241 | 36.4 |
|  |  | 1 | 421 | 63.6 |
|  | 2 | 0 | 243 | 36.7 |
|  |  | 1 | 419 | 63.3 |
|  | 3 | 0 | 246 | 37.2 |
|  |  | 1 | 416 | 62.8 |
|  | 4 | 0 | 239 | 36.1 |
|  |  | 1 | 423 | 63.9 |
|  | 5 | 0 | 240 | 36.3 |
|  |  | 1 | 422 | 63.7 |
|  | 6 | 0 | 243 | 36.7 |
|  |  | 1 | 419 | 63.3 |
|  | 7 | 0 | 244 | 36.9 |
|  |  | 1 | 418 | 63.1 |
|  | 8 | 0 | 235 | 35.5 |
|  |  | 1 | 427 | 64.5 |
|  | 9 | 0 | 245 | 37.0 |
|  |  | 1 | 417 | 63.0 |
|  | 10 | 0 | 243 | 36.7 |
|  |  | 1 | 419 | 63.3 |
|  | 11 | 0 | 245 | 37.0 |
|  |  | 1 | 417 | 63.0 |
|  | 12 | 0 | 243 | 36.7 |
|  |  | 1 | 419 | 63.3 |
|  | 13 | 0 | 248 | 37.5 |
|  |  | 1 | 414 | 62.5 |
|  | 14 | 0 | 243 | 36.7 |
|  |  | 1 | 419 | 63.3 |
|  | 15 | 0 | 242 | 36.6 |
|  |  | 1 | 420 | 63.4 |
|  | 16 | 0 | 240 | 36.3 |
|  |  | 1 | 422 | 63.7 |
|  | 17 | 0 | 244 | 36.9 |
|  |  | 1 | 418 | 63.1 |
|  | 18 | 0 | 241 | 36.4 |
|  |  | 1 | 421 | 63.6 |
|  | 19 | 0 | 244 | 36.9 |
|  |  | 1 | 418 | 63.1 |
|  | 20 | 0 | 250 | 37.8 |
|  |  | 1 | 412 | 62.2 |
|  | 21 | 0 | 244 | 36.9 |
|  |  | 1 | 418 | 63.1 |
|  | 22 | 0 | 245 | 37.0 |
|  |  | 1 | 417 | 63.0 |
|  | 23 | 0 | 247 | 37.3 |
|  |  | 1 | 415 | 62.7 |
|  | 24 | 0 | 241 | 36.4 |
|  |  | 1 | 421 | 63.6 |
|  | 25 | 0 | 244 | 36.9 |
|  |  | 1 | 418 | 63.1 |
|  | 26 | 0 | 243 | 36.7 |
|  |  | 1 | 419 | 63.3 |
|  | 27 | 0 | 244 | 36.9 |
|  |  | 1 | 418 | 63.1 |
|  | 28 | 0 | 245 | 37.0 |
|  |  | 1 | 417 | 63.0 |
|  | 29 | 0 | 244 | 36.9 |
|  |  | 1 | 418 | 63.1 |
|  | 30 | 0 | 245 | 37.0 |
|  |  | 1 | 417 | 63.0 |
|  | 31 | 0 | 241 | 36.4 |
|  |  | 1 | 421 | 63.6 |
|  | 32 | 0 | 247 | 37.3 |
|  |  | 1 | 415 | 62.7 |
|  | 33 | 0 | 245 | 37.0 |
|  |  | 1 | 417 | 63.0 |
|  | 34 | 0 | 244 | 36.9 |
|  |  | 1 | 418 | 63.1 |
|  | 35 | 0 | 247 | 37.3 |
|  |  | 1 | 415 | 62.7 |
|  | 36 | 0 | 242 | 36.6 |
|  |  | 1 | 420 | 63.4 |
|  | 37 | 0 | 244 | 36.9 |
|  |  | 1 | 418 | 63.1 |
|  | 38 | 0 | 247 | 37.3 |
|  |  | 1 | 415 | 62.7 |
|  | 39 | 0 | 245 | 37.0 |
|  |  | 1 | 417 | 63.0 |
|  | 40 | 0 | 242 | 36.6 |
|  |  | 1 | 420 | 63.4 |
|  | 41 | 0 | 241 | 36.4 |
|  |  | 1 | 421 | 63.6 |
|  | 42 | 0 | 241 | 36.4 |
|  |  | 1 | 421 | 63.6 |
|  | 43 | 0 | 245 | 37.0 |
|  |  | 1 | 417 | 63.0 |
|  | 44 | 0 | 247 | 37.3 |
|  |  | 1 | 415 | 62.7 |
|  | 45 | 0 | 243 | 36.7 |
|  |  | 1 | 419 | 63.3 |
|  | 46 | 0 | 245 | 37.0 |
|  |  | 1 | 417 | 63.0 |
|  | 47 | 0 | 239 | 36.1 |
|  |  | 1 | 423 | 63.9 |
|  | 48 | 0 | 236 | 35.6 |
|  |  | 1 | 426 | 64.4 |
|  | 49 | 0 | 244 | 36.9 |
|  |  | 1 | 418 | 63.1 |
|  | 50 | 0 | 244 | 36.9 |
|  |  | 1 | 418 | 63.1 |

| **Smear_SCR_2months** | | | | |
| --- | --- | --- | --- | --- |
| Data | Imputation | Category | N | Percent |
| Original Data |  | 0 | 83 | 14.2 |
|  |  | 1 | 503 | 85.8 |
| Imputed Values | 1 | 0 | 20 | 26.3 |
|  |  | 1 | 56 | 73.7 |
|  | 2 | 0 | 16 | 21.1 |
|  |  | 1 | 60 | 78.9 |
|  | 3 | 0 | 7 | 9.2 |
|  |  | 1 | 69 | 90.8 |
|  | 4 | 0 | 14 | 18.4 |
|  |  | 1 | 62 | 81.6 |
|  | 5 | 0 | 25 | 32.9 |
|  |  | 1 | 51 | 67.1 |
|  | 6 | 0 | 17 | 22.4 |
|  |  | 1 | 59 | 77.6 |
|  | 7 | 0 | 19 | 25.0 |
|  |  | 1 | 57 | 75.0 |
|  | 8 | 0 | 19 | 25.0 |
|  |  | 1 | 57 | 75.0 |
|  | 9 | 0 | 21 | 27.6 |
|  |  | 1 | 55 | 72.4 |
|  | 10 | 0 | 16 | 21.1 |
|  |  | 1 | 60 | 78.9 |
|  | 11 | 0 | 18 | 23.7 |
|  |  | 1 | 58 | 76.3 |
|  | 12 | 0 | 22 | 28.9 |
|  |  | 1 | 54 | 71.1 |
|  | 13 | 0 | 10 | 13.2 |
|  |  | 1 | 66 | 86.8 |
|  | 14 | 0 | 16 | 21.1 |
|  |  | 1 | 60 | 78.9 |
|  | 15 | 0 | 16 | 21.1 |
|  |  | 1 | 60 | 78.9 |
|  | 16 | 0 | 19 | 25.0 |
|  |  | 1 | 57 | 75.0 |
|  | 17 | 0 | 28 | 36.8 |
|  |  | 1 | 48 | 63.2 |
|  | 18 | 0 | 24 | 31.6 |
|  |  | 1 | 52 | 68.4 |
|  | 19 | 0 | 17 | 22.4 |
|  |  | 1 | 59 | 77.6 |
|  | 20 | 0 | 17 | 22.4 |
|  |  | 1 | 59 | 77.6 |
|  | 21 | 0 | 21 | 27.6 |
|  |  | 1 | 55 | 72.4 |
|  | 22 | 0 | 8 | 10.5 |
|  |  | 1 | 68 | 89.5 |
|  | 23 | 0 | 25 | 32.9 |
|  |  | 1 | 51 | 67.1 |
|  | 24 | 0 | 19 | 25.0 |
|  |  | 1 | 57 | 75.0 |
|  | 25 | 0 | 16 | 21.1 |
|  |  | 1 | 60 | 78.9 |
|  | 26 | 0 | 18 | 23.7 |
|  |  | 1 | 58 | 76.3 |
|  | 27 | 0 | 22 | 28.9 |
|  |  | 1 | 54 | 71.1 |
|  | 28 | 0 | 13 | 17.1 |
|  |  | 1 | 63 | 82.9 |
|  | 29 | 0 | 26 | 34.2 |
|  |  | 1 | 50 | 65.8 |
|  | 30 | 0 | 22 | 28.9 |
|  |  | 1 | 54 | 71.1 |
|  | 31 | 0 | 15 | 19.7 |
|  |  | 1 | 61 | 80.3 |
|  | 32 | 0 | 10 | 13.2 |
|  |  | 1 | 66 | 86.8 |
|  | 33 | 0 | 19 | 25.0 |
|  |  | 1 | 57 | 75.0 |
|  | 34 | 0 | 20 | 26.3 |
|  |  | 1 | 56 | 73.7 |
|  | 35 | 0 | 19 | 25.0 |
|  |  | 1 | 57 | 75.0 |
|  | 36 | 0 | 20 | 26.3 |
|  |  | 1 | 56 | 73.7 |
|  | 37 | 0 | 17 | 22.4 |
|  |  | 1 | 59 | 77.6 |
|  | 38 | 0 | 14 | 18.4 |
|  |  | 1 | 62 | 81.6 |
|  | 39 | 0 | 22 | 28.9 |
|  |  | 1 | 54 | 71.1 |
|  | 40 | 0 | 16 | 21.1 |
|  |  | 1 | 60 | 78.9 |
|  | 41 | 0 | 21 | 27.6 |
|  |  | 1 | 55 | 72.4 |
|  | 42 | 0 | 27 | 35.5 |
|  |  | 1 | 49 | 64.5 |
|  | 43 | 0 | 10 | 13.2 |
|  |  | 1 | 66 | 86.8 |
|  | 44 | 0 | 18 | 23.7 |
|  |  | 1 | 58 | 76.3 |
|  | 45 | 0 | 32 | 42.1 |
|  |  | 1 | 44 | 57.9 |
|  | 46 | 0 | 23 | 30.3 |
|  |  | 1 | 53 | 69.7 |
|  | 47 | 0 | 30 | 39.5 |
|  |  | 1 | 46 | 60.5 |
|  | 48 | 0 | 15 | 19.7 |
|  |  | 1 | 61 | 80.3 |
|  | 49 | 0 | 17 | 22.4 |
|  |  | 1 | 59 | 77.6 |
|  | 50 | 0 | 11 | 14.5 |
|  |  | 1 | 65 | 85.5 |
| Complete Data After Imputation | 1 | 0 | 103 | 15.6 |
|  |  | 1 | 559 | 84.4 |
|  | 2 | 0 | 99 | 15.0 |
|  |  | 1 | 563 | 85.0 |
|  | 3 | 0 | 90 | 13.6 |
|  |  | 1 | 572 | 86.4 |
|  | 4 | 0 | 97 | 14.7 |
|  |  | 1 | 565 | 85.3 |
|  | 5 | 0 | 108 | 16.3 |
|  |  | 1 | 554 | 83.7 |
|  | 6 | 0 | 100 | 15.1 |
|  |  | 1 | 562 | 84.9 |
|  | 7 | 0 | 102 | 15.4 |
|  |  | 1 | 560 | 84.6 |
|  | 8 | 0 | 102 | 15.4 |
|  |  | 1 | 560 | 84.6 |
|  | 9 | 0 | 104 | 15.7 |
|  |  | 1 | 558 | 84.3 |
|  | 10 | 0 | 99 | 15.0 |
|  |  | 1 | 563 | 85.0 |
|  | 11 | 0 | 101 | 15.3 |
|  |  | 1 | 561 | 84.7 |
|  | 12 | 0 | 105 | 15.9 |
|  |  | 1 | 557 | 84.1 |
|  | 13 | 0 | 93 | 14.0 |
|  |  | 1 | 569 | 86.0 |
|  | 14 | 0 | 99 | 15.0 |
|  |  | 1 | 563 | 85.0 |
|  | 15 | 0 | 99 | 15.0 |
|  |  | 1 | 563 | 85.0 |
|  | 16 | 0 | 102 | 15.4 |
|  |  | 1 | 560 | 84.6 |
|  | 17 | 0 | 111 | 16.8 |
|  |  | 1 | 551 | 83.2 |
|  | 18 | 0 | 107 | 16.2 |
|  |  | 1 | 555 | 83.8 |
|  | 19 | 0 | 100 | 15.1 |
|  |  | 1 | 562 | 84.9 |
|  | 20 | 0 | 100 | 15.1 |
|  |  | 1 | 562 | 84.9 |
|  | 21 | 0 | 104 | 15.7 |
|  |  | 1 | 558 | 84.3 |
|  | 22 | 0 | 91 | 13.7 |
|  |  | 1 | 571 | 86.3 |
|  | 23 | 0 | 108 | 16.3 |
|  |  | 1 | 554 | 83.7 |
|  | 24 | 0 | 102 | 15.4 |
|  |  | 1 | 560 | 84.6 |
|  | 25 | 0 | 99 | 15.0 |
|  |  | 1 | 563 | 85.0 |
|  | 26 | 0 | 101 | 15.3 |
|  |  | 1 | 561 | 84.7 |
|  | 27 | 0 | 105 | 15.9 |
|  |  | 1 | 557 | 84.1 |
|  | 28 | 0 | 96 | 14.5 |
|  |  | 1 | 566 | 85.5 |
|  | 29 | 0 | 109 | 16.5 |
|  |  | 1 | 553 | 83.5 |
|  | 30 | 0 | 105 | 15.9 |
|  |  | 1 | 557 | 84.1 |
|  | 31 | 0 | 98 | 14.8 |
|  |  | 1 | 564 | 85.2 |
|  | 32 | 0 | 93 | 14.0 |
|  |  | 1 | 569 | 86.0 |
|  | 33 | 0 | 102 | 15.4 |
|  |  | 1 | 560 | 84.6 |
|  | 34 | 0 | 103 | 15.6 |
|  |  | 1 | 559 | 84.4 |
|  | 35 | 0 | 102 | 15.4 |
|  |  | 1 | 560 | 84.6 |
|  | 36 | 0 | 103 | 15.6 |
|  |  | 1 | 559 | 84.4 |
|  | 37 | 0 | 100 | 15.1 |
|  |  | 1 | 562 | 84.9 |
|  | 38 | 0 | 97 | 14.7 |
|  |  | 1 | 565 | 85.3 |
|  | 39 | 0 | 105 | 15.9 |
|  |  | 1 | 557 | 84.1 |
|  | 40 | 0 | 99 | 15.0 |
|  |  | 1 | 563 | 85.0 |
|  | 41 | 0 | 104 | 15.7 |
|  |  | 1 | 558 | 84.3 |
|  | 42 | 0 | 110 | 16.6 |
|  |  | 1 | 552 | 83.4 |
|  | 43 | 0 | 93 | 14.0 |
|  |  | 1 | 569 | 86.0 |
|  | 44 | 0 | 101 | 15.3 |
|  |  | 1 | 561 | 84.7 |
|  | 45 | 0 | 115 | 17.4 |
|  |  | 1 | 547 | 82.6 |
|  | 46 | 0 | 106 | 16.0 |
|  |  | 1 | 556 | 84.0 |
|  | 47 | 0 | 113 | 17.1 |
|  |  | 1 | 549 | 82.9 |
|  | 48 | 0 | 98 | 14.8 |
|  |  | 1 | 564 | 85.2 |
|  | 49 | 0 | 100 | 15.1 |
|  |  | 1 | 562 | 84.9 |
|  | 50 | 0 | 94 | 14.2 |
|  |  | 1 | 568 | 85.8 |

| **Culture_SCR_2months** | | | | |
| --- | --- | --- | --- | --- |
| Data | Imputation | Category | N | Percent |
| Original Data |  | 0 | 138 | 25.0 |
|  |  | 1 | 415 | 75.0 |
| Imputed Values | 1 | 0 | 36 | 33.0 |
|  |  | 1 | 73 | 67.0 |
|  | 2 | 0 | 39 | 35.8 |
|  |  | 1 | 70 | 64.2 |
|  | 3 | 0 | 36 | 33.0 |
|  |  | 1 | 73 | 67.0 |
|  | 4 | 0 | 22 | 20.2 |
|  |  | 1 | 87 | 79.8 |
|  | 5 | 0 | 35 | 32.1 |
|  |  | 1 | 74 | 67.9 |
|  | 6 | 0 | 34 | 31.2 |
|  |  | 1 | 75 | 68.8 |
|  | 7 | 0 | 28 | 25.7 |
|  |  | 1 | 81 | 74.3 |
|  | 8 | 0 | 32 | 29.4 |
|  |  | 1 | 77 | 70.6 |
|  | 9 | 0 | 31 | 28.4 |
|  |  | 1 | 78 | 71.6 |
|  | 10 | 0 | 25 | 22.9 |
|  |  | 1 | 84 | 77.1 |
|  | 11 | 0 | 26 | 23.9 |
|  |  | 1 | 83 | 76.1 |
|  | 12 | 0 | 43 | 39.4 |
|  |  | 1 | 66 | 60.6 |
|  | 13 | 0 | 40 | 36.7 |
|  |  | 1 | 69 | 63.3 |
|  | 14 | 0 | 29 | 26.6 |
|  |  | 1 | 80 | 73.4 |
|  | 15 | 0 | 38 | 34.9 |
|  |  | 1 | 71 | 65.1 |
|  | 16 | 0 | 30 | 27.5 |
|  |  | 1 | 79 | 72.5 |
|  | 17 | 0 | 52 | 47.7 |
|  |  | 1 | 57 | 52.3 |
|  | 18 | 0 | 37 | 33.9 |
|  |  | 1 | 72 | 66.1 |
|  | 19 | 0 | 36 | 33.0 |
|  |  | 1 | 73 | 67.0 |
|  | 20 | 0 | 28 | 25.7 |
|  |  | 1 | 81 | 74.3 |
|  | 21 | 0 | 34 | 31.2 |
|  |  | 1 | 75 | 68.8 |
|  | 22 | 0 | 28 | 25.7 |
|  |  | 1 | 81 | 74.3 |
|  | 23 | 0 | 32 | 29.4 |
|  |  | 1 | 77 | 70.6 |
|  | 24 | 0 | 41 | 37.6 |
|  |  | 1 | 68 | 62.4 |
|  | 25 | 0 | 28 | 25.7 |
|  |  | 1 | 81 | 74.3 |
|  | 26 | 0 | 43 | 39.4 |
|  |  | 1 | 66 | 60.6 |
|  | 27 | 0 | 36 | 33.0 |
|  |  | 1 | 73 | 67.0 |
|  | 28 | 0 | 31 | 28.4 |
|  |  | 1 | 78 | 71.6 |
|  | 29 | 0 | 40 | 36.7 |
|  |  | 1 | 69 | 63.3 |
|  | 30 | 0 | 31 | 28.4 |
|  |  | 1 | 78 | 71.6 |
|  | 31 | 0 | 39 | 35.8 |
|  |  | 1 | 70 | 64.2 |
|  | 32 | 0 | 33 | 30.3 |
|  |  | 1 | 76 | 69.7 |
|  | 33 | 0 | 28 | 25.7 |
|  |  | 1 | 81 | 74.3 |
|  | 34 | 0 | 31 | 28.4 |
|  |  | 1 | 78 | 71.6 |
|  | 35 | 0 | 28 | 25.7 |
|  |  | 1 | 81 | 74.3 |
|  | 36 | 0 | 34 | 31.2 |
|  |  | 1 | 75 | 68.8 |
|  | 37 | 0 | 31 | 28.4 |
|  |  | 1 | 78 | 71.6 |
|  | 38 | 0 | 35 | 32.1 |
|  |  | 1 | 74 | 67.9 |
|  | 39 | 0 | 39 | 35.8 |
|  |  | 1 | 70 | 64.2 |
|  | 40 | 0 | 34 | 31.2 |
|  |  | 1 | 75 | 68.8 |
|  | 41 | 0 | 39 | 35.8 |
|  |  | 1 | 70 | 64.2 |
|  | 42 | 0 | 43 | 39.4 |
|  |  | 1 | 66 | 60.6 |
|  | 43 | 0 | 33 | 30.3 |
|  |  | 1 | 76 | 69.7 |
|  | 44 | 0 | 36 | 33.0 |
|  |  | 1 | 73 | 67.0 |
|  | 45 | 0 | 40 | 36.7 |
|  |  | 1 | 69 | 63.3 |
|  | 46 | 0 | 45 | 41.3 |
|  |  | 1 | 64 | 58.7 |
|  | 47 | 0 | 44 | 40.4 |
|  |  | 1 | 65 | 59.6 |
|  | 48 | 0 | 36 | 33.0 |
|  |  | 1 | 73 | 67.0 |
|  | 49 | 0 | 37 | 33.9 |
|  |  | 1 | 72 | 66.1 |
|  | 50 | 0 | 26 | 23.9 |
|  |  | 1 | 83 | 76.1 |
| Complete Data After Imputation | 1 | 0 | 174 | 26.3 |
|  |  | 1 | 488 | 73.7 |
|  | 2 | 0 | 177 | 26.7 |
|  |  | 1 | 485 | 73.3 |
|  | 3 | 0 | 174 | 26.3 |
|  |  | 1 | 488 | 73.7 |
|  | 4 | 0 | 160 | 24.2 |
|  |  | 1 | 502 | 75.8 |
|  | 5 | 0 | 173 | 26.1 |
|  |  | 1 | 489 | 73.9 |
|  | 6 | 0 | 172 | 26.0 |
|  |  | 1 | 490 | 74.0 |
|  | 7 | 0 | 166 | 25.1 |
|  |  | 1 | 496 | 74.9 |
|  | 8 | 0 | 170 | 25.7 |
|  |  | 1 | 492 | 74.3 |
|  | 9 | 0 | 169 | 25.5 |
|  |  | 1 | 493 | 74.5 |
|  | 10 | 0 | 163 | 24.6 |
|  |  | 1 | 499 | 75.4 |
|  | 11 | 0 | 164 | 24.8 |
|  |  | 1 | 498 | 75.2 |
|  | 12 | 0 | 181 | 27.3 |
|  |  | 1 | 481 | 72.7 |
|  | 13 | 0 | 178 | 26.9 |
|  |  | 1 | 484 | 73.1 |
|  | 14 | 0 | 167 | 25.2 |
|  |  | 1 | 495 | 74.8 |
|  | 15 | 0 | 176 | 26.6 |
|  |  | 1 | 486 | 73.4 |
|  | 16 | 0 | 168 | 25.4 |
|  |  | 1 | 494 | 74.6 |
|  | 17 | 0 | 190 | 28.7 |
|  |  | 1 | 472 | 71.3 |
|  | 18 | 0 | 175 | 26.4 |
|  |  | 1 | 487 | 73.6 |
|  | 19 | 0 | 174 | 26.3 |
|  |  | 1 | 488 | 73.7 |
|  | 20 | 0 | 166 | 25.1 |
|  |  | 1 | 496 | 74.9 |
|  | 21 | 0 | 172 | 26.0 |
|  |  | 1 | 490 | 74.0 |
|  | 22 | 0 | 166 | 25.1 |
|  |  | 1 | 496 | 74.9 |
|  | 23 | 0 | 170 | 25.7 |
|  |  | 1 | 492 | 74.3 |
|  | 24 | 0 | 179 | 27.0 |
|  |  | 1 | 483 | 73.0 |
|  | 25 | 0 | 166 | 25.1 |
|  |  | 1 | 496 | 74.9 |
|  | 26 | 0 | 181 | 27.3 |
|  |  | 1 | 481 | 72.7 |
|  | 27 | 0 | 174 | 26.3 |
|  |  | 1 | 488 | 73.7 |
|  | 28 | 0 | 169 | 25.5 |
|  |  | 1 | 493 | 74.5 |
|  | 29 | 0 | 178 | 26.9 |
|  |  | 1 | 484 | 73.1 |
|  | 30 | 0 | 169 | 25.5 |
|  |  | 1 | 493 | 74.5 |
|  | 31 | 0 | 177 | 26.7 |
|  |  | 1 | 485 | 73.3 |
|  | 32 | 0 | 171 | 25.8 |
|  |  | 1 | 491 | 74.2 |
|  | 33 | 0 | 166 | 25.1 |
|  |  | 1 | 496 | 74.9 |
|  | 34 | 0 | 169 | 25.5 |
|  |  | 1 | 493 | 74.5 |
|  | 35 | 0 | 166 | 25.1 |
|  |  | 1 | 496 | 74.9 |
|  | 36 | 0 | 172 | 26.0 |
|  |  | 1 | 490 | 74.0 |
|  | 37 | 0 | 169 | 25.5 |
|  |  | 1 | 493 | 74.5 |
|  | 38 | 0 | 173 | 26.1 |
|  |  | 1 | 489 | 73.9 |
|  | 39 | 0 | 177 | 26.7 |
|  |  | 1 | 485 | 73.3 |
|  | 40 | 0 | 172 | 26.0 |
|  |  | 1 | 490 | 74.0 |
|  | 41 | 0 | 177 | 26.7 |
|  |  | 1 | 485 | 73.3 |
|  | 42 | 0 | 181 | 27.3 |
|  |  | 1 | 481 | 72.7 |
|  | 43 | 0 | 171 | 25.8 |
|  |  | 1 | 491 | 74.2 |
|  | 44 | 0 | 174 | 26.3 |
|  |  | 1 | 488 | 73.7 |
|  | 45 | 0 | 178 | 26.9 |
|  |  | 1 | 484 | 73.1 |
|  | 46 | 0 | 183 | 27.6 |
|  |  | 1 | 479 | 72.4 |
|  | 47 | 0 | 182 | 27.5 |
|  |  | 1 | 480 | 72.5 |
|  | 48 | 0 | 174 | 26.3 |
|  |  | 1 | 488 | 73.7 |
|  | 49 | 0 | 175 | 26.4 |
|  |  | 1 | 487 | 73.6 |
|  | 50 | 0 | 164 | 24.8 |
|  |  | 1 | 498 | 75.2 |

| **Smear_conv_4months** | | | | |
| --- | --- | --- | --- | --- |
| Data | Imputation | Category | N | Percent |
| Original Data |  | 0 | 18 | 3.4 |
|  |  | 1 | 519 | 96.6 |
| Imputed Values | 1 | 0 | 24 | 19.2 |
|  |  | 1 | 101 | 80.8 |
|  | 2 | 0 | 9 | 7.2 |
|  |  | 1 | 116 | 92.8 |
|  | 3 | 0 | 25 | 20.0 |
|  |  | 1 | 100 | 80.0 |
|  | 4 | 0 | 40 | 32.0 |
|  |  | 1 | 85 | 68.0 |
|  | 5 | 0 | 29 | 23.2 |
|  |  | 1 | 96 | 76.8 |
|  | 6 | 0 | 6 | 4.8 |
|  |  | 1 | 119 | 95.2 |
|  | 7 | 0 | 37 | 29.6 |
|  |  | 1 | 88 | 70.4 |
|  | 8 | 0 | 35 | 28.0 |
|  |  | 1 | 90 | 72.0 |
|  | 9 | 0 | 30 | 24.0 |
|  |  | 1 | 95 | 76.0 |
|  | 10 | 0 | 44 | 35.2 |
|  |  | 1 | 81 | 64.8 |
|  | 11 | 0 | 29 | 23.2 |
|  |  | 1 | 96 | 76.8 |
|  | 12 | 0 | 38 | 30.4 |
|  |  | 1 | 87 | 69.6 |
|  | 13 | 0 | 37 | 29.6 |
|  |  | 1 | 88 | 70.4 |
|  | 14 | 0 | 40 | 32.0 |
|  |  | 1 | 85 | 68.0 |
|  | 15 | 0 | 29 | 23.2 |
|  |  | 1 | 96 | 76.8 |
|  | 16 | 0 | 9 | 7.2 |
|  |  | 1 | 116 | 92.8 |
|  | 17 | 0 | 33 | 26.4 |
|  |  | 1 | 92 | 73.6 |
|  | 18 | 0 | 27 | 21.6 |
|  |  | 1 | 98 | 78.4 |
|  | 19 | 0 | 3 | 2.4 |
|  |  | 1 | 122 | 97.6 |
|  | 20 | 0 | 19 | 15.2 |
|  |  | 1 | 106 | 84.8 |
|  | 21 | 0 | 34 | 27.2 |
|  |  | 1 | 91 | 72.8 |
|  | 22 | 0 | 11 | 8.8 |
|  |  | 1 | 114 | 91.2 |
|  | 23 | 0 | 24 | 19.2 |
|  |  | 1 | 101 | 80.8 |
|  | 24 | 0 | 6 | 4.8 |
|  |  | 1 | 119 | 95.2 |
|  | 25 | 0 | 19 | 15.2 |
|  |  | 1 | 106 | 84.8 |
|  | 26 | 0 | 47 | 37.6 |
|  |  | 1 | 78 | 62.4 |
|  | 27 | 0 | 37 | 29.6 |
|  |  | 1 | 88 | 70.4 |
|  | 28 | 0 | 29 | 23.2 |
|  |  | 1 | 96 | 76.8 |
|  | 29 | 0 | 12 | 9.6 |
|  |  | 1 | 113 | 90.4 |
|  | 30 | 0 | 26 | 20.8 |
|  |  | 1 | 99 | 79.2 |
|  | 31 | 0 | 11 | 8.8 |
|  |  | 1 | 114 | 91.2 |
|  | 32 | 0 | 45 | 36.0 |
|  |  | 1 | 80 | 64.0 |
|  | 33 | 0 | 13 | 10.4 |
|  |  | 1 | 112 | 89.6 |
|  | 34 | 0 | 27 | 21.6 |
|  |  | 1 | 98 | 78.4 |
|  | 35 | 0 | 8 | 6.4 |
|  |  | 1 | 117 | 93.6 |
|  | 36 | 0 | 34 | 27.2 |
|  |  | 1 | 91 | 72.8 |
|  | 37 | 0 | 35 | 28.0 |
|  |  | 1 | 90 | 72.0 |
|  | 38 | 0 | 19 | 15.2 |
|  |  | 1 | 106 | 84.8 |
|  | 39 | 0 | 23 | 18.4 |
|  |  | 1 | 102 | 81.6 |
|  | 40 | 0 | 28 | 22.4 |
|  |  | 1 | 97 | 77.6 |
|  | 41 | 0 | 32 | 25.6 |
|  |  | 1 | 93 | 74.4 |
|  | 42 | 0 | 11 | 8.8 |
|  |  | 1 | 114 | 91.2 |
|  | 43 | 0 | 47 | 37.6 |
|  |  | 1 | 78 | 62.4 |
|  | 44 | 0 | 35 | 28.0 |
|  |  | 1 | 90 | 72.0 |
|  | 45 | 0 | 18 | 14.4 |
|  |  | 1 | 107 | 85.6 |
|  | 46 | 0 | 16 | 12.8 |
|  |  | 1 | 109 | 87.2 |
|  | 47 | 0 | 6 | 4.8 |
|  |  | 1 | 119 | 95.2 |
|  | 48 | 0 | 30 | 24.0 |
|  |  | 1 | 95 | 76.0 |
|  | 49 | 0 | 20 | 16.0 |
|  |  | 1 | 105 | 84.0 |
|  | 50 | 0 | 18 | 14.4 |
|  |  | 1 | 107 | 85.6 |
| Complete Data After Imputation | 1 | 0 | 42 | 6.3 |
|  |  | 1 | 620 | 93.7 |
|  | 2 | 0 | 27 | 4.1 |
|  |  | 1 | 635 | 95.9 |
|  | 3 | 0 | 43 | 6.5 |
|  |  | 1 | 619 | 93.5 |
|  | 4 | 0 | 58 | 8.8 |
|  |  | 1 | 604 | 91.2 |
|  | 5 | 0 | 47 | 7.1 |
|  |  | 1 | 615 | 92.9 |
|  | 6 | 0 | 24 | 3.6 |
|  |  | 1 | 638 | 96.4 |
|  | 7 | 0 | 55 | 8.3 |
|  |  | 1 | 607 | 91.7 |
|  | 8 | 0 | 53 | 8.0 |
|  |  | 1 | 609 | 92.0 |
|  | 9 | 0 | 48 | 7.3 |
|  |  | 1 | 614 | 92.7 |
|  | 10 | 0 | 62 | 9.4 |
|  |  | 1 | 600 | 90.6 |
|  | 11 | 0 | 47 | 7.1 |
|  |  | 1 | 615 | 92.9 |
|  | 12 | 0 | 56 | 8.5 |
|  |  | 1 | 606 | 91.5 |
|  | 13 | 0 | 55 | 8.3 |
|  |  | 1 | 607 | 91.7 |
|  | 14 | 0 | 58 | 8.8 |
|  |  | 1 | 604 | 91.2 |
|  | 15 | 0 | 47 | 7.1 |
|  |  | 1 | 615 | 92.9 |
|  | 16 | 0 | 27 | 4.1 |
|  |  | 1 | 635 | 95.9 |
|  | 17 | 0 | 51 | 7.7 |
|  |  | 1 | 611 | 92.3 |
|  | 18 | 0 | 45 | 6.8 |
|  |  | 1 | 617 | 93.2 |
|  | 19 | 0 | 21 | 3.2 |
|  |  | 1 | 641 | 96.8 |
|  | 20 | 0 | 37 | 5.6 |
|  |  | 1 | 625 | 94.4 |
|  | 21 | 0 | 52 | 7.9 |
|  |  | 1 | 610 | 92.1 |
|  | 22 | 0 | 29 | 4.4 |
|  |  | 1 | 633 | 95.6 |
|  | 23 | 0 | 42 | 6.3 |
|  |  | 1 | 620 | 93.7 |
|  | 24 | 0 | 24 | 3.6 |
|  |  | 1 | 638 | 96.4 |
|  | 25 | 0 | 37 | 5.6 |
|  |  | 1 | 625 | 94.4 |
|  | 26 | 0 | 65 | 9.8 |
|  |  | 1 | 597 | 90.2 |
|  | 27 | 0 | 55 | 8.3 |
|  |  | 1 | 607 | 91.7 |
|  | 28 | 0 | 47 | 7.1 |
|  |  | 1 | 615 | 92.9 |
|  | 29 | 0 | 30 | 4.5 |
|  |  | 1 | 632 | 95.5 |
|  | 30 | 0 | 44 | 6.6 |
|  |  | 1 | 618 | 93.4 |
|  | 31 | 0 | 29 | 4.4 |
|  |  | 1 | 633 | 95.6 |
|  | 32 | 0 | 63 | 9.5 |
|  |  | 1 | 599 | 90.5 |
|  | 33 | 0 | 31 | 4.7 |
|  |  | 1 | 631 | 95.3 |
|  | 34 | 0 | 45 | 6.8 |
|  |  | 1 | 617 | 93.2 |
|  | 35 | 0 | 26 | 3.9 |
|  |  | 1 | 636 | 96.1 |
|  | 36 | 0 | 52 | 7.9 |
|  |  | 1 | 610 | 92.1 |
|  | 37 | 0 | 53 | 8.0 |
|  |  | 1 | 609 | 92.0 |
|  | 38 | 0 | 37 | 5.6 |
|  |  | 1 | 625 | 94.4 |
|  | 39 | 0 | 41 | 6.2 |
|  |  | 1 | 621 | 93.8 |
|  | 40 | 0 | 46 | 6.9 |
|  |  | 1 | 616 | 93.1 |
|  | 41 | 0 | 50 | 7.6 |
|  |  | 1 | 612 | 92.4 |
|  | 42 | 0 | 29 | 4.4 |
|  |  | 1 | 633 | 95.6 |
|  | 43 | 0 | 65 | 9.8 |
|  |  | 1 | 597 | 90.2 |
|  | 44 | 0 | 53 | 8.0 |
|  |  | 1 | 609 | 92.0 |
|  | 45 | 0 | 36 | 5.4 |
|  |  | 1 | 626 | 94.6 |
|  | 46 | 0 | 34 | 5.1 |
|  |  | 1 | 628 | 94.9 |
|  | 47 | 0 | 24 | 3.6 |
|  |  | 1 | 638 | 96.4 |
|  | 48 | 0 | 48 | 7.3 |
|  |  | 1 | 614 | 92.7 |
|  | 49 | 0 | 38 | 5.7 |
|  |  | 1 | 624 | 94.3 |
|  | 50 | 0 | 36 | 5.4 |
|  |  | 1 | 626 | 94.6 |

| **Culture_conv_4months** | | | | |
| --- | --- | --- | --- | --- |
| Data | Imputation | Category | N | Percent |
| Original Data |  | 0 | 40 | 7.6 |
|  |  | 1 | 488 | 92.4 |
| Imputed Values | 1 | 0 | 20 | 14.9 |
|  |  | 1 | 114 | 85.1 |
|  | 2 | 0 | 23 | 17.2 |
|  |  | 1 | 111 | 82.8 |
|  | 3 | 0 | 28 | 20.9 |
|  |  | 1 | 106 | 79.1 |
|  | 4 | 0 | 27 | 20.1 |
|  |  | 1 | 107 | 79.9 |
|  | 5 | 0 | 22 | 16.4 |
|  |  | 1 | 112 | 83.6 |
|  | 6 | 0 | 17 | 12.7 |
|  |  | 1 | 117 | 87.3 |
|  | 7 | 0 | 45 | 33.6 |
|  |  | 1 | 89 | 66.4 |
|  | 8 | 0 | 21 | 15.7 |
|  |  | 1 | 113 | 84.3 |
|  | 9 | 0 | 14 | 10.4 |
|  |  | 1 | 120 | 89.6 |
|  | 10 | 0 | 17 | 12.7 |
|  |  | 1 | 117 | 87.3 |
|  | 11 | 0 | 26 | 19.4 |
|  |  | 1 | 108 | 80.6 |
|  | 12 | 0 | 22 | 16.4 |
|  |  | 1 | 112 | 83.6 |
|  | 13 | 0 | 21 | 15.7 |
|  |  | 1 | 113 | 84.3 |
|  | 14 | 0 | 37 | 27.6 |
|  |  | 1 | 97 | 72.4 |
|  | 15 | 0 | 23 | 17.2 |
|  |  | 1 | 111 | 82.8 |
|  | 16 | 0 | 24 | 17.9 |
|  |  | 1 | 110 | 82.1 |
|  | 17 | 0 | 17 | 12.7 |
|  |  | 1 | 117 | 87.3 |
|  | 18 | 0 | 26 | 19.4 |
|  |  | 1 | 108 | 80.6 |
|  | 19 | 0 | 19 | 14.2 |
|  |  | 1 | 115 | 85.8 |
|  | 20 | 0 | 16 | 11.9 |
|  |  | 1 | 118 | 88.1 |
|  | 21 | 0 | 29 | 21.6 |
|  |  | 1 | 105 | 78.4 |
|  | 22 | 0 | 13 | 9.7 |
|  |  | 1 | 121 | 90.3 |
|  | 23 | 0 | 21 | 15.7 |
|  |  | 1 | 113 | 84.3 |
|  | 24 | 0 | 30 | 22.4 |
|  |  | 1 | 104 | 77.6 |
|  | 25 | 0 | 34 | 25.4 |
|  |  | 1 | 100 | 74.6 |
|  | 26 | 0 | 34 | 25.4 |
|  |  | 1 | 100 | 74.6 |
|  | 27 | 0 | 18 | 13.4 |
|  |  | 1 | 116 | 86.6 |
|  | 28 | 0 | 15 | 11.2 |
|  |  | 1 | 119 | 88.8 |
|  | 29 | 0 | 18 | 13.4 |
|  |  | 1 | 116 | 86.6 |
|  | 30 | 0 | 22 | 16.4 |
|  |  | 1 | 112 | 83.6 |
|  | 31 | 0 | 48 | 35.8 |
|  |  | 1 | 86 | 64.2 |
|  | 32 | 0 | 12 | 9.0 |
|  |  | 1 | 122 | 91.0 |
|  | 33 | 0 | 24 | 17.9 |
|  |  | 1 | 110 | 82.1 |
|  | 34 | 0 | 25 | 18.7 |
|  |  | 1 | 109 | 81.3 |
|  | 35 | 0 | 15 | 11.2 |
|  |  | 1 | 119 | 88.8 |
|  | 36 | 0 | 30 | 22.4 |
|  |  | 1 | 104 | 77.6 |
|  | 37 | 0 | 26 | 19.4 |
|  |  | 1 | 108 | 80.6 |
|  | 38 | 0 | 29 | 21.6 |
|  |  | 1 | 105 | 78.4 |
|  | 39 | 0 | 25 | 18.7 |
|  |  | 1 | 109 | 81.3 |
|  | 40 | 0 | 16 | 11.9 |
|  |  | 1 | 118 | 88.1 |
|  | 41 | 0 | 28 | 20.9 |
|  |  | 1 | 106 | 79.1 |
|  | 42 | 0 | 23 | 17.2 |
|  |  | 1 | 111 | 82.8 |
|  | 43 | 0 | 18 | 13.4 |
|  |  | 1 | 116 | 86.6 |
|  | 44 | 0 | 39 | 29.1 |
|  |  | 1 | 95 | 70.9 |
|  | 45 | 0 | 40 | 29.9 |
|  |  | 1 | 94 | 70.1 |
|  | 46 | 0 | 15 | 11.2 |
|  |  | 1 | 119 | 88.8 |
|  | 47 | 0 | 15 | 11.2 |
|  |  | 1 | 119 | 88.8 |
|  | 48 | 0 | 32 | 23.9 |
|  |  | 1 | 102 | 76.1 |
|  | 49 | 0 | 35 | 26.1 |
|  |  | 1 | 99 | 73.9 |
|  | 50 | 0 | 12 | 9.0 |
|  |  | 1 | 122 | 91.0 |
| Complete Data After Imputation | 1 | 0 | 60 | 9.1 |
|  |  | 1 | 602 | 90.9 |
|  | 2 | 0 | 63 | 9.5 |
|  |  | 1 | 599 | 90.5 |
|  | 3 | 0 | 68 | 10.3 |
|  |  | 1 | 594 | 89.7 |
|  | 4 | 0 | 67 | 10.1 |
|  |  | 1 | 595 | 89.9 |
|  | 5 | 0 | 62 | 9.4 |
|  |  | 1 | 600 | 90.6 |
|  | 6 | 0 | 57 | 8.6 |
|  |  | 1 | 605 | 91.4 |
|  | 7 | 0 | 85 | 12.8 |
|  |  | 1 | 577 | 87.2 |
|  | 8 | 0 | 61 | 9.2 |
|  |  | 1 | 601 | 90.8 |
|  | 9 | 0 | 54 | 8.2 |
|  |  | 1 | 608 | 91.8 |
|  | 10 | 0 | 57 | 8.6 |
|  |  | 1 | 605 | 91.4 |
|  | 11 | 0 | 66 | 10.0 |
|  |  | 1 | 596 | 90.0 |
|  | 12 | 0 | 62 | 9.4 |
|  |  | 1 | 600 | 90.6 |
|  | 13 | 0 | 61 | 9.2 |
|  |  | 1 | 601 | 90.8 |
|  | 14 | 0 | 77 | 11.6 |
|  |  | 1 | 585 | 88.4 |
|  | 15 | 0 | 63 | 9.5 |
|  |  | 1 | 599 | 90.5 |
|  | 16 | 0 | 64 | 9.7 |
|  |  | 1 | 598 | 90.3 |
|  | 17 | 0 | 57 | 8.6 |
|  |  | 1 | 605 | 91.4 |
|  | 18 | 0 | 66 | 10.0 |
|  |  | 1 | 596 | 90.0 |
|  | 19 | 0 | 59 | 8.9 |
|  |  | 1 | 603 | 91.1 |
|  | 20 | 0 | 56 | 8.5 |
|  |  | 1 | 606 | 91.5 |
|  | 21 | 0 | 69 | 10.4 |
|  |  | 1 | 593 | 89.6 |
|  | 22 | 0 | 53 | 8.0 |
|  |  | 1 | 609 | 92.0 |
|  | 23 | 0 | 61 | 9.2 |
|  |  | 1 | 601 | 90.8 |
|  | 24 | 0 | 70 | 10.6 |
|  |  | 1 | 592 | 89.4 |
|  | 25 | 0 | 74 | 11.2 |
|  |  | 1 | 588 | 88.8 |
|  | 26 | 0 | 74 | 11.2 |
|  |  | 1 | 588 | 88.8 |
|  | 27 | 0 | 58 | 8.8 |
|  |  | 1 | 604 | 91.2 |
|  | 28 | 0 | 55 | 8.3 |
|  |  | 1 | 607 | 91.7 |
|  | 29 | 0 | 58 | 8.8 |
|  |  | 1 | 604 | 91.2 |
|  | 30 | 0 | 62 | 9.4 |
|  |  | 1 | 600 | 90.6 |
|  | 31 | 0 | 88 | 13.3 |
|  |  | 1 | 574 | 86.7 |
|  | 32 | 0 | 52 | 7.9 |
|  |  | 1 | 610 | 92.1 |
|  | 33 | 0 | 64 | 9.7 |
|  |  | 1 | 598 | 90.3 |
|  | 34 | 0 | 65 | 9.8 |
|  |  | 1 | 597 | 90.2 |
|  | 35 | 0 | 55 | 8.3 |
|  |  | 1 | 607 | 91.7 |
|  | 36 | 0 | 70 | 10.6 |
|  |  | 1 | 592 | 89.4 |
|  | 37 | 0 | 66 | 10.0 |
|  |  | 1 | 596 | 90.0 |
|  | 38 | 0 | 69 | 10.4 |
|  |  | 1 | 593 | 89.6 |
|  | 39 | 0 | 65 | 9.8 |
|  |  | 1 | 597 | 90.2 |
|  | 40 | 0 | 56 | 8.5 |
|  |  | 1 | 606 | 91.5 |
|  | 41 | 0 | 68 | 10.3 |
|  |  | 1 | 594 | 89.7 |
|  | 42 | 0 | 63 | 9.5 |
|  |  | 1 | 599 | 90.5 |
|  | 43 | 0 | 58 | 8.8 |
|  |  | 1 | 604 | 91.2 |
|  | 44 | 0 | 79 | 11.9 |
|  |  | 1 | 583 | 88.1 |
|  | 45 | 0 | 80 | 12.1 |
|  |  | 1 | 582 | 87.9 |
|  | 46 | 0 | 55 | 8.3 |
|  |  | 1 | 607 | 91.7 |
|  | 47 | 0 | 55 | 8.3 |
|  |  | 1 | 607 | 91.7 |
|  | 48 | 0 | 72 | 10.9 |
|  |  | 1 | 590 | 89.1 |
|  | 49 | 0 | 75 | 11.3 |
|  |  | 1 | 587 | 88.7 |
|  | 50 | 0 | 52 | 7.9 |
|  |  | 1 | 610 | 92.1 |

| **Inpatient_side_effect_experienced** | | | | |
| --- | --- | --- | --- | --- |
| Data | Imputation | Category | N | Percent |
| Original Data |  | 0 | 413 | 91.4 |
|  |  | 1 | 39 | 8.6 |
| Imputed Values | 1 | 0 | 210 | 100.0 |
|  | 2 | 0 | 56 | 26.7 |
|  |  | 1 | 154 | 73.3 |
|  | 3 | 0 | 9 | 4.3 |
|  |  | 1 | 201 | 95.7 |
|  | 4 | 0 | 136 | 64.8 |
|  |  | 1 | 74 | 35.2 |
|  | 5 | 0 | 79 | 37.6 |
|  |  | 1 | 131 | 62.4 |
|  | 6 | 0 | 27 | 12.9 |
|  |  | 1 | 183 | 87.1 |
|  | 7 | 0 | 151 | 71.9 |
|  |  | 1 | 59 | 28.1 |
|  | 8 | 0 | 203 | 96.7 |
|  |  | 1 | 7 | 3.3 |
|  | 9 | 0 | 6 | 2.9 |
|  |  | 1 | 204 | 97.1 |
|  | 10 | 0 | 135 | 64.3 |
|  |  | 1 | 75 | 35.7 |
|  | 11 | 0 | 21 | 10.0 |
|  |  | 1 | 189 | 90.0 |
|  | 12 | 0 | 209 | 99.5 |
|  |  | 1 | 1 | .5 |
|  | 13 | 0 | 182 | 86.7 |
|  |  | 1 | 28 | 13.3 |
|  | 14 | 0 | 40 | 19.0 |
|  |  | 1 | 170 | 81.0 |
|  | 15 | 0 | 136 | 64.8 |
|  |  | 1 | 74 | 35.2 |
|  | 16 | 0 | 46 | 21.9 |
|  |  | 1 | 164 | 78.1 |
|  | 17 | 0 | 149 | 71.0 |
|  |  | 1 | 61 | 29.0 |
|  | 18 | 0 | 207 | 98.6 |
|  |  | 1 | 3 | 1.4 |
|  | 19 | 0 | 190 | 90.5 |
|  |  | 1 | 20 | 9.5 |
|  | 20 | 0 | 10 | 4.8 |
|  |  | 1 | 200 | 95.2 |
|  | 21 | 0 | 191 | 91.0 |
|  |  | 1 | 19 | 9.0 |
|  | 22 | 0 | 208 | 99.0 |
|  |  | 1 | 2 | 1.0 |
|  | 23 | 0 | 206 | 98.1 |
|  |  | 1 | 4 | 1.9 |
|  | 24 | 0 | 204 | 97.1 |
|  |  | 1 | 6 | 2.9 |
|  | 25 | 0 | 11 | 5.2 |
|  |  | 1 | 199 | 94.8 |
|  | 26 | 0 | 183 | 87.1 |
|  |  | 1 | 27 | 12.9 |
|  | 27 | 0 | 203 | 96.7 |
|  |  | 1 | 7 | 3.3 |
|  | 28 | 0 | 209 | 99.5 |
|  |  | 1 | 1 | .5 |
|  | 29 | 0 | 166 | 79.0 |
|  |  | 1 | 44 | 21.0 |
|  | 30 | 0 | 204 | 97.1 |
|  |  | 1 | 6 | 2.9 |
|  | 31 | 0 | 209 | 99.5 |
|  |  | 1 | 1 | .5 |
|  | 32 | 0 | 187 | 89.0 |
|  |  | 1 | 23 | 11.0 |
|  | 33 | 0 | 90 | 42.9 |
|  |  | 1 | 120 | 57.1 |
|  | 34 | 0 | 8 | 3.8 |
|  |  | 1 | 202 | 96.2 |
|  | 35 | 0 | 164 | 78.1 |
|  |  | 1 | 46 | 21.9 |
|  | 36 | 0 | 10 | 4.8 |
|  |  | 1 | 200 | 95.2 |
|  | 37 | 0 | 18 | 8.6 |
|  |  | 1 | 192 | 91.4 |
|  | 38 | 0 | 194 | 92.4 |
|  |  | 1 | 16 | 7.6 |
|  | 39 | 0 | 24 | 11.4 |
|  |  | 1 | 186 | 88.6 |
|  | 40 | 0 | 58 | 27.6 |
|  |  | 1 | 152 | 72.4 |
|  | 41 | 0 | 206 | 98.1 |
|  |  | 1 | 4 | 1.9 |
|  | 42 | 0 | 30 | 14.3 |
|  |  | 1 | 180 | 85.7 |
|  | 43 | 0 | 153 | 72.9 |
|  |  | 1 | 57 | 27.1 |
|  | 44 | 0 | 35 | 16.7 |
|  |  | 1 | 175 | 83.3 |
|  | 45 | 0 | 190 | 90.5 |
|  |  | 1 | 20 | 9.5 |
|  | 46 | 0 | 56 | 26.7 |
|  |  | 1 | 154 | 73.3 |
|  | 47 | 0 | 195 | 92.9 |
|  |  | 1 | 15 | 7.1 |
|  | 48 | 0 | 180 | 85.7 |
|  |  | 1 | 30 | 14.3 |
|  | 49 | 0 | 75 | 35.7 |
|  |  | 1 | 135 | 64.3 |
|  | 50 | 0 | 165 | 78.6 |
|  |  | 1 | 45 | 21.4 |
| Complete Data After Imputation | 1 | 0 | 623 | 94.1 |
|  |  | 1 | 39 | 5.9 |
|  | 2 | 0 | 469 | 70.8 |
|  |  | 1 | 193 | 29.2 |
|  | 3 | 0 | 422 | 63.7 |
|  |  | 1 | 240 | 36.3 |
|  | 4 | 0 | 549 | 82.9 |
|  |  | 1 | 113 | 17.1 |
|  | 5 | 0 | 492 | 74.3 |
|  |  | 1 | 170 | 25.7 |
|  | 6 | 0 | 440 | 66.5 |
|  |  | 1 | 222 | 33.5 |
|  | 7 | 0 | 564 | 85.2 |
|  |  | 1 | 98 | 14.8 |
|  | 8 | 0 | 616 | 93.1 |
|  |  | 1 | 46 | 6.9 |
|  | 9 | 0 | 419 | 63.3 |
|  |  | 1 | 243 | 36.7 |
|  | 10 | 0 | 548 | 82.8 |
|  |  | 1 | 114 | 17.2 |
|  | 11 | 0 | 434 | 65.6 |
|  |  | 1 | 228 | 34.4 |
|  | 12 | 0 | 622 | 94.0 |
|  |  | 1 | 40 | 6.0 |
|  | 13 | 0 | 595 | 89.9 |
|  |  | 1 | 67 | 10.1 |
|  | 14 | 0 | 453 | 68.4 |
|  |  | 1 | 209 | 31.6 |
|  | 15 | 0 | 549 | 82.9 |
|  |  | 1 | 113 | 17.1 |
|  | 16 | 0 | 459 | 69.3 |
|  |  | 1 | 203 | 30.7 |
|  | 17 | 0 | 562 | 84.9 |
|  |  | 1 | 100 | 15.1 |
|  | 18 | 0 | 620 | 93.7 |
|  |  | 1 | 42 | 6.3 |
|  | 19 | 0 | 603 | 91.1 |
|  |  | 1 | 59 | 8.9 |
|  | 20 | 0 | 423 | 63.9 |
|  |  | 1 | 239 | 36.1 |
|  | 21 | 0 | 604 | 91.2 |
|  |  | 1 | 58 | 8.8 |
|  | 22 | 0 | 621 | 93.8 |
|  |  | 1 | 41 | 6.2 |
|  | 23 | 0 | 619 | 93.5 |
|  |  | 1 | 43 | 6.5 |
|  | 24 | 0 | 617 | 93.2 |
|  |  | 1 | 45 | 6.8 |
|  | 25 | 0 | 424 | 64.0 |
|  |  | 1 | 238 | 36.0 |
|  | 26 | 0 | 596 | 90.0 |
|  |  | 1 | 66 | 10.0 |
|  | 27 | 0 | 616 | 93.1 |
|  |  | 1 | 46 | 6.9 |
|  | 28 | 0 | 622 | 94.0 |
|  |  | 1 | 40 | 6.0 |
|  | 29 | 0 | 579 | 87.5 |
|  |  | 1 | 83 | 12.5 |
|  | 30 | 0 | 617 | 93.2 |
|  |  | 1 | 45 | 6.8 |
|  | 31 | 0 | 622 | 94.0 |
|  |  | 1 | 40 | 6.0 |
|  | 32 | 0 | 600 | 90.6 |
|  |  | 1 | 62 | 9.4 |
|  | 33 | 0 | 503 | 76.0 |
|  |  | 1 | 159 | 24.0 |
|  | 34 | 0 | 421 | 63.6 |
|  |  | 1 | 241 | 36.4 |
|  | 35 | 0 | 577 | 87.2 |
|  |  | 1 | 85 | 12.8 |
|  | 36 | 0 | 423 | 63.9 |
|  |  | 1 | 239 | 36.1 |
|  | 37 | 0 | 431 | 65.1 |
|  |  | 1 | 231 | 34.9 |
|  | 38 | 0 | 607 | 91.7 |
|  |  | 1 | 55 | 8.3 |
|  | 39 | 0 | 437 | 66.0 |
|  |  | 1 | 225 | 34.0 |
|  | 40 | 0 | 471 | 71.1 |
|  |  | 1 | 191 | 28.9 |
|  | 41 | 0 | 619 | 93.5 |
|  |  | 1 | 43 | 6.5 |
|  | 42 | 0 | 443 | 66.9 |
|  |  | 1 | 219 | 33.1 |
|  | 43 | 0 | 566 | 85.5 |
|  |  | 1 | 96 | 14.5 |
|  | 44 | 0 | 448 | 67.7 |
|  |  | 1 | 214 | 32.3 |
|  | 45 | 0 | 603 | 91.1 |
|  |  | 1 | 59 | 8.9 |
|  | 46 | 0 | 469 | 70.8 |
|  |  | 1 | 193 | 29.2 |
|  | 47 | 0 | 608 | 91.8 |
|  |  | 1 | 54 | 8.2 |
|  | 48 | 0 | 593 | 89.6 |
|  |  | 1 | 69 | 10.4 |
|  | 49 | 0 | 488 | 73.7 |
|  |  | 1 | 174 | 26.3 |
|  | 50 | 0 | 578 | 87.3 |
|  |  | 1 | 84 | 12.7 |

| **Outpatient_side_effect_experienced** | | | | |
| --- | --- | --- | --- | --- |
| Data | Imputation | Category | N | Percent |
| Original Data |  | 0 | 465 | 81.3 |
|  |  | 1 | 107 | 18.7 |
| Imputed Values | 1 | 0 | 65 | 72.2 |
|  |  | 1 | 25 | 27.8 |
|  | 2 | 0 | 71 | 78.9 |
|  |  | 1 | 19 | 21.1 |
|  | 3 | 0 | 70 | 77.8 |
|  |  | 1 | 20 | 22.2 |
|  | 4 | 0 | 78 | 86.7 |
|  |  | 1 | 12 | 13.3 |
|  | 5 | 0 | 68 | 75.6 |
|  |  | 1 | 22 | 24.4 |
|  | 6 | 0 | 74 | 82.2 |
|  |  | 1 | 16 | 17.8 |
|  | 7 | 0 | 69 | 76.7 |
|  |  | 1 | 21 | 23.3 |
|  | 8 | 0 | 70 | 77.8 |
|  |  | 1 | 20 | 22.2 |
|  | 9 | 0 | 71 | 78.9 |
|  |  | 1 | 19 | 21.1 |
|  | 10 | 0 | 67 | 74.4 |
|  |  | 1 | 23 | 25.6 |
|  | 11 | 0 | 69 | 76.7 |
|  |  | 1 | 21 | 23.3 |
|  | 12 | 0 | 73 | 81.1 |
|  |  | 1 | 17 | 18.9 |
|  | 13 | 0 | 73 | 81.1 |
|  |  | 1 | 17 | 18.9 |
|  | 14 | 0 | 70 | 77.8 |
|  |  | 1 | 20 | 22.2 |
|  | 15 | 0 | 79 | 87.8 |
|  |  | 1 | 11 | 12.2 |
|  | 16 | 0 | 64 | 71.1 |
|  |  | 1 | 26 | 28.9 |
|  | 17 | 0 | 62 | 68.9 |
|  |  | 1 | 28 | 31.1 |
|  | 18 | 0 | 68 | 75.6 |
|  |  | 1 | 22 | 24.4 |
|  | 19 | 0 | 68 | 75.6 |
|  |  | 1 | 22 | 24.4 |
|  | 20 | 0 | 71 | 78.9 |
|  |  | 1 | 19 | 21.1 |
|  | 21 | 0 | 70 | 77.8 |
|  |  | 1 | 20 | 22.2 |
|  | 22 | 0 | 73 | 81.1 |
|  |  | 1 | 17 | 18.9 |
|  | 23 | 0 | 72 | 80.0 |
|  |  | 1 | 18 | 20.0 |
|  | 24 | 0 | 71 | 78.9 |
|  |  | 1 | 19 | 21.1 |
|  | 25 | 0 | 69 | 76.7 |
|  |  | 1 | 21 | 23.3 |
|  | 26 | 0 | 65 | 72.2 |
|  |  | 1 | 25 | 27.8 |
|  | 27 | 0 | 68 | 75.6 |
|  |  | 1 | 22 | 24.4 |
|  | 28 | 0 | 82 | 91.1 |
|  |  | 1 | 8 | 8.9 |
|  | 29 | 0 | 61 | 67.8 |
|  |  | 1 | 29 | 32.2 |
|  | 30 | 0 | 76 | 84.4 |
|  |  | 1 | 14 | 15.6 |
|  | 31 | 0 | 64 | 71.1 |
|  |  | 1 | 26 | 28.9 |
|  | 32 | 0 | 62 | 68.9 |
|  |  | 1 | 28 | 31.1 |
|  | 33 | 0 | 73 | 81.1 |
|  |  | 1 | 17 | 18.9 |
|  | 34 | 0 | 68 | 75.6 |
|  |  | 1 | 22 | 24.4 |
|  | 35 | 0 | 68 | 75.6 |
|  |  | 1 | 22 | 24.4 |
|  | 36 | 0 | 72 | 80.0 |
|  |  | 1 | 18 | 20.0 |
|  | 37 | 0 | 65 | 72.2 |
|  |  | 1 | 25 | 27.8 |
|  | 38 | 0 | 77 | 85.6 |
|  |  | 1 | 13 | 14.4 |
|  | 39 | 0 | 69 | 76.7 |
|  |  | 1 | 21 | 23.3 |
|  | 40 | 0 | 69 | 76.7 |
|  |  | 1 | 21 | 23.3 |
|  | 41 | 0 | 75 | 83.3 |
|  |  | 1 | 15 | 16.7 |
|  | 42 | 0 | 74 | 82.2 |
|  |  | 1 | 16 | 17.8 |
|  | 43 | 0 | 72 | 80.0 |
|  |  | 1 | 18 | 20.0 |
|  | 44 | 0 | 72 | 80.0 |
|  |  | 1 | 18 | 20.0 |
|  | 45 | 0 | 71 | 78.9 |
|  |  | 1 | 19 | 21.1 |
|  | 46 | 0 | 72 | 80.0 |
|  |  | 1 | 18 | 20.0 |
|  | 47 | 0 | 76 | 84.4 |
|  |  | 1 | 14 | 15.6 |
|  | 48 | 0 | 61 | 67.8 |
|  |  | 1 | 29 | 32.2 |
|  | 49 | 0 | 62 | 68.9 |
|  |  | 1 | 28 | 31.1 |
|  | 50 | 0 | 61 | 67.8 |
|  |  | 1 | 29 | 32.2 |
| Complete Data After Imputation | 1 | 0 | 530 | 80.1 |
|  |  | 1 | 132 | 19.9 |
|  | 2 | 0 | 536 | 81.0 |
|  |  | 1 | 126 | 19.0 |
|  | 3 | 0 | 535 | 80.8 |
|  |  | 1 | 127 | 19.2 |
|  | 4 | 0 | 543 | 82.0 |
|  |  | 1 | 119 | 18.0 |
|  | 5 | 0 | 533 | 80.5 |
|  |  | 1 | 129 | 19.5 |
|  | 6 | 0 | 539 | 81.4 |
|  |  | 1 | 123 | 18.6 |
|  | 7 | 0 | 534 | 80.7 |
|  |  | 1 | 128 | 19.3 |
|  | 8 | 0 | 535 | 80.8 |
|  |  | 1 | 127 | 19.2 |
|  | 9 | 0 | 536 | 81.0 |
|  |  | 1 | 126 | 19.0 |
|  | 10 | 0 | 532 | 80.4 |
|  |  | 1 | 130 | 19.6 |
|  | 11 | 0 | 534 | 80.7 |
|  |  | 1 | 128 | 19.3 |
|  | 12 | 0 | 538 | 81.3 |
|  |  | 1 | 124 | 18.7 |
|  | 13 | 0 | 538 | 81.3 |
|  |  | 1 | 124 | 18.7 |
|  | 14 | 0 | 535 | 80.8 |
|  |  | 1 | 127 | 19.2 |
|  | 15 | 0 | 544 | 82.2 |
|  |  | 1 | 118 | 17.8 |
|  | 16 | 0 | 529 | 79.9 |
|  |  | 1 | 133 | 20.1 |
|  | 17 | 0 | 527 | 79.6 |
|  |  | 1 | 135 | 20.4 |
|  | 18 | 0 | 533 | 80.5 |
|  |  | 1 | 129 | 19.5 |
|  | 19 | 0 | 533 | 80.5 |
|  |  | 1 | 129 | 19.5 |
|  | 20 | 0 | 536 | 81.0 |
|  |  | 1 | 126 | 19.0 |
|  | 21 | 0 | 535 | 80.8 |
|  |  | 1 | 127 | 19.2 |
|  | 22 | 0 | 538 | 81.3 |
|  |  | 1 | 124 | 18.7 |
|  | 23 | 0 | 537 | 81.1 |
|  |  | 1 | 125 | 18.9 |
|  | 24 | 0 | 536 | 81.0 |
|  |  | 1 | 126 | 19.0 |
|  | 25 | 0 | 534 | 80.7 |
|  |  | 1 | 128 | 19.3 |
|  | 26 | 0 | 530 | 80.1 |
|  |  | 1 | 132 | 19.9 |
|  | 27 | 0 | 533 | 80.5 |
|  |  | 1 | 129 | 19.5 |
|  | 28 | 0 | 547 | 82.6 |
|  |  | 1 | 115 | 17.4 |
|  | 29 | 0 | 526 | 79.5 |
|  |  | 1 | 136 | 20.5 |
|  | 30 | 0 | 541 | 81.7 |
|  |  | 1 | 121 | 18.3 |
|  | 31 | 0 | 529 | 79.9 |
|  |  | 1 | 133 | 20.1 |
|  | 32 | 0 | 527 | 79.6 |
|  |  | 1 | 135 | 20.4 |
|  | 33 | 0 | 538 | 81.3 |
|  |  | 1 | 124 | 18.7 |
|  | 34 | 0 | 533 | 80.5 |
|  |  | 1 | 129 | 19.5 |
|  | 35 | 0 | 533 | 80.5 |
|  |  | 1 | 129 | 19.5 |
|  | 36 | 0 | 537 | 81.1 |
|  |  | 1 | 125 | 18.9 |
|  | 37 | 0 | 530 | 80.1 |
|  |  | 1 | 132 | 19.9 |
|  | 38 | 0 | 542 | 81.9 |
|  |  | 1 | 120 | 18.1 |
|  | 39 | 0 | 534 | 80.7 |
|  |  | 1 | 128 | 19.3 |
|  | 40 | 0 | 534 | 80.7 |
|  |  | 1 | 128 | 19.3 |
|  | 41 | 0 | 540 | 81.6 |
|  |  | 1 | 122 | 18.4 |
|  | 42 | 0 | 539 | 81.4 |
|  |  | 1 | 123 | 18.6 |
|  | 43 | 0 | 537 | 81.1 |
|  |  | 1 | 125 | 18.9 |
|  | 44 | 0 | 537 | 81.1 |
|  |  | 1 | 125 | 18.9 |
|  | 45 | 0 | 536 | 81.0 |
|  |  | 1 | 126 | 19.0 |
|  | 46 | 0 | 537 | 81.1 |
|  |  | 1 | 125 | 18.9 |
|  | 47 | 0 | 541 | 81.7 |
|  |  | 1 | 121 | 18.3 |
|  | 48 | 0 | 526 | 79.5 |
|  |  | 1 | 136 | 20.5 |
|  | 49 | 0 | 527 | 79.6 |
|  |  | 1 | 135 | 20.4 |
|  | 50 | 0 | 526 | 79.5 |
|  |  | 1 | 136 | 20.5 |
